# miR-10a restores human mesenchymal stem cell differentiation by repressing KLF4

**DOI:** 10.1002/jcp.24402

**Published:** 2013-08-23

**Authors:** Jiao Li, Jun Dong, Zhen-hui Zhang, Dong-Cheng Zhang, Xiang-Yu You, Yun Zhong, Min-Sheng Chen, Shi-Ming Liu

**Affiliations:** 1 Department of Cardiology the Second Affiliated Hospital of Guangzhou Medical University, Guangzhou Institute of Cardiovascular DiseaseGuangzhou, China; 2 State Key Laboratory of Oncology in South China, Sun Yat-sen University Cancer CenterGuangzhou, China; 3 Department of Cardiovascular Surgery, The Second Affiliated Hospital of Guangzhou Medical University, Guangzhou Institute of Cardiovascular DiseaseGuangzhou, China

## Abstract

miRNAs have recently been shown to play a significant role in human aging. However, data demonstrating the effects of aging-related miRNAs in human mesenchymal stem cells (hMSCs) are limited. We observed that hMSC differentiation decreased with aging. We also identified that miR-10a expression was significantly decreased with age by comparing the miRNA expression of hMSCs derived from young and aged individuals. Therefore, we hypothesized that the downregulation of miR-10a may be associated with the decreased differentiation capability of hMSCs from aged individuals. Lentiviral constructs were used to up- or downregulate miR-10a in young and old hMSCs. Upregulation of miR-10a resulted in increased differentiation to adipogenic, osteogenic, and chondrogenic lineages and in reduced cell senescence. Conversely, downregulation of miR-10a resulted in decreased cell differentiation and increased cell senescence. A chimeric luciferase reporter system was generated, tagged with the full-length 3′-UTR region of KLF4 harboring the seed-matched sequence with or without four nucleotide mutations. These constructs were cotransfected with the miR-10a mimic into cells. The luciferase activity was significantly repressed by the miR-10a mimic, proving the direct binding of miR-10a to the 3′-UTR of KLF4. Direct suppression of KLF4 in aged hMSCs increased cell differentiation and decreased cell senescence. In conclusion, miR-10a restores the differentiation capability of aged hMSCs through repression of KLF4. Aging-related miRNAs may have broad applications in the restoration of cell dysfunction caused by aging. J. Cell. Physiol. 228: 2324–2336, 2013. © The Authors. Published by Wiley Periodicals, Inc.

Human mesenchymal stem cells (hMSCs) possess regeneration and differentiation capabilities (Pittenger et al., 1999; Jiang et al., 2002; Tateishi et al., 2008). However, previous studies indicate that cell proliferation, in vitro differentiation, colony-forming ability, cellular apoptosis, telomere function, and senescence of MSCs were all affected by the age of the donor (Fehrer and Lepperdinger, 2005; Fehrer et al., 2006; Sethe et al., 2006; Ju et al., 2007; Stolzing et al., 2008). The results of preclinical animal research also highlight the importance of donor cell age (Capogrossi, 2004; Lehrke et al., 2006; Dimmeler and Leri, 2008; Fan et al., 2010). The age of the donor is a key factor in hMSC function. Therefore, it is not surprising that the age of the donor also influences the outcome of hMSC therapy (Stolzing et al., 2008). Previous studies have demonstrated that age-related alterations of the donor cells combined with the endogenous responses of the aged recipients determine cell therapy results (Zhuo et al., 2010). Since age affects the efficacy of cell therapy using hMSCs, determining the mechanisms that control aging in these cells may prove beneficial in optimizing their utility in the treatment of degenerative conditions.

Accumulating data suggest that the expression levels of miRNA change during the process of aging. miRNAs participate in the processes of cell survival, replication, senescence, growth, and differentiation (Bartel, 2009; Noren Hooten et al., 2010). Evidence suggests that the expression of miRNA changes in the liver, lung, and brain of aged mice (Williams et al., 2007; Maes et al., 2008). Noren Hooten et al. (2010) compared miRNA expression in peripheral blood mononuclear cells from different aged populations and showed that nine miRNAs were upregulated in younger individuals. It has been shown that miR-371, miR-369-5P, miR-29c, miR-499, and let-7f are overexpressed in replicative-senescent hMSCs (Wagner et al., 2008). Thus, aging may affect miRNA expression patterns, and miRNA may influence the process of cell senescence (Bates et al., 2010; Li et al., 2011).

miRNA has also been implicated in MSC differentiation. In deed, Kim et al. (2009a,2009b) showed that miR-196a increases osteoblast differentiation through HOXC8, and miR-21 increases adipogenic differentiation through TGFBR2 in hMSCs derived from adipose tissue. Both miR-204 and miR-211 promote adipogenesis but inhibit MSC and mesenchymal progenitor cell osteogenesis by inhibiting the expression of Runx2 (Huang et al., 2010). In parallel, miR-140 increases chondrogenic differentiation of hMSCs through Sox9 and Col2a1 (Miyaki et al., 2009). miR-148b, -27a, and -489 regulate early osteogenic differentiation in hMSCs (Schoolmeesters et al., 2009). All these data suggest that miRNAs may regulate the differentiation process of MSCs.

Although previous studies identified age-related changes in miRNA expression, data confirming the effects of miRNA on the function of aged MSCs are limited. By comparing the miRNA expression profiles of hMSCs derived from young and old subjects, we identified four miRNAs (miR-196a, miR-378-star, miR-486-5p, and miR-664-star) that were significantly upregulated and three miRNAs (miR-10a, miR-708, and miR-3197) that were significantly downregulated in older individuals. Among these, we found that miR-10a showed the most dramatic change in aged hMSCs. We also observed that hMSC adipocyte, osteocyte, and chondrocyte differentiation was decreased with aging. Therefore, we hypothesized that miR-10a may play a major role in hMSC differentiation. In the current study, we investigated the regulatory role of miR-10a in hMSC differentiation related to age. The differentiation ability of MSCs is usually demonstrated by the three-lineage gold standard: adipocyte, osteoblast, and chondrocyte differentiation (Pittenger et al., 1999). Accordingly, we demonstrated that the differentiation of hMSCs to adipocytes, osteocytes, and chondrocytes decreased with aging.

## Materials and Methods

### Cell isolation, culture, and identification

This study was conducted in accordance with the Declaration of Helsinki and was approved by the Research Ethics Committee of Guangzhou Medical University. Human bone marrow was collected during cardiac valve surgery at the Second Affiliated Hospital of Guangzhou Medical University. Young hMSCs were obtained from 17- to 30-year-old patients, and old hMSCs from 65- to 80-year-old patients. All patients had valve disease, were of the same pathological status, and received the same medical treatment. Bone marrow was collected from 15 male and 15 female patients for each age group (N = 30/group). Three samples in each group were chosen for the microarray assays. Patient characteristics are presented in Table S1.

The hMSCs were cultured as previously described (Fan et al., 2010). Briefly, after centrifugation through a Ficoll–Paque gradient (1.077 g/ml density; GE Healthcare, Kretztechnik, Zipf, Austria), cells were separated and plated. After 48 h, non-adhesive cells were removed by changing the culture medium. The adhesive cells were harvested for passage when confluence reached approximately 80%. hMSCs were identified by flow cytometry after staining with antibodies against CD29, CD31, CD34, CD44, CD45, and CD166 (MultiSciences Biotech Co., Shanghai, China).

### Cell proliferation characteristics

The proliferative ability of hMSCs was analyzed with the CellTiter 96 AQueous Non-Radioactive Cell Proliferation Assay (Promega, Madison, WI) for seven consecutive days following the manufacturer's instructions. This assay is a colorimetric method for determining the number of proliferating cells. The assay uses a novel tetrazolium compound [3-(4,5-dimethylthiazol-2-yl)-5-(3-carboxymethoxyph-enyl)-2-(4-sulfophenyl)-2H-tetrazolium, inner salt; MTS] and an electron coupling reagent (phenazine methosulfate; PMS). MTS is bioreduced by proliferating cells into a formazan product that is soluble in culture medium. The absorbance of the formazan at 490 nm can be measured directly from 96-well plates without additional processing.

In brief, hMSCs were seeded into 96-well plates at a density of 4,000 cells/well, 20 µl of combined MTS and PMS solution was pipetted into each well containing 100 µl culture medium, and the cells were incubated for 4 h. An ELISA plate reader was used to measure the absorbance at 490 nm, and growth curves were generated. For each experiment, four samples from each group were tested in triplicate.

### Induction of cell differentiation

For adipocyte and osteoblast differentiation, hMSCs were seeded at 2 × 10^4^ cells/cm^2^ in culture medium. After incubation for 24 h, culture medium was replaced with adipocyte differentiation medium and osteoblast differentiation medium (Cat. no. A10070-01, A10072-01; Invitrogen, San Diego, CA). The differentiation medium was changed every 2 days for up to 10 days. According to the manufacturer (Invitrogen), the differentiation media contain the basal medium and supplemental reagents as provided by the manufacturer. Differentiated adipocytes were identified by Oil Red O staining. Alizarin Red S staining was used to estimate the extracellular matrix calcification. The positively stained area was quantified by ImageJ software (NIH, Bethesda, MD). Three fields were randomly chosen from each well. The mean percentage was determined from of a total of six wells. As noted previously, high-density pellet mass cultures were used for chondroblast differentiation (Tallheden et al., 2004). Briefly, 2 × 10^5^ hMSCs were cultured in a tube with 500 μl chondrogenic differentiation medium (Lonza, Basel, Switzerland) for 10 days. The differentiation medium was changed every 2 days.

### Senescence-activated β-galactosidase (SA-β-gal) staining

Aging hMSCs were identified with an SA-β-gal staining kit (Cell Signaling Technology, Boston, MA). Three fields were randomly chosen from each well and examined by light microscopy. The positively stained area was quantified by ImageJ (NIH). The mean percentage was determined from of a total of six wells.

### Quantitative real-time PCR (qRT-PCR) analysis

cDNA was synthesized from 1 μg of total RNA with a ReverTra Ace qPCR RT Kit (Toyobo Co., Ltd., Osaka, Japan). Real-time PCR was performed with Thunderbird SYBR® qPCR Mix (Toyobo Co., Ltd.). U6 small nuclear RNA and GAPDH mRNA were used as the references for miRNA and mRNA detection, respectively. miRNA and mRNA expression levels were determined by the ΔΔC_t_ method, which was used for the relative quantification of gene expression. miRNA RT primers were purchased from GenePharma (GenePharma, Shanghai, China). The primer sequences are presented in Table S2.

### Microarray assays

The miRNA expression of passage 2 hMSCs was analyzed using Affymetrix GeneChip 2.0 miRNA arrays as described previously (Wang et al., 2011). According to microarray assays conducted in previous studies, the results from three samples per group are considered statistically meaningful (Harris et al., 2008; Wagner et al., 2008; Wang et al., 2010). Therefore, three samples from each age group were used for the initial screening.

### Western blot analysis

Proteins were loaded onto SDS polyacrylamide gels (10%), electroblotted onto polyvinylidene difluoride membranes (PVDF; BioRad, Hercules, CA), and incubated with either a mouse monoclonal antibody (1E5) against KLF4 (1:400; Abcam, Cambridge, MA) or a monoclonal antibody against GAPDH (1:1,000; Santa Cruz Biotechnology, Santa Cruz, CA). Immunoreactive bands were detected with either an anti-rabbit or anti-mouse peroxidase-conjugated secondary antibody (1:2,000; Santa Cruz Biotechnology). Proteins were visualized by enhanced chemiluminescence (Amersham Pharmacia Biotech, Orsay, France). GAPDH was used as the loading control. For each experiment, hMSC samples from three young and three old donors were tested in triplicate.

### Plasmid construction

The 3′-UTR of KLF4 was subcloned into the pGL4.13 luciferase plasmid (Promega) to generate the pGL4.13-KLF4-3′UTR construct. By using overlapping extension PCR, we substituted four nucleotides in the KLF4 3′-UTR core binding sites to generate the pGL4.13-KLF4-3′UTR-mut construct. The primers used to clone the KLF4 3′-UTR and to generate the mutant are presented in Table S2.

### Luciferase reporter assay

A total of 100 ng of miR-10a mimic or scrambled control (GenePharma), 100 ng of pGL4.13-KLF4-3′UTR or pGL4.13-KLF4-3′UTR-mut containing the firefly luciferase reporter vector, and 5 ng of the control vector containing Renilla luciferase (pRL-TK) were used to transfect 6 × 10^4^ HEK-293T cells or 3 × 10^4^ hMSCs per well in a 48-well plate using 0.5 µl Lipofectamine 2000 (Invitrogen). hMSCs were transfected with 200 ng of miR-10a inhibitor or inhibitor scrambled control (GenePharma), 200 ng of pGL4.13-KLF4-3′UTR or pGL4.13-KLF4-3′UTR-mut, and 10 ng of pRL-TK using 1 µl Lipofectamine 2000 (Invitrogen). Renilla and firefly luciferase activities were measured 48 h after transfection with the Dual-Luciferase system (Promega). The sequences for the miR-10a mimic and inhibitor are listed in Table S3.

### Viral vector construction and transduction

Lentiviral constructs for overexpression of miR-10a (LV-miR-10a), KLF4 (LV-KLF4), or inhibition of miR-10a and KLF4 (LV-anti-10a and LV-anti-KLF4) were purchased from GenePharma. Diluent viral solution and polybrene (5 mg/ml) were added to the culture medium to transduce hMSCs for 72 h. The lentiviral sequences are listed in Table S3. To construct LV-KLF4, the entire CDS region of KLF4 (NCBI reference sequence: NM_004235.4) was subcloned into the lentiviral vector. In the rescue experiment, after confirming that LV-miR-10a effectively increased miR-10a expression and inhibited KLF4 expression in hMSCs, the miR-10a-overexpressing hMSCs were infected with LV-KLF4 to restore the expression of KLF4.

### Statistical analyses

The data are presented as mean ± SD. All statistical analyses were performed with SPSS 16.0. Comparisons between groups were analyzed using two-sided *t*-tests or ANOVA for more than two groups. A value of *P* < 0.05 was defined as statistically significant.

## Results

### In vitro differentiation of hMSCs

**Figure 1:**
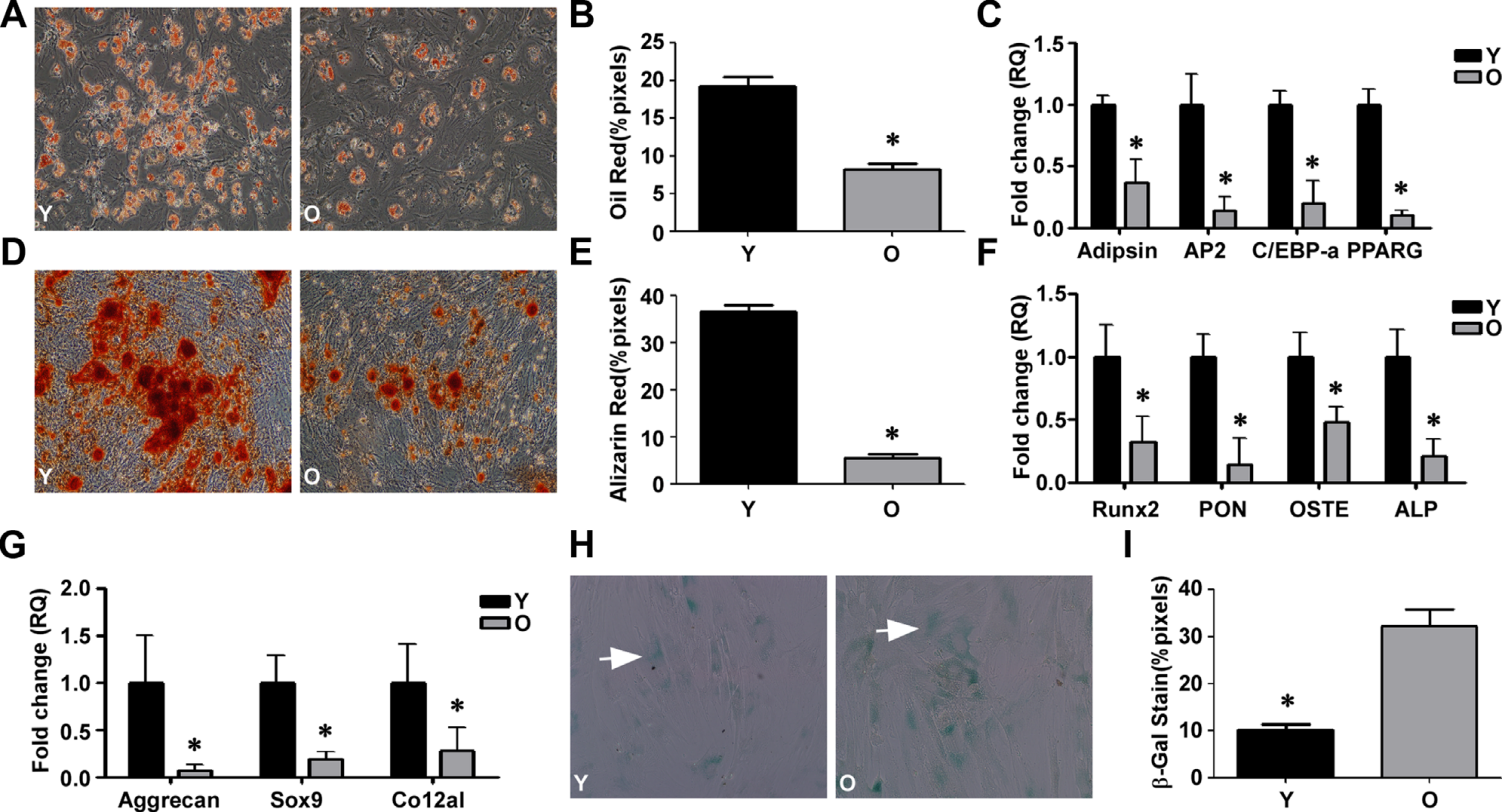
In vitro hMSC differentiation. Young (Y) and old (O) hMSCs were differentiated to adipogenic, osteogenic, and chondrogenic cells. A,B: Oil Red O staining and quantification of adipogenic differentiation (200× magnification). C: The adipocyte-specific genes adipsin, AP2, C/EBP-α, and PPARG were quantified by qRT-PCR in Y and O hMSCs. D,E: Alizarin Red S staining and quantification of osteogenic differentiation (100× magnification). F: The osteogenic-specific genes Runx2, PON, OSTE, and ALP were quantified by qRT-PCR in Y and O hMSCs. G: The chondrogenic-specific genes Aggrecan, Sox9 and Co12a1 were quantified by qRT-PCR in Y and O hMSCs. H,I: Representive images of SA-β-gal staining (arrows indicate positive cells) and quantification of cell senescence. The data represent mean ± SD (n = 5/group). *P < 0.05 Y versus O hMSCs.

To determine whether hMSC differentiation is altered with age, we obtained bone marrow MSCs from young (17- to 30-year-old) and old (65- to 80-year-old) patients. Adipogenic, osteogenic, and chondrogenic differentiation were induced by standard protocols. Adipocyte and osteoblast differentiation were evaluated by Oil Red O or Alizarin Red S staining, respectively, and were significantly higher in young hMSCs (Fig. 1A,B,D,E). To quantitatively compare the cells from young and old donors, qRT-PCR was used to detect the expression of genes related to the three cell lineages. The attenuated adipogenic differentiation of aged hMSCs was further evidenced by the reduced expression of adipsin, AP2, C/EBP-α, and PPARG genes (Fig. 1C). The expression of Runx2, osteopontin (PON), osteocalcin (OSTE), and alkaline phosphatase (ALP) genes were lower in old hMSCs, indicating that aging led to an attenuation of osteogenic differentiation (Fig. 1F). The chondrogenic pellets formed by old hMSCs were smaller and looser than those formed by hMSCs obtained from young patients (data not shown), indicating less chondrogenic differentiation. The expression of the chondrogenesis-specific genes aggrecan, Sox9, and Co12al was also decreased in the old cells, indicating a reduction in cartilage formation (Fig. 1G). Therefore, the differentiation potential of old hMSCs was decreased. In addition to differentiation, the proliferative ability of old hMSCs was also impaired compared with that of the young hMSCs (Fig. S1A).

### Senescence and immunophenotype of hMSCs

The senescence biomarker SA-β-gal is used to investigate the functional implications of aging in hMSCs (Dimri et al., 1995). Figure 1H,I shows that more old than young hMSCs stained positive for SA-β-gal.

Next, we determined if aging affected the immunophenotype of hMSCs by evaluating the six classic cell surface antigens used to identify MSCs (Horwitz et al., 2005; Wagner et al., 2005). Both young and old hMSCs were positive for CD29, CD44, and CD166 but negative for CD31, CD34, and CD45 (Fig. S2). These results indicate that although old hMSCs became senescent, the expression and composition of hMSC-specific surface markers did not change.

### Alternation of miRNA expression profiles in aged hMSCs

**Figure 2:**
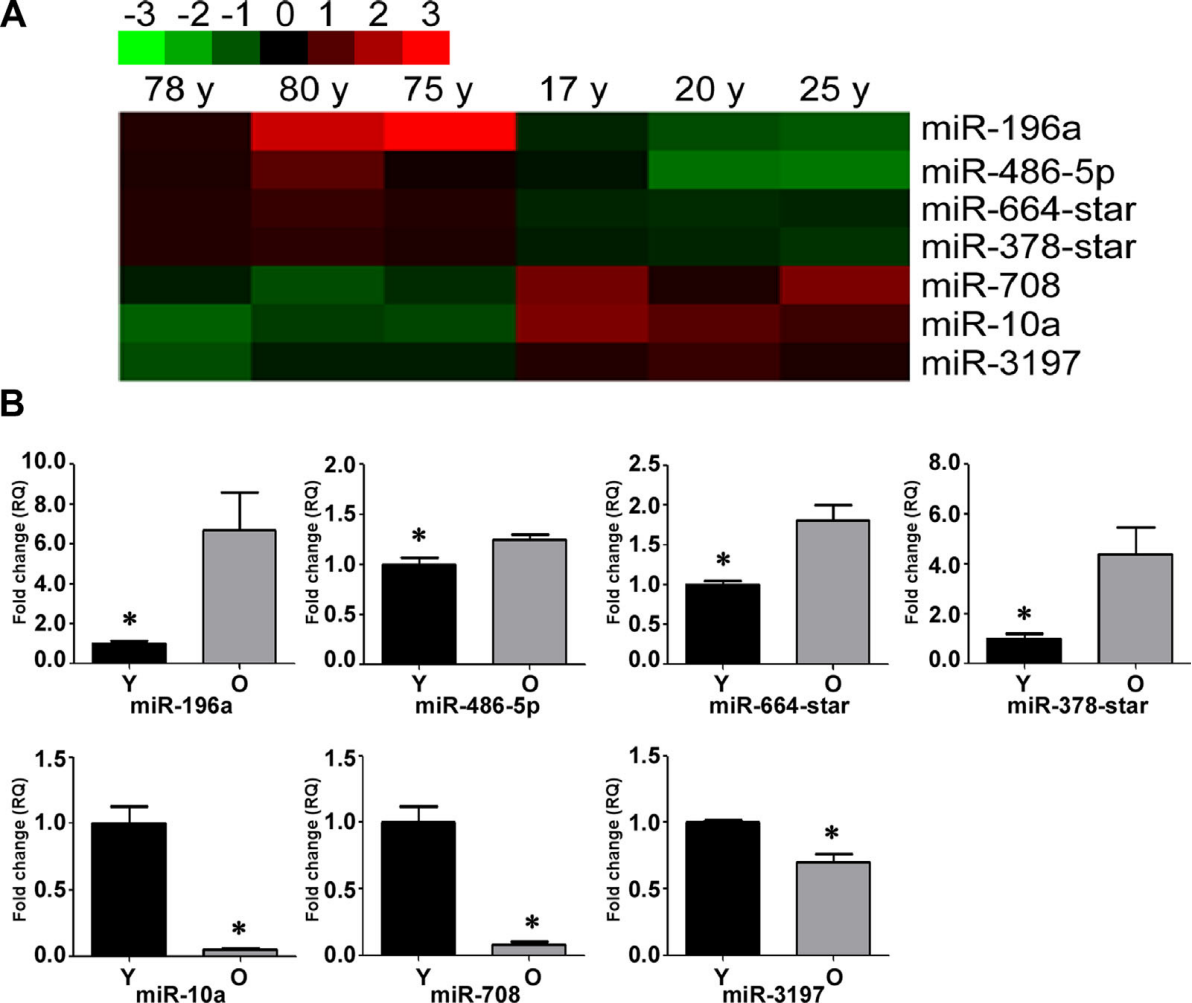
Alternation of miRNA expression profiles in aged hMSCs. A: Differential miRNA expression in young (Y) and old (O) hMSCs was determined by microarray analysis. B: Differential miRNA expression was validated by qRT-PCR. miR-196a, miR-486-5p, miR-664-star, and miR-378-star were upregulated, and miR-10a, miR-708, and miR-3197 were downregulated in old versus young hMSCs. The data represent mean ± SD (n = 3–6/group). *P < 0.05 Y versus O hMSCs.

We compared the hMSC miRNA expression profiles of three young (17-year-old female, 20-year-old male, and 25-year-old male) and three old (78-year-old female, 75-year-old male, and 80-year-old male) individuals using miRNA arrays (Table S4). Using significance analysis of microarray (SAM) software, we identified seven differentially expressed miRNAs (Fig. 2A, Table S5). Next, qRT-PCR was used to validate the microarray data for the miRNAs of interest. Notably, miR-196a, miR-486-5p, miR-664-star, and miR-378-star were all significantly upregulated, and miR-10a, miR-708, and miR-3197 were downregulated in the hMSCs from old subjects compared with young subjects (Fig. 2B). Furthermore, we found that miR-10a showed the most dramatic decrease with age.

### Effects of miR-10a on hMSC differentiation

**Figure 3:**
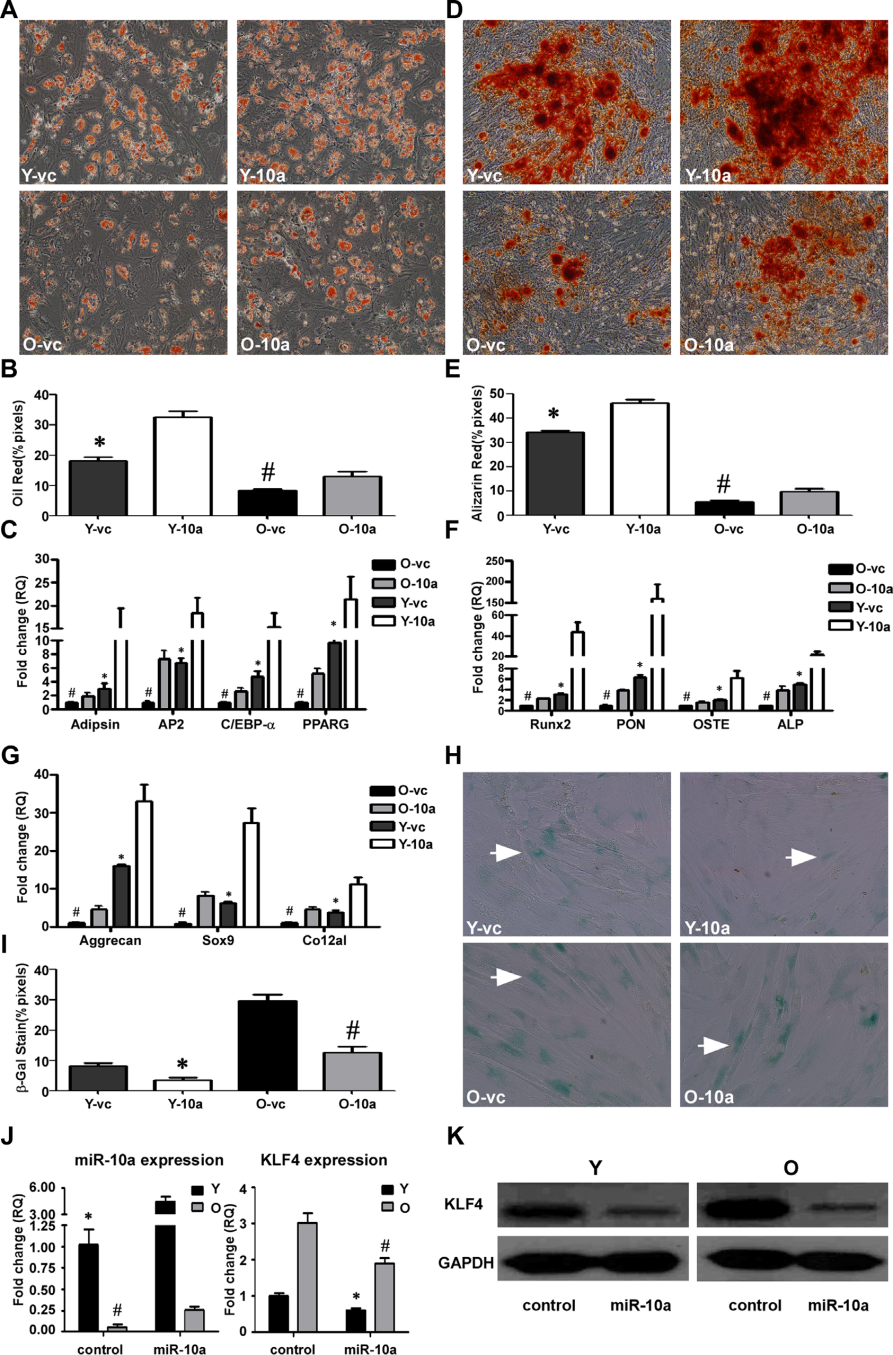
Effects of miR-10a on hMSC differentiation. LV-miR-10a or LV-control was used to transduce young (Y) and old (O) hMSCs (Y-10a, O-10a and Y-vc, O-vc). The transduced hMSCs were used to induce cell differentiation. A,B: Oil Red O staining and quantification of adipogenic differentiation in transduced Y and O hMSCs (200× magnification). C: The adipocyte-specific genes adipsin, AP2, C/EBP-α and PPARG were quantified by qRT-PCR. D,E: Alizarin Red S staining and quantification of osteogenic differentiation in transduced Y and O hMSCs (100× magnification). F: The osteogenic-specific genes Runx2, PON, OSTE, and ALP were quantified by qRT-PCR. G: The chondrogenic-specific genes Aggrecan, Sox9, and Co12a1 were quantified by qRT-PCR in transduced Y and O hMSCs. H,I: SA-β-gal staining (arrows indicate positive cells) and quantification of cell senescence in transduced Y and O hMSCs. J: The expression level of miR-10a and KLF4 was quantified by qRT-PCR in transduced Y and O hMSCs. K: KLF4 protein expression in transduced Y and O hMSCs. The data represent mean ± SD (n = 3–4/group). *P < 0.05 Y-10a versus Y-vc; ^#^*P* < 0.05 O-10a versus O-vc.

To investigate the impact of miR-10a on hMSC differentiation, we produced a lentiviral construct to overexpress miR-10a (LV-miR-10a). Adipogenic, osteogenic, and chondrogenic cell lineages were induced from young and old hMSCs overexpressing miR-10a. LV-miR-10a transduction significantly increased the differentiation of the adipogenic and osteogenic lineages, especially in the young cells overexpressing miR-10a, as demonstrated by Oil Red O (Fig. 3A,B) and Alizarin Red S staining (Fig. 3D,E). Moreover, overexpression of miR-10a partially restored differentiation to the three lineages in the old hMSCs. The qRT-PCR results revealed that after differentiation, the expression of adipogenic-related genes (adipsin, AP2, C/EBP-α, PPARG; Fig. 3C), osteogenic-related genes (Runx2, PON, OSTE, ALP; Fig. 3F), and chondrogenic-related genes (aggrecan, sox9, and co12al; Fig. 3G) was upregulated to a greater extent in both LV-miR-10a young and old hMSCs compared with the respective LV-control cells.

After transducing the hMSCs with LV-miR10a, we compared the continuous 7-day proliferation rate of the hMSCs. The results of this assay indicated that the LV-miR-10a-transduced cells in both the young and old groups grew at a slower rate compared with that of the respective LV-control-transduced cells (Fig. S1B). To detect cell senescence, virus-transduced cells were stained with SA-β-gal. Notably, there were fewer SA-β-gal positive cells among the LV-miR10a groups compared to the LV-control groups, indicating that LV-miR10a decreased hMSC senescence in both young and old hMSCs (Fig. 3H,I). Real-time PCR analysis demonstrated that transduction of hMSCs by LV-miR-10a upregulated the expression of miR-10a 4.5-fold (Fig. 3J).

### Downregulation of miR-10a suppresses the differentiation potential of hMSCs

**Figure 4:**
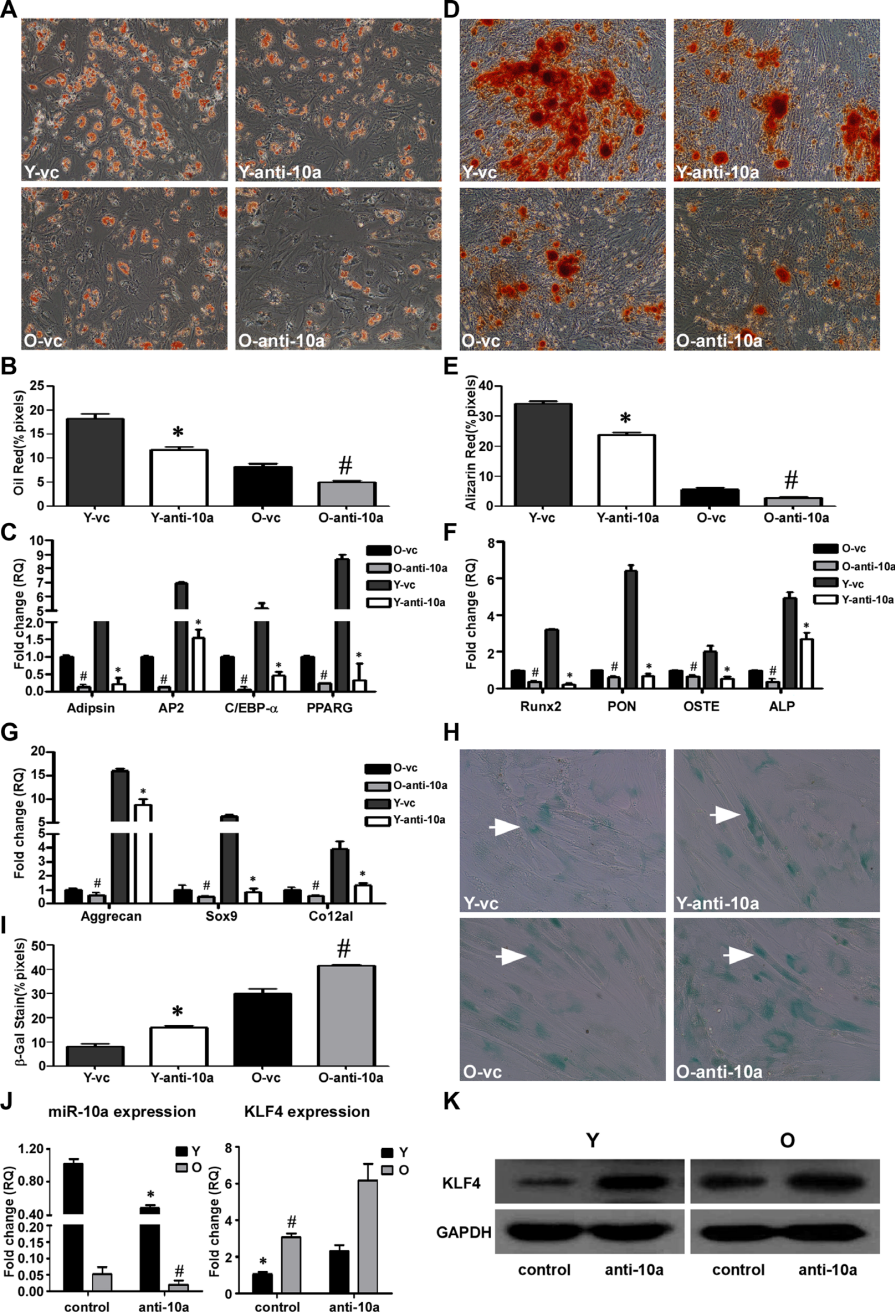
Downregulation of miR-10a suppresses hMSC differentiation potential. LV-anti-10a or LV-control was used to transduce young (Y) and old (O) hMSCs (Y-anti-10a, O-anti-10a and Y-vc, O-vc). The transduced hMSCs were used to induce cell differentiation. A,B: Oil Red O staining and quantification of adipogenic differentiation in transduced Y and O hMSCs (200× magnification). C: The adipocyte-specific genes adipsin, AP2, C/EBP-α, and PPARG were quantified by qRT-PCR. D,E: Alizarin Red S staining and quantification of osteogenic differentiation in transduced Y and O hMSCs (100× magnification). F: The osteogenic-specific genes Runx2, PON, OSTE, and ALP were quantified by qRT-PCR. G: The chondrogenic-specific genes Aggrecan, Sox9, and Co12a1 were quantified by qRT-PCR in transduced Y and O hMSCs. H,I: SA-β-gal staining (arrows indicate positive cells) and quantification of cell senescence in transduced Y and O hMSCs. J: The expression level of miR-10a and KLF4 was quantified by qRT-PCR in transduced Y and O hMSCs. K: KLF4 protein expression in transduced Y and O hMSCs. The data represent mean ± SD (n = 3–4/group). *P < 0.05 Y-anti-10a versus Y-vc; ^#^*P* < 0.05, O-anti-10a versus O-vc.

Next, to further prove the association of miR-10a with cell differentiation, a lentiviral construct to inhibit miR-10a expression (LV-anti-10a) was produced. LV-anti-10a-transduced young and old hMSCs were differentiated into adipogenic, osteogenic, and chondrogenic cells. Oil Red O staining (Fig. 4A,B) and Alizarin Red S staining (Fig. 4D,E) showed decreased adipogenic and osteogenic differentiation in both the LV-anti-10a young and old hMSCs compared with that of the respective LV-control cells. As expected, the differentiation-related genes were all downregulated to a greater extent in the LV-anti-10a-infected hMSCs compared with the LV-control-infected cells (Fig. 4C,F,G).

In the cell proliferation assay, LV-anti-10a-transduced young and old hMSCs exhibited higher proliferative ability than that of the respective hMSCs transduced by the control lentivirus (Fig. S1C). However, downregulation of miR-10a expression increased the percentage of SA-β-gal positive cells among the LV-anti-10a-infected groups compared to the LV-control-infected groups, indicating that repression of miR-10a increased hMSC senescence in both young and old hMSCs (Fig. 4H,I). The data from qRT-PCR demonstrated that miR-10a was effectively inhibited by transducing the hMSCs with LV-anti-miR10a (Fig. 4J).

### miR-10a targets KLF4 mRNA

**Figure 5:**
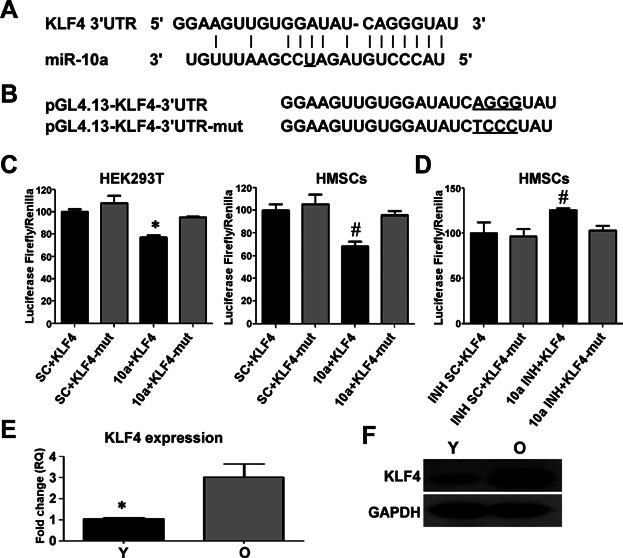
miR-10a targets the 3′-UTR of KLF4 mRNA. A: miR-10a sequences and the binding sites in KLF4 3′-UTR. B: pGL4.13-KLF4-3′UTR and pGL4.13-KLF4-3′UTR-mut sequences. C: HEK 293T cells and hMSCs were cotransfected with a miR-10a mimic or a scrambled control (SC) and pGL4.13-KLF4-3′UTR or pGL4.13-KLF4-3′UTR-mut. Renilla and firefly luciferase activities were measured 48 h after transfection. The luciferase activity for KLF4 3′-UTR was normalized to endogenous renilla luciferase. D: hMSCs were cotransfected with a miR-10a inhibitor or a scrambled control and pGL4.13-KLF4-3′UTR or pGL4.13-KLF4-3′UTR-mut. The luciferase activity for KLF4 3′-UTR was measured 48 h after transfection and normalized to endogenous renilla luciferase. E,F: The basal mRNA and protein expression of KLF4 in Y and O hMSCs. The data represent mean ± SD (n = 3–6/group). *P < 0.05 10a + KLF4 versus SC + KLF4, Y versus O hMSCs; ^#^*P* < 0.05 INH + KLF4 versus INH SC + KLF4.

A bioinformatics approach, incorporating sequence matching and mRNA secondary structure, was employed to predict mRNA targets (www.targetscan.org). KLF4 appeared most likely to be the major candidate because the miR-10a seed sequence is reverse complementary to the seed-matched sequence in the 3′-UTR region of human KLF4 (Fig. 5A). Therefore, KLF4 was chosen as the potential downstream target of miR-10a. To confirm the bioinformatics prediction, a chimeric luciferase reporter system was generated, tagged with the full-length 3′-UTR region of KLF4 harboring the seed-matched sequence with four nucleotide mutations (pGL4.13-KLF4-3′UTR-mut) or without (pGL4.13-KLF4-3′UTR; Fig. 5B). These constructs were cotransfected with the miR-10a mimic into HEK 293 cells. As shown in Figure 5C, luciferase activity was significantly repressed by the miR-10a mimic, proving the direct binding of the miR-10a to the 3′-UTR of KLF4. A similar effect was also observed in the hMSCs (Fig. 5C). In contrast, cotransfection of hMSCs with a miR-10a inhibitor and the pGL4.13-KLF4-3′-UTR construct increased luciferase activity (Fig. 5D) compared with the groups cotransfected with the inhibitor and pGL4.13-KLF4-3′UTR-mut. Therefore, we believe that miR-10a can restore the differentiation capabilities of aged hMSCs through the repression of KLF4 expression.

### miR-10a regulates endogenous KLF4 protein expression

Contrary to the miR-10a expression results, the endogenous KLF4 mRNA and protein expression were much higher in the old than the young hMSCs (Fig. 5E,F). To determine if miR-10a would affect endogenous KLF4 expression, we compared KLF4 expression after infecting the hMSCs with LV-miR-10a and LV-anti-10a. Upregulation of miR-10a repressed both the endogenous mRNA and protein expression of KLF4 (Fig. 3J,K). By contrast, downregulation of miR-10a increased endogenous KLF4 mRNA and protein levels (Fig. 4J,K).

### Inhibition of KLF4 enhances hMSC differentiation

**Figure 6:**
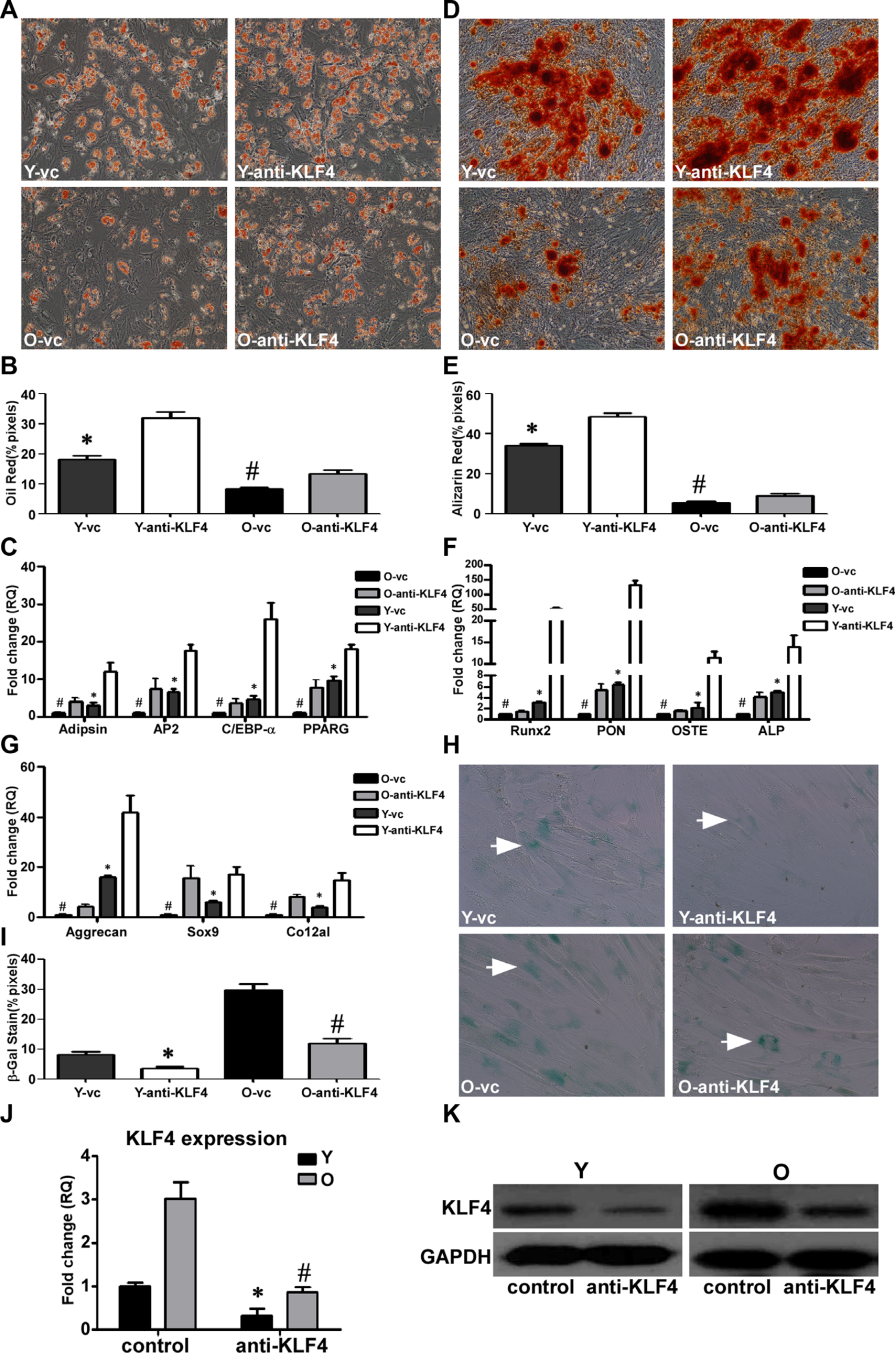
Inhibition of KLF4 enhances hMSC differentiation potential. LV-anti-KLF4 or LV-control was used to transduce young (Y) and old (O) hMSCs (Y-anti-KLF4, O-anti-KLF4 and Y-vc, O-vc). The transduced hMSCs were used to induce cell differentiation. A,B: Oil Red O staining and quantification of adipogenic differentiation in transduced Y and O hMSCs (200× magnification). C: The adipocyte-specific genes adipsin, AP2, C/EBP-α, and PPARG were quantified by qRT-PCR. D,E: Alizarin Red S staining and quantification of osteogenic differentiation in transduced Y and O hMSCs (100× magnification). F: The osteogenic-specific genes Runx2, PON, OSTE, and ALP were quantified by qRT-PCR. G: The chondrogenic-specific genes Aggrecan, Sox9, and Co12a1 were quantified by qRT-PCR. H,I: SA-β-gal staining (arrows indicate positive cells) and quantification of cell senescence in transduced Y and O hMSCs. J,K: KLF4 gene and protein expression in transduced Y and O hMSCs. The data represent mean ± SD (n = 3–4/group). *P < 0.05 Y-anti-KLF4 versus Y-vc; ^#^*P* < 0.05 O-anti-KLF4 versus O-vc.

To confirm the effect of KLF4 on hMSCs, we inhibited KLF4 expression in hMSCs using the lentiviral construct LV-anti-KLF4. Young and old hMSCs were transduced with LV-anti-KLF4 and differentiated into adipogenic, osteogenic, and chondrogenic cell lineages. Oil Red O staining (Fig. 6A,B) and Alizarin Red S staining (Fig. 6D,E) confirmed that suppression of KLF4 expression increased hMSC differentiation to adipogenic and osteogenic cells in both the young and the old hMSCs compared with the respective non-suppressed groups. As expected, the differentiation-related genes were all upregulated to a greater extent in both the LV-anti-KLF4 young and old hMSCs compared with the respective LV-control cells (Fig. 6C,F,G).

The impact of LV-anti-KLF4 on hMSC growth was assessed with a cell proliferation assay. Proliferation was decreased in LV-anti-KLF4-transduced hMSCs compared with that of the LV-control-transduced hMSCs (Fig. S1D). There were fewer SA-β-gal positive cells among the LV-anti-KLF4 hMSCs compared to the respective LV-control cells, indicating that inhibition of KLF4 expression decreased hMSC senescence in both young and old hMSCs (Fig. 6H,I). The data from qRT-PCR and Western blot indicated that LV-anti-KLF4 effectively inhibited KLF4 expression in hMSCs (Fig. 6J,K).

### miR-10a-induced hMSC differentiation is attenuated by the restoration of KLF4

**Figure 7:**
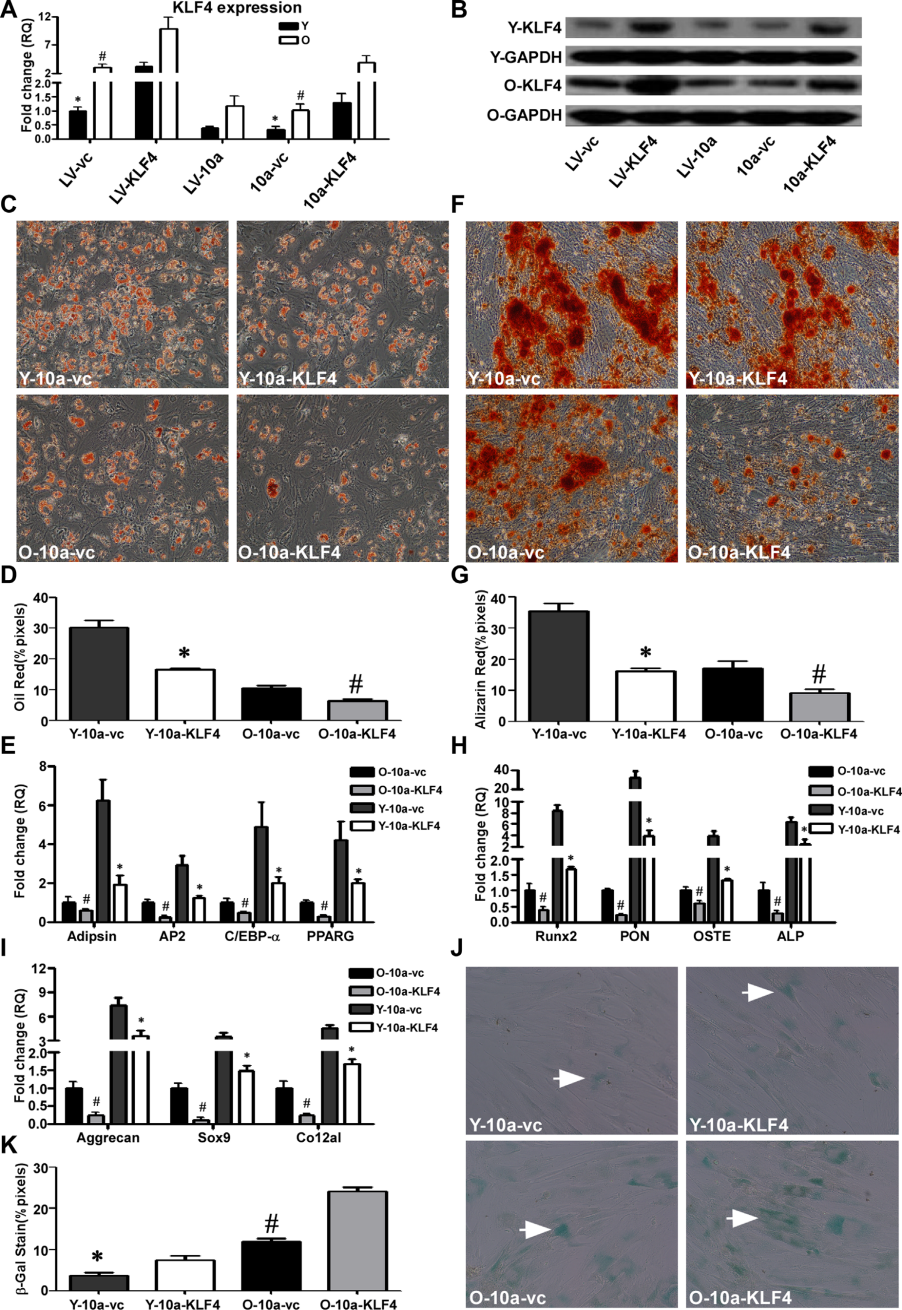
miR-10a-induced hMSC differentiation is attenuated by the restoration of KLF4. KLF4 gene (A) and protein (B) expression in LV-KLF4 or LV-control infected young (Y) and old (O) hMSCs (LV-KLF4, LV-vc) as well as LV-KLF4 or LV-control infected Y and O hMSCs overexpressing miR-10a (10a-KLF4, 10a-vc). LV-control infected Y hMSCs were set as the baseline. C,D: Oil Red O staining and quantification of adipogenic differentiation in infected Y and O hMSCs (200× magnification). E: The adipocyte-specific genes adipsin, AP2, C/EBP-α, and PPARG were quantified by qRT-PCR in infected Y and O hMSCs. F,G: Alizarin Red S staining and quantification of osteogenic differentiation in infected Y and O hMSCs (100× magnification). H: The osteogenic-specific genes Runx2, PON, OSTE, and ALP were quantified by qRT-PCR in infected Y and O hMSCs. I: The chondrogenic-specific genes Aggrecan, Sox9, and Co12a1 were quantified by qRT-PCR in infected Y and O hMSCs. J,K: SA-β-gal staining (arrows indicate positive cells) and quantification of cell senescence in infected Y and O hMSCs. The data represent mean ± SD (n = 3–4/group). *P < 0.05 Y-LV-KLF4 versus Y-LV-vc, Y-10a-KLF4 versus Y-10a-vc; ^#^*P* < 0.05 O-LV-KLF4 versus O-LV-vc, O-10a-KLF4 versus O-10a-vc.

To further confirm that miR-10a induces hMSC differentiation and reduces cell senescence through inhibition of KLF4, we designed a rescue experiment in which KLF4 expression was restored in hMSCs overexpressing miR-10a. Real-time PCR and Western blot analyses demonstrated that transduction of hMSCs with LV-KLF4 increased KLF4 expression (Fig. 7A,B). We then infected the miR-10a-overexpressing young and old hMSCs with LV-KLF4, resulting in the restoration of KLF4 expression (Fig. 7A,B). The cells were subsequently analyzed for differentiation into the adipogenic, osteogenic, and chondrogenic lineages. We found decreased adipogenic and osteogenic differentiation in both young and old KLF4-overexpressing hMSCs (Fig. 7C,D,F,G). Again, qRT-PCR was used to quantify the expression of the lineage-specific marker genes (Fig. 7E,H,I). The results clearly indicated that the adipogenic, osteogenic, and chondrogenic differentiation of miR-10a-overexpressing hMSCs was counteracted by the restoration of KLF4.

Both cell proliferation (Fig. S1E) and senescence (Fig. 7J,K) were higher in the young and old KLF4-restored groups compared to that of the respective control groups.

## Discussion

To enhance the understanding of aging-related miRNAs in hMSC differentiation, this study compared miRNA expression profiles of hMSCs derived from young and old subjects and found that miR-10a was the most significantly altered with aging. We also observed that hMSC adipocyte, osteocyte, and chondrocyte differentiation was decreased with aging. Upregulation of miR-10a restored the differentiation ability of aged hMSCs and reduced cell senescence, but cell growth was inhibited. KLF4 was chosen as the potential downstream target of miR-10a by miRNA target prediction. To confirm the bioinformatics prediction, a chimeric luciferase reporter system was used to prove the direct binding of miR-10a to the 3′-UTR of KLF4. Up- or downregulation of miR-10a and KLF4 showed that miR-10a can restore the differentiation capabilities of aged hMSCs through the repression of KLF4 expression.

The differentiation capacity of stem cells is an important characteristic for the repair and regeneration of tissues/organs. Consistent with previous studies, the data presented here demonstrate that the differentiation capacity of young hMSCs is superior to that of old cells (O'Driscoll et al., 2001; Baxter et al., 2004; Kretlow et al., 2008; Zhang et al., 2008). Along with the decrease in differentiation capacity, the old hMSCs proliferated more slowly and became senescent, which may be caused by telomere shortening or a loss of telomere function (Mendes et al., 2002; Baxter et al., 2004).

Accompanying the reduction in differentiation, the expression of miR-10a was significantly downregulated in aged hMSCs, implying that miR-10a may play an active role in the aging-related changes in the differentiation ability of hMSCs. Indeed, Foley et al. found that by repressing nuclear receptor corepressor 2 (NCOR2), which induces transcriptional alterations, miR-10a/b induced neuroblastoma cell differentiation similar to all-trans-retinoic acid. Using a luciferase reporter system, miR-10a/b was proven to target the 3′-UTR of NCOR2. Direct suppression of NCOR2 also induces neural cell differentiation. Thus, miR-10a/b promote neuroblastoma differentiation by suppressing NCOR2 expression (Foley et al., 2011). All these results suggest that miR-10a may be a key regulator for MSC differentiation. However, miR-10a may induce MSCs to a variety of cell types by modulating different downstream targets. As multipotent cells, MSCs have the potential to differentiate to many cell types besides adipocytes, osteoblasts, and chondrocytes, and this should be investigated in future studies.

Unlike miR-10a, no significant difference was found in the miR-10b expression level between the young and old groups (Fig. S3A). Consistent with previous data (Tian et al., 2010), we also found that similar to miR-10a, miR-10b also targets the 3′-UTR of KLF4 (Fig. S3B–D). However, compared to miR-10a, the relative abundance of miR-10b was much lower, particularly in young hMSCs (Fig. S3E). In agreement with the current data, Fang *et al*. have also shown that the basal level of miR-10b is much lower than that of miR-10a in swine aortic endothelium cells and human aortic endothelial cells (Fang et al., 2010).

Investigation of the underlying molecular mechanisms responsible for miR-10a mediated hMSC differentiation showed that miR-10a can restore the differentiation capabilities of aged hMSCs through the repression of KLF4 expression. KLF4, as a downstream mediator, can either repress or activate transcription and participate in cell cycle regulation and differentiation (Shields et al., 1996; Zhang et al., 2000). KLF4 has been proven to prevent embryonic stem cell (ESC) differentiation through either LIF or BMP4, and, in ESC renewal, KLF4 functions upstream to Nanog (Zhang et al., 2010). To demonstrate the effect of KLF4 in hMSC differentiation, we first confirmed that KLF4 levels were elevated in the old hMSCs, in parallel with the functional decline in cell differentiation. Through the luciferase reporter analysis, we found that miR-10a directly targets KLF4 mRNA at the 3′-UTR. In addition, lentiviral-mediated overexpression of miR-10a can decrease KLF4. Direct blockage of KLF4 expression promotes cell differentiation and reduces cell aging in hMSCs. Furthermore, after the restoration of KLF4 expression in miR-10a-overexpressing hMSCs, miR-10a could no longer increase cell differentiation and reduce cell senescence. Together, these data confirm that miR-10a induces hMSC differentiation and reduces cell senescence by direct repression of KLF4.

miR-10a is located in the HOXB gene cluster on chromosome 17q21. During mouse embryogenesis, miR-10a/10b are expressed after the gene cluster (Mansfield et al., 2004; Tanzer et al., 2005). HOX genes function as transcriptional regulators and modulate MSC behavior. Hoxb2, Hoxb5, Hoxb7, and Hoxc4 expression is associated with the expansion and self-renewal of MSCs, whereas other Hox family members, including HOXA7, HOXB3, HOXA3, and HOXB13, have been implicated in regulating endothelial differentiation of hMSCs (Phinney et al., 2005). Previous data indicate that during in vitro hMSC differentiation, HOXA7 and HOXB3 expression increases but HOXA3 and HOXB13 expression decreases (Chung et al., 2009). To determine if the change in miR-10a expression in aged hMSCs was associated with a change in HOXB genes, we examined the expression of HOXB3 in young and old hMSCs. As presented in Figure S3F, HOXB3 expression was decreased in the old hMSCs, corresponding with the change in miR-10a expression. These data are consistent with other studies showing that miR-10a expression is altered along with that of HOXB4 and HOXB5 during CD34^+^ hematopoietic progenitor differentiation to megakaryocytes (Garzon et al., 2006).

The aberrant expression of miR-10a/10b is also implicated in various human diseases, particularly several tumors. In human breast cancer cells, miR-10a can repress the expression of Hoxd4 by a transcriptional mechanism, thus demonstrating the function of miR-10a in the regulation of human genes (Tan et al., 2009). Repression of miR-10a can increase the upstream stimulatory factor (USF2) expression and promote the proliferation of chronic myeloid leukemia cells (Agirre et al., 2008). Contrary to being increased in human pancreatic adenocarcinomas and glioblastoma multiforme (Ciafre et al., 2005; Bloomston et al., 2007), miR-10b has been found to be decreased in breast cancer tissues (Ma et al., 2007). MiR-10b levels have been reported to be repressed by heparin; however, they are increased by thrombin in human microvascular endothelial cells (Shen et al., 2011). In future studies, the function of miR-10a in angiogenesis should be investigated.

In conclusion, we demonstrated that miR-10a was significantly decreased in old hMSCs and that this reduction was related to the impaired adipogenic, osteogenic, and chondrogenic differentiation of the cells. We determined that miR-10a directly and functionally targets KLF4 mRNA. Furthermore, we demonstrated that miR-10a can restore hMSC differentiation and can reduce cell senescence by direct suppression of KLF4 expression. Further studies on the effects of miR-10a on the regenerative capacity of hMSCs are needed.

## Supporting Information

**Fig. S1. In vitro proliferation. Seven-day growth curves are presented for young (Y) and old (O) hMSCs.** A: Non-transduced hMSCs. B: LV-miR-10a-transduced hMSCs. C: LV-anti-10a-transduced hMSCs. D: LV-anti-KLF4-transduced hMSCs. E: LV-KLF4 and miR-10a cotransduced hMSCs. The data represent mean ± SD (n = 4/group). **P* < 0.05 Y versus O hMSCs; Y-10a, Y-anti-10a or Y-anti-KLF4 versus Y-vc; Y-10a-KLF4 versus Y-10a-vc. ^#^*P* < 0.05 O-10a, O-anti-10a or O-anti-KLF4 versus O-vc; O-10a-KLF4 versus O-10a-vc.

**Fig. S2. hMSC surface antigen expression.** Flow cytometric analysis of cell surface antigens shows that hMSCs are positive for CD44, CD29, and CD166 (A) and negative for CD31, CD34, and CD45 (B) in both young (Y) and old (O) hMSCs. The data represent mean ± SD (n = 4/group).

**Fig. S3. Relative expression of miR-10a and miR-10b.** A: miR-10b expression in young (Y) and old (O) hMSCs. B: miR-10b binding site at the 3′-UTR of KLF4. C: hMSCs were cotransfected with a miR-10b mimic or a scrambled control and pGL4.13-KLF4-3′UTR or pGL4.13-KLF4-3′UTR-mut. Renilla and firefly luciferase activities were measured 48 h after transfection. The luciferase activity for KLF4 3′-UTR was normalized to endogenous renilla luciferase. D: hMSCs were cotransfected with a miR-10b inhibitor or a scrambled control and pGL4.13-KLF4-3′UTR or pGL4.13-KLF4-3′UTR-mut. Renilla and firefly luciferase activities were measured 48 h after transfection. The luciferase activity for KLF4 3′-UTR was normalized by the endogenous renilla luciferase. E: The basal expression levels of miR-10a and miR-10b in Y and O hMSCs. F: The basal expression levels of HOXB3 in Y and O hMSCs. The data represent mean ± SD (n = 3–4/group). **P* < 0.05 10b + KLF4 versus SC + KLF4, Y-10a versus Y-10b, Y-HOXB3 versus O-HOXB3. ^#^*P* < 0.05 10b INH + KLF4 versus INH SC + KLF4, O-10a versus O-10b.

**Table S1.** Patient demographic information.

**Table S2.** miRNA RT primer and qRT-PCR primer sequences.

**Table S3.** The sequences of the lentivirus and the miR-10a and miR-10b mimics and inhibitors.

**Table S4.** miRNA expression of hMSCs of different ages.

**Table S5.** Differential miRNA expression of hMSCs of different ages.

**Method S1.** Luciferase reporter assay of miR-10b.
