# Supplementary figures and images for: miR-10a restores human mesenchymal stem cell differentiation by repressing KLF4
Source: J Cell Physiol. 2013 Aug 23;228(12):2324–36. doi: 10.1002/jcp.24402 (PMC4285942; doi:10.1002/jcp.24402)

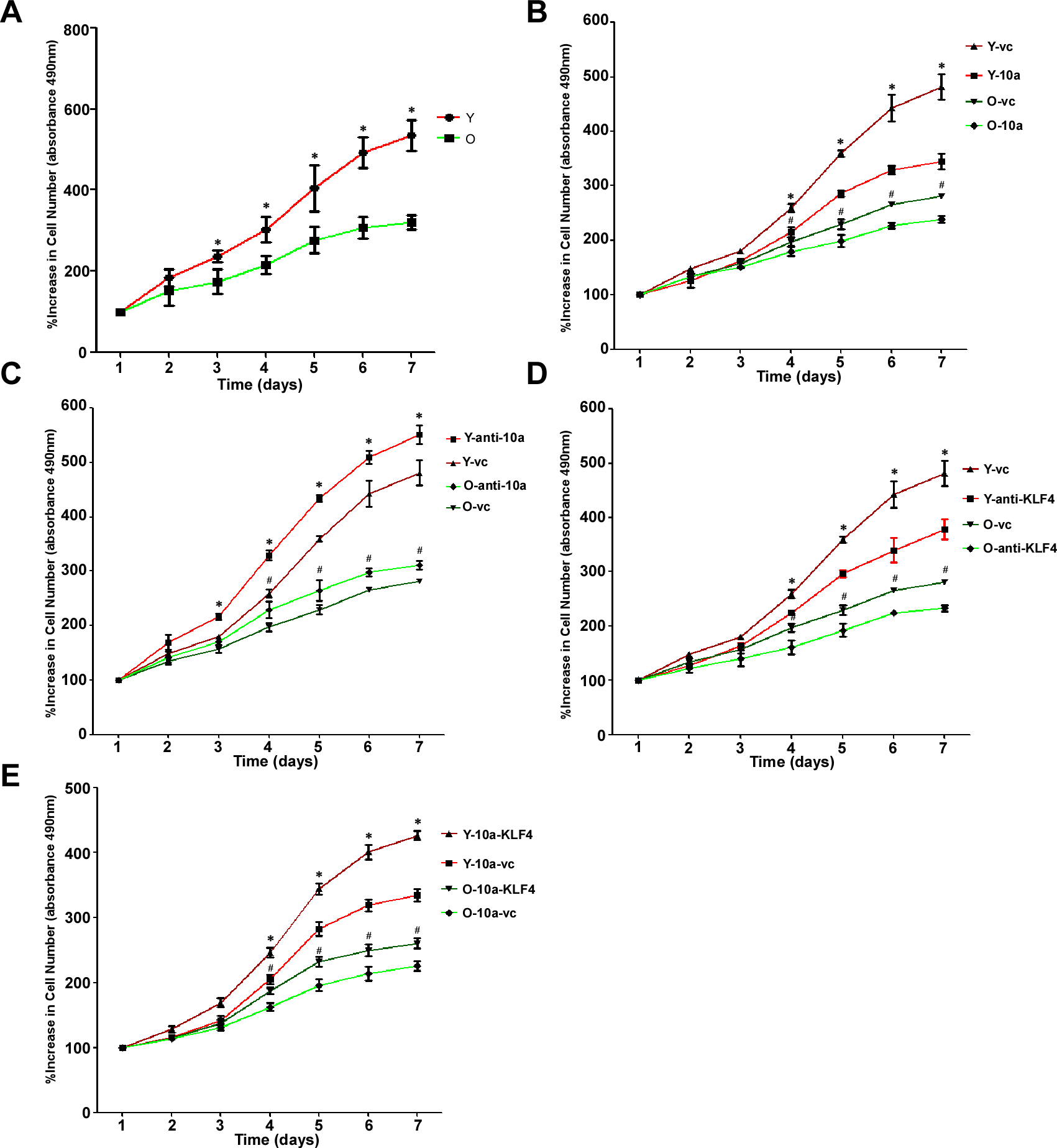

Supplement: Supplementary file 1 — Fig. S1. In vitro proliferation. Seven-day growth curves are presented for young (Y) and old (O) hMSCs. A: Non-transduced hMSCs. B: LV-miR-10a-transduced hMSCs. C: LV-anti-10a-transduced hMSCs. D: LV-anti-KLF4-transduced hMSCs. E: LV-KLF4 and miR-10a cotransduced hMSCs. The data represent mean ± SD (n = 4/group). *P < 0.05 Y versus O hMSCs; Y-10a, Y-anti-10a or Y-anti-KLF4 versus Y-vc; Y-10a-KLF4 versus Y-10a-vc. #P < 0.05 O-10a, O-anti-10a or O-anti-KLF4 versus O-vc; O-10a-KLF4 versus O-10a-vc. [file jcp0228-2324-sd1.tif]

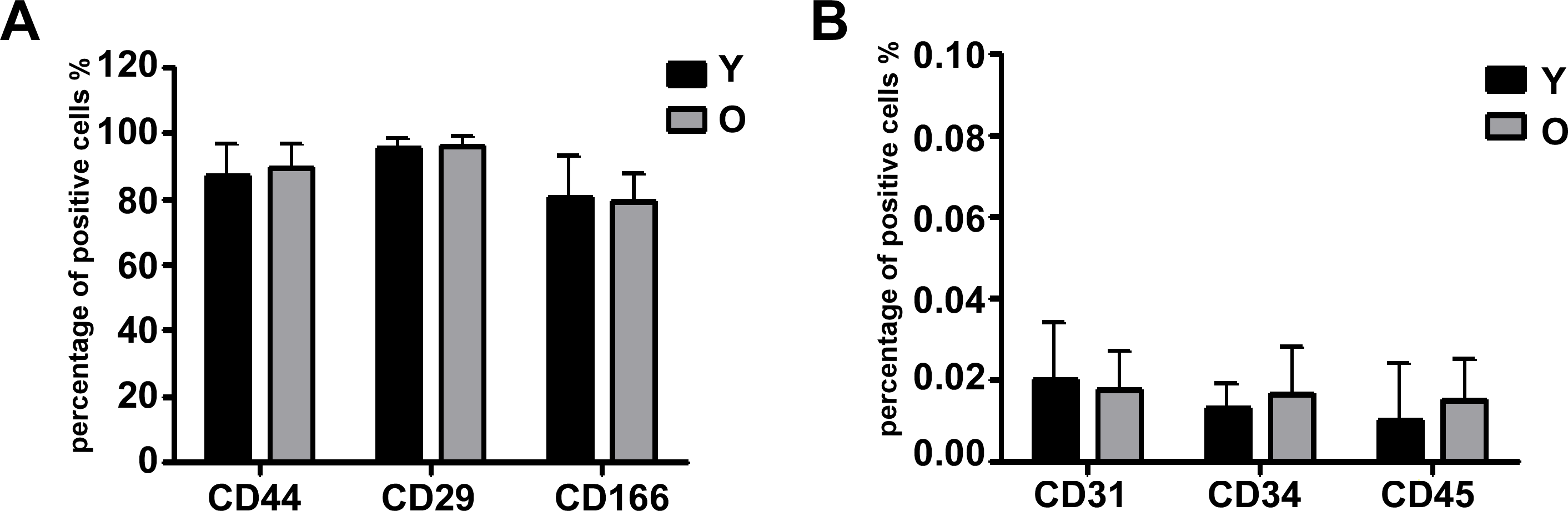

Supplement: Supplementary file 2 — Fig. S2. hMSC surface antigen expression. Flow cytometric analysis of cell surface antigens shows that hMSCs are positive for CD44, CD29, and CD166 (A) and negative for CD31, CD34, and CD45 (B) in both young (Y) and old (O) hMSCs. The data represent mean ± SD (n = 4/group). [file jcp0228-2324-sd2.tif]

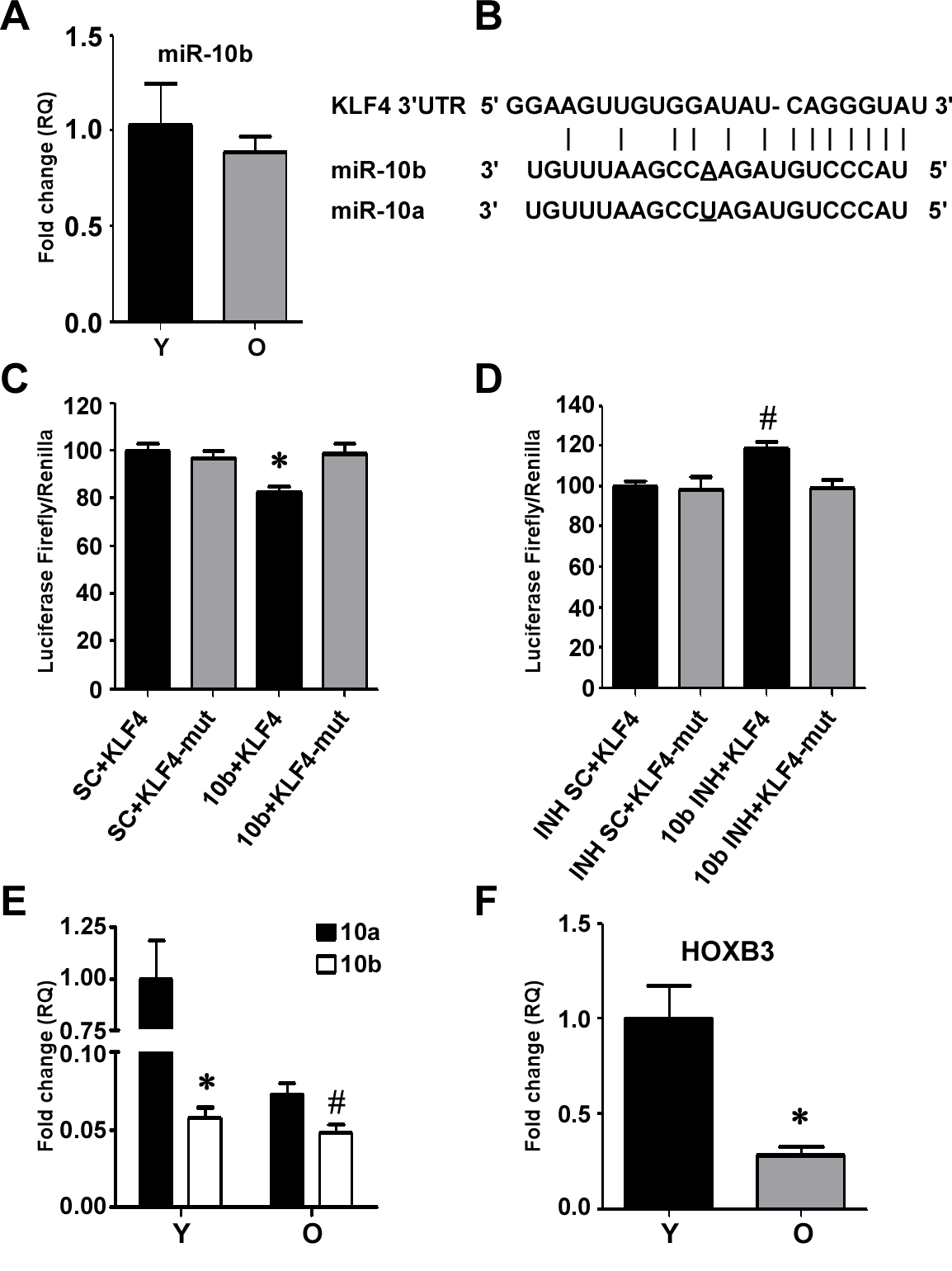

Supplement: Supplementary file 3 — Fig. S3. Relative expression of miR-10a and miR-10b. A: miR-10b expression in young (Y) and old (O) hMSCs. B: miR-10b binding site at the 3′-UTR of KLF4. C: hMSCs were cotransfected with a miR-10b mimic or a scrambled control and pGL4.13-KLF4-3′UTR or pGL4.13-KLF4-3′UTR-mut. Renilla and firefly luciferase activities were measured 48 h after transfection. The luciferase activity for KLF4 3′-UTR was normalized to endogenous renilla luciferase. D: hMSCs were cotransfected with a miR-10b inhibitor or a scrambled control and pGL4.13-KLF4-3′UTR or pGL4.13-KLF4-3′UTR-mut. Renilla and firefly luciferase activities were measured 48 h after transfection. The luciferase activity for KLF4 3′-UTR was normalized by the endogenous renilla luciferase. E: The basal expression levels of miR-10a and miR-10b in Y and O hMSCs. F: The basal expression levels of HOXB3 in Y and O hMSCs. The data represent mean ± SD (n = 3–4/group). *P < 0.05 10b + KLF4 versus SC + KLF4, Y-10a versus Y-10b, Y-HOXB3 versus O-HOXB3. #P < 0.05 10b INH + KLF4 versus INH SC + KLF4, O-10a versus O-10b. [file jcp0228-2324-sd3.tif]
