# Supplementary material for: miR-10a restores human mesenchymal stem cell differentiation by repressing KLF4
Source: J Cell Physiol. 2013 Aug 23;228(12):2324–36. doi: 10.1002/jcp.24402 (PMC4285942; doi:10.1002/jcp.24402)
Supplement: Supplementary file 4 — Table S1. Patient demographic information. [file jcp0228-2324-sd4.doc]

**Supplementary Table S1.** **Patient demographic information.**

| **Name** | **MiroRNA array** | | **Validation** | |
| --- | --- | --- | --- | --- |
|  | **younger** | **older** | **younger** | **older** |
| **n** | **3** | **3** | **30** | **30** |
| **age** | **17,20,25** | **75,78,80** | **17-30** | **65-80** |
| **sex** | **2M,1F** | **2M,1F** | **15M,15F** | **15M,15F** |

**Abbreviations: F, Female; M, Male.**
