# Supplementary material for: miR-10a restores human mesenchymal stem cell differentiation by repressing KLF4
Source: J Cell Physiol. 2013 Aug 23;228(12):2324–36. doi: 10.1002/jcp.24402 (PMC4285942; doi:10.1002/jcp.24402)
Supplement: Supplementary file 5 — Table S2. miRNA RT primer and qRT-PCR primer sequences. [file jcp0228-2324-sd5.doc]

**Supplementary Table S2. miRNA RT primer and qRT-PCR primer sequences.**

| **Name** | **Forward Primer** | | **Reverse Primer** |
| --- | --- | --- | --- |
| **Adipsin** | CCCTACATGGCGTCGGTGCA | | ATGGTGTCGGGCTGGCTGTC |
| **AP2** | GCATGGCCAAACCTAACATGA | | CCTGGCCCAGTATGAAGGAAA |
| **C/EBP-α** | CGCCTTCAACGACGAGTTCCTG | | CGCCTTGGCCTTCTCCTGCT |
| **PPARG** | GGCTTCATGACAAGGGAGTTTC | | AAACTCAAACTTGGGCTCCATAAA |
| **Runx2** | CTCTACTATGGCACTTCGTCA | | CTTCCATCAGCGTCAACAC |
| **ALP** | GCCAGGGCTGTAAGGACA | | CCAGATGAAGTGGGAGTGC |
| **PON** | ATGCTACAGACGAGGACA | | CTATCAATCACATCGGAAT |
| **OSTE** | AGGGCAGCGAGGTAGTGA | | CCTGAAAGCCGATGTGGT |
| **Sox9** | ACCACCAGAACTCCAGCTCCT | | TCTGCGGGATGGAAGGGA |
| **Aggrecan** | GGCATCGTGTTCCATTAC | | TCTCCATAGCAGCCTTCC |
| **Co12al** | GCTCCCAGAACATCACCTACC | | TGAACCTGCTATTGCCCTCT |
| **KLF4** | CCGCTCCATTACCAAGAGCT | | TGGTCAGTTCATCTGAGCGG |
| **HOXB3** | CTTTCCCATCACCCTTCCG | GGCGCTTCTTGGATTCTACC | |
| **GAPDH** | AACGGATTTGGTCGTATTG | GGAAGATGGTGATGGGATT | |
| **U6** | CTCGCTTCGGCAGCACA | | AACGCTTCACGAATTTGCGT |
| **miR-10a** | GGATACCCTGTAGATCCGAA | | CAGTGCGTGTCGTGGAGT |
| **miR-10b** | GGATACCCTGTAGAACCGAA | | CAGTGCGTGTCGTGGAGT |
| **miR-196a** | ACACTCCAGCTGGGTAGGTAGTTTCATGTTGTT | | CTCAACTGGTGTCGTGGA |
| **miR-486-5p** | ACACTCCAGCTGGGTCCTGTACTGAGCTGCCC | | CTCAACTGGTGTCGTGGA |
| **miR-664 star** | ACACTCCAGCTGGGACTGGCTAGGGAAAATGAT | | CTCAACTGGTGTCGTGGA |
| **miR-3197** | ACACTCCAGCTGGGGGAGGCGCAGGCTCGGAAA | | CTCAACTGGTGTCGTGGA |
| **KLF4-3’UTR** | TACACGCGTATCCCAGACAGTGGATATG | | TCTGTCGACGAGTATGCAAAATACAAACTCC |
| **KLF4-3’UTR-mut** | GATATCACCCAATAAATTATATCCGTGAG | | ACAACTTCCAGTCACCCCCTTG |
| **miR-10a RT primer** | GTCGTATCCAGTGCGTCGTGGAGTCGGCAATTGCACTGGATACGACCACAAA | | |
