# Supplementary material for: miR-10a restores human mesenchymal stem cell differentiation by repressing KLF4
Source: J Cell Physiol. 2013 Aug 23;228(12):2324–36. doi: 10.1002/jcp.24402 (PMC4285942; doi:10.1002/jcp.24402)
Supplement: Supplementary file 6 — Table S3. The sequences of the lentivirus and the miR-10a and miR-10b mimics and inhibitors. [file jcp0228-2324-sd6.doc]

**Supplementary Table S3. The sequences of the lentivirus and the miR-10a and miR-10b mimics and inhibitors.**

| **Name** | **Sequences** |
| --- | --- |
| **LV-control** | TTCTCCGAACGTGTCACGT |
| **LV-miR-10a** | TACCCTGTAGATCCGAATTTGTG |
| **LV-anti-10a** | CACAAATTTTCGGATCTACAGGGTA |
| **LV-anti-KLF4** | GCCACCCACACTTGTGATTAC |
| **miR-10a inhibitor scrambled** **control** | CAGUACUUUUGUGUAGUACAA |
| **miR-10a inhibitor** | CACAAAUUCGGAUCUACAGGGUA |
| **miR-10b inhibitor scrambled** **control** | CAGUACUUUUGUGUAGUACAA |
| **miR-10b inhibitor** | CACAAAUUCGGUUCUACAGGGUA |
| **miR-10a mimic scrambled** **control** | anti-sense ACGUGACACGUUCGGAGAATT |
|  | sense UUCUCCGAACGUGUCACGUTT |
| **miR-10a mimic** | anti-sense UACCCUGUAGAUCCGAAUUUGUG |
|  | sense CAAAUUCGGAUCUACAGGGUAUU |
| **miR-10b mimic scrambled** **control** | anti-sense UUCUCCGAACGUGUCACGUTT |
|  | sense ACGUGACACGUUCGGAGAATT |
| **miR-10b mimic** | anti-sense UACCCUGUAGAACCGAAUUUGUG |
|  | sense CAAAUUCGGUUCUACAGGGUAUU |
