# Supplementary material for: miR-10a restores human mesenchymal stem cell differentiation by repressing KLF4
Source: J Cell Physiol. 2013 Aug 23;228(12):2324–36. doi: 10.1002/jcp.24402 (PMC4285942; doi:10.1002/jcp.24402)
Supplement: Supplementary file 7 — Table S4. miRNA expression of hMSCs of different ages. [file jcp0228-2324-sd7.doc]

**Supplementary Table S4. miRNA expression of hMSCs of different ages.**.

| **ProbeSetID** | **H78** | **H80** | **H75** | **H17** | **H20** | **H25** |
| --- | --- | --- | --- | --- | --- | --- |
| hsa-let-7a_st | 4182.23 | 6813.64 | 8677.44 | 8545.44 | 6935.14 | 5939.12 |
| hsa-let-7a-star_st | 15.68 | 18.28 | 13.29 | 18.12 | 18.08 | 18.05 |
| hsa-let-7a-2-star_st | 22.82 | 43.07 | 22.11 | 47.46 | 26.68 | 26.53 |
| hsa-let-7b_st | 23694.99 | 24187.60 | 24276.83 | 23875.74 | 23460.63 | 23027.10 |
| hsa-let-7b-star_st | 23.11 | 26.63 | 17.30 | 24.06 | 24.16 | 21.85 |
| hsa-let-7c_st | 5914.33 | 7949.90 | 10977.22 | 9923.15 | 9020.90 | 7871.79 |
| hsa-let-7c-star_st | 18.00 | 15.69 | 18.21 | 17.60 | 14.73 | 17.26 |
| hsa-let-7d_st | 3632.90 | 4268.04 | 4784.67 | 4349.95 | 3632.61 | 4088.06 |
| hsa-let-7d-star_st | 17.29 | 24.97 | 18.97 | 27.42 | 17.72 | 27.30 |
| hsa-let-7e_st | 6328.07 | 8136.12 | 7113.50 | 6946.49 | 8383.02 | 8470.35 |
| hsa-let-7e-star_st | 22.41 | 27.87 | 29.21 | 32.01 | 31.64 | 31.41 |
| hsa-let-7f_st | 257.60 | 447.33 | 423.25 | 517.65 | 393.36 | 217.76 |
| hsa-let-7f-1-star_st | 24.10 | 16.95 | 16.26 | 18.48 | 23.46 | 25.21 |
| hsa-let-7f-2-star_st | 12.40 | 12.54 | 18.20 | 15.92 | 17.65 | 14.79 |
| hsa-miR-15a_st | 226.16 | 327.70 | 268.24 | 490.97 | 250.77 | 417.31 |
| hsa-miR-15a-star_st | 13.18 | 19.85 | 14.82 | 21.33 | 17.96 | 14.29 |
| hsa-miR-16_st | 6783.10 | 5611.56 | 5902.81 | 6648.03 | 6352.21 | 6435.38 |
| hsa-miR-16-1-star_st | 17.84 | 15.34 | 17.62 | 15.53 | 16.73 | 11.97 |
| hsa-miR-17_st | 4512.11 | 2091.69 | 1284.02 | 1428.27 | 2108.22 | 1926.70 |
| hsa-miR-17-star_st | 98.77 | 46.03 | 36.12 | 54.32 | 51.09 | 57.99 |
| hsa-miR-18a_st | 633.87 | 260.08 | 136.10 | 195.63 | 247.94 | 267.50 |
| hsa-miR-18a-star_st | 36.51 | 27.51 | 13.66 | 19.21 | 23.45 | 22.76 |
| hsa-miR-19a-star_st | 16.84 | 16.95 | 17.17 | 15.96 | 15.34 | 11.91 |
| hsa-miR-19a_st | 26.20 | 25.73 | 24.38 | 44.69 | 31.11 | 25.42 |
| hsa-miR-19b-1-star_st | 13.43 | 13.11 | 11.02 | 13.79 | 16.78 | 19.12 |
| hsa-miR-19b_st | 623.65 | 525.12 | 371.21 | 704.09 | 457.14 | 457.51 |
| hsa-miR-19b-2-star_st | 16.99 | 16.40 | 15.33 | 15.13 | 15.38 | 16.99 |
| hsa-miR-20a_st | 2703.53 | 1285.72 | 938.37 | 1009.89 | 1126.13 | 1104.34 |
| hsa-miR-20a-star_st | 12.52 | 14.94 | 18.11 | 13.36 | 17.97 | 15.00 |
| hsa-miR-21_st | 415.39 | 532.71 | 422.11 | 956.00 | 610.89 | 458.67 |
| hsa-miR-21-star_st | 172.44 | 117.34 | 48.21 | 105.45 | 108.51 | 202.53 |
| hsa-miR-22-star_st | 29.36 | 48.61 | 26.66 | 64.89 | 38.96 | 58.33 |
| hsa-miR-22_st | 10160.03 | 9799.46 | 10505.06 | 11390.87 | 10754.40 | 10765.79 |
| hsa-miR-23a-star_st | 182.91 | 240.94 | 105.54 | 64.90 | 117.51 | 138.09 |
| hsa-miR-23a_st | 13348.75 | 16472.71 | 16901.77 | 16087.08 | 15400.01 | 14884.09 |
| hsa-miR-24-1-star_st | 19.23 | 22.71 | 15.48 | 19.46 | 17.02 | 19.01 |
| hsa-miR-24_st | 21451.23 | 21367.58 | 21550.17 | 20676.52 | 19424.12 | 20023.01 |
| hsa-miR-24-2-star_st | 262.78 | 477.20 | 316.50 | 293.24 | 278.28 | 312.27 |
| hsa-miR-25-star_st | 115.39 | 36.55 | 37.53 | 43.86 | 36.74 | 35.15 |
| hsa-miR-25_st | 187.58 | 272.39 | 267.29 | 287.32 | 166.70 | 191.26 |
| hsa-miR-26a_st | 7209.00 | 8440.95 | 8255.64 | 8164.69 | 8502.11 | 7642.82 |
| hsa-miR-26a-1-star_st | 12.75 | 13.77 | 16.56 | 13.41 | 14.09 | 16.16 |
| hsa-miR-26b_st | 22.68 | 28.71 | 29.83 | 35.05 | 17.21 | 25.42 |
| hsa-miR-26b-star_st | 15.04 | 21.13 | 13.10 | 15.37 | 17.11 | 14.68 |
| hsa-miR-27a-star_st | 175.60 | 200.40 | 65.06 | 72.51 | 100.13 | 103.39 |
| hsa-miR-27a_st | 5175.49 | 7761.71 | 6083.85 | 8014.44 | 7170.11 | 8833.65 |
| hsa-miR-28-5p_st | 647.99 | 977.60 | 608.05 | 631.76 | 813.43 | 857.57 |
| hsa-miR-28-3p_st | 296.00 | 511.52 | 344.24 | 356.32 | 480.71 | 413.69 |
| hsa-miR-29a-star_st | 12.93 | 13.59 | 15.78 | 15.81 | 14.67 | 14.84 |
| hsa-miR-29a_st | 1003.77 | 1350.32 | 1429.58 | 1965.42 | 435.26 | 754.41 |
| hsa-miR-30a_st | 675.81 | 788.48 | 627.44 | 600.18 | 573.88 | 570.73 |
| hsa-miR-30a-star_st | 42.89 | 128.27 | 150.05 | 94.09 | 79.73 | 102.44 |
| hsa-miR-31_st | 3958.62 | 5233.41 | 2391.12 | 6796.97 | 4085.18 | 6444.37 |
| hsa-miR-31-star_st | 26.98 | 33.63 | 32.75 | 129.83 | 23.71 | 49.65 |
| hsa-miR-32_st | 18.42 | 15.32 | 17.46 | 17.17 | 13.59 | 20.21 |
| hsa-miR-32-star_st | 14.99 | 15.25 | 17.17 | 12.24 | 19.16 | 16.50 |
| hsa-miR-33a_st | 15.84 | 11.95 | 11.69 | 13.18 | 15.88 | 18.00 |
| hsa-miR-33a-star_st | 16.98 | 12.65 | 12.42 | 15.18 | 17.61 | 18.85 |
| hsa-miR-92a-1-star_st | 98.24 | 22.93 | 21.46 | 26.74 | 17.01 | 17.56 |
| hsa-miR-92a_st | 3774.31 | 2280.74 | 1814.52 | 1895.68 | 2605.08 | 2077.53 |
| hsa-miR-92a-2-star_st | 11.03 | 13.75 | 10.45 | 11.58 | 14.35 | 12.36 |
| hsa-miR-93_st | 2061.78 | 2137.18 | 1390.84 | 1330.75 | 1858.62 | 1937.77 |
| hsa-miR-93-star_st | 111.83 | 164.94 | 96.67 | 102.35 | 149.56 | 136.83 |
| hsa-miR-95_st | 14.79 | 14.75 | 15.83 | 15.37 | 12.46 | 14.20 |
| hsa-miR-96_st | 11.64 | 15.90 | 15.74 | 14.57 | 14.37 | 11.57 |
| hsa-miR-96-star_st | 14.45 | 14.12 | 15.95 | 17.69 | 17.68 | 11.67 |
| hsa-miR-98_st | 64.15 | 95.19 | 80.31 | 72.64 | 77.49 | 79.96 |
| hsa-miR-99a_st | 427.52 | 693.01 | 969.84 | 542.39 | 658.34 | 584.01 |
| hsa-miR-99a-star_st | 15.05 | 9.72 | 13.56 | 17.79 | 10.70 | 14.02 |
| hsa-miR-100_st | 8030.68 | 11799.79 | 8199.01 | 8443.69 | 5486.80 | 8997.55 |
| hsa-miR-100-star_st | 16.28 | 15.48 | 14.62 | 16.90 | 14.61 | 16.18 |
| hsa-miR-101-star_st | 9.09 | 13.89 | 17.66 | 16.97 | 16.01 | 15.00 |
| hsa-miR-101_st | 13.74 | 18.63 | 15.33 | 13.62 | 16.84 | 15.54 |
| hsa-miR-29b-1-star_st | 82.39 | 129.16 | 86.74 | 75.00 | 38.92 | 60.60 |
| hsa-miR-29b_st | 33.96 | 19.00 | 22.79 | 32.63 | 15.12 | 12.59 |
| hsa-miR-29b-2-star_st | 29.09 | 27.53 | 39.05 | 24.44 | 26.12 | 25.58 |
| hsa-miR-103-2-star_st | 13.79 | 16.36 | 20.43 | 22.16 | 16.79 | 19.01 |
| hsa-miR-103_st | 12443.25 | 9119.38 | 7832.38 | 7264.16 | 10288.66 | 10270.06 |
| hsa-miR-105_st | 18.58 | 10.66 | 13.92 | 13.59 | 17.18 | 16.91 |
| hsa-miR-105-star_st | 18.04 | 17.26 | 17.86 | 18.05 | 17.86 | 18.06 |
| hsa-miR-106a_st | 3245.86 | 1668.26 | 1110.03 | 1191.84 | 1619.23 | 1475.21 |
| hsa-miR-106a-star_st | 18.73 | 14.97 | 14.61 | 16.90 | 13.13 | 15.00 |
| hsa-miR-107_st | 7161.10 | 6424.96 | 6165.00 | 5642.80 | 7716.41 | 6769.74 |
| hsa-miR-16-2-star_st | 20.58 | 19.88 | 17.65 | 19.10 | 17.62 | 25.82 |
| hsa-miR-192_st | 21.31 | 37.58 | 35.61 | 41.38 | 47.19 | 33.76 |
| hsa-miR-192-star_st | 14.20 | 14.57 | 16.89 | 16.67 | 18.89 | 13.10 |
| hsa-miR-196a_st | 45.68 | 178.44 | 270.12 | 25.44 | 18.71 | 16.56 |
| hsa-miR-197_st | 167.41 | 262.31 | 178.50 | 153.91 | 273.67 | 212.82 |
| hsa-miR-198_st | 14.96 | 16.09 | 14.79 | 13.89 | 15.95 | 15.00 |
| hsa-miR-199a-5p_st | 1298.93 | 2885.53 | 2684.28 | 3742.47 | 2715.45 | 2799.39 |
| hsa-miR-199a-3p_st | 1078.22 | 2140.50 | 2798.37 | 3047.34 | 1460.17 | 1468.23 |
| hsa-miR-208a_st | 18.56 | 16.90 | 14.69 | 16.46 | 21.93 | 18.86 |
| hsa-miR-129-5p_st | 23.90 | 22.61 | 22.17 | 30.76 | 23.94 | 15.99 |
| hsa-miR-129-star_st | 16.00 | 17.30 | 20.80 | 18.66 | 15.30 | 18.65 |
| hsa-miR-148a-star_st | 12.98 | 20.64 | 22.14 | 22.20 | 29.27 | 13.19 |
| hsa-miR-148a_st | 41.79 | 48.65 | 62.90 | 59.65 | 42.84 | 45.99 |
| hsa-miR-30c_st | 267.78 | 544.56 | 714.50 | 651.66 | 501.43 | 423.12 |
| hsa-miR-30c-2-star_st | 35.10 | 31.09 | 36.51 | 46.40 | 40.74 | 38.34 |
| hsa-miR-30d_st | 205.62 | 306.17 | 205.31 | 238.22 | 185.31 | 198.24 |
| hsa-miR-30d-star_st | 11.65 | 16.83 | 16.49 | 12.28 | 14.93 | 17.22 |
| hsa-miR-139-5p_st | 14.64 | 14.52 | 14.41 | 16.09 | 25.29 | 17.28 |
| hsa-miR-139-3p_st | 9.62 | 10.36 | 9.84 | 18.61 | 15.87 | 12.89 |
| hsa-miR-147_st | 13.73 | 12.87 | 15.03 | 15.19 | 13.23 | 15.48 |
| hsa-miR-7_st | 16.83 | 13.63 | 13.76 | 17.36 | 15.15 | 11.99 |
| hsa-miR-7-1-star_st | 19.61 | 12.88 | 18.11 | 24.79 | 20.12 | 15.99 |
| hsa-miR-7-2-star_st | 21.78 | 20.22 | 18.43 | 16.94 | 17.80 | 15.18 |
| hsa-miR-10a_st | 36.62 | 49.74 | 45.76 | 222.40 | 165.42 | 130.90 |
| hsa-miR-10a-star_st | 16.48 | 16.46 | 15.86 | 23.27 | 16.53 | 16.39 |
| hsa-miR-10b_st | 58.05 | 126.72 | 115.44 | 105.45 | 51.85 | 82.64 |
| hsa-miR-10b-star_st | 24.87 | 21.37 | 18.11 | 25.14 | 13.47 | 19.05 |
| hsa-miR-34a_st | 1858.52 | 1853.86 | 1941.96 | 1657.36 | 2042.28 | 1823.48 |
| hsa-miR-34a-star_st | 82.39 | 70.75 | 76.56 | 81.66 | 78.09 | 73.75 |
| hsa-miR-181a_st | 3250.00 | 3756.04 | 2845.52 | 3580.63 | 3458.48 | 3685.36 |
| hsa-miR-181a-2-star_st | 73.90 | 95.01 | 71.36 | 149.32 | 129.22 | 214.39 |
| hsa-miR-181b_st | 1215.45 | 1322.75 | 857.34 | 1153.51 | 1303.29 | 1351.33 |
| hsa-miR-181c_st | 81.81 | 98.81 | 79.58 | 89.82 | 75.77 | 45.85 |
| hsa-miR-181c-star_st | 47.41 | 31.82 | 27.48 | 37.92 | 24.94 | 33.23 |
| hsa-miR-182_st | 44.50 | 19.64 | 18.48 | 14.16 | 19.35 | 17.43 |
| hsa-miR-182-star_st | 22.41 | 15.33 | 20.79 | 20.50 | 17.63 | 19.90 |
| hsa-miR-183_st | 14.29 | 14.53 | 15.81 | 12.25 | 13.85 | 11.71 |
| hsa-miR-183-star_st | 18.28 | 20.45 | 18.35 | 16.66 | 21.63 | 15.83 |
| hsa-miR-187-star_st | 23.46 | 15.71 | 35.70 | 27.24 | 18.15 | 16.99 |
| hsa-miR-187_st | 18.68 | 28.89 | 25.02 | 27.85 | 18.32 | 17.65 |
| hsa-miR-196a-star_st | 15.53 | 17.35 | 12.46 | 13.79 | 13.87 | 16.33 |
| hsa-miR-199b-5p_st | 16.62 | 11.92 | 19.19 | 21.16 | 18.29 | 15.00 |
| hsa-miR-199b-3p_st | 1080.91 | 2292.60 | 2783.68 | 3040.25 | 1481.65 | 1557.58 |
| hsa-miR-203_st | 17.06 | 9.41 | 16.78 | 15.96 | 14.35 | 14.26 |
| hsa-miR-204_st | 17.87 | 19.12 | 17.96 | 16.93 | 16.92 | 15.49 |
| hsa-miR-205_st | 15.90 | 18.40 | 15.83 | 13.29 | 15.95 | 16.03 |
| hsa-miR-205-star_st | 18.87 | 12.96 | 16.07 | 14.21 | 15.09 | 11.25 |
| hsa-miR-210_st | 1631.12 | 1481.89 | 437.31 | 1763.46 | 2268.92 | 3240.01 |
| hsa-miR-211_st | 16.78 | 22.68 | 16.80 | 16.22 | 16.05 | 14.56 |
| hsa-miR-212_st | 126.52 | 157.43 | 63.77 | 78.64 | 126.82 | 135.30 |
| hsa-miR-181a-star_st | 38.97 | 60.63 | 44.22 | 60.96 | 53.85 | 64.39 |
| hsa-miR-214-star_st | 78.93 | 120.33 | 88.04 | 111.02 | 109.81 | 116.43 |
| hsa-miR-214_st | 3893.35 | 4444.00 | 4129.00 | 2648.29 | 4893.69 | 4160.52 |
| hsa-miR-215_st | 19.19 | 10.58 | 15.14 | 18.88 | 13.10 | 14.64 |
| hsa-miR-216a_st | 16.98 | 19.40 | 18.14 | 19.67 | 18.18 | 18.60 |
| hsa-miR-217_st | 26.85 | 23.74 | 20.60 | 21.70 | 21.81 | 18.50 |
| hsa-miR-218_st | 16.22 | 14.25 | 11.61 | 15.55 | 16.51 | 18.00 |
| hsa-miR-218-1-star_st | 15.37 | 17.66 | 14.41 | 15.57 | 18.64 | 15.40 |
| hsa-miR-218-2-star_st | 16.26 | 11.00 | 17.89 | 16.19 | 16.14 | 18.00 |
| hsa-miR-219-5p_st | 15.83 | 13.02 | 15.15 | 14.35 | 17.14 | 14.70 |
| hsa-miR-219-1-3p_st | 10.19 | 12.27 | 17.06 | 12.61 | 17.42 | 15.00 |
| hsa-miR-220a_st | 12.78 | 18.51 | 17.12 | 13.59 | 14.18 | 19.70 |
| hsa-miR-221-star_st | 39.17 | 90.57 | 76.46 | 86.26 | 71.90 | 61.55 |
| hsa-miR-221_st | 8870.08 | 12897.91 | 12383.56 | 13133.36 | 13954.56 | 15450.82 |
| hsa-miR-222-star_st | 22.87 | 49.34 | 16.74 | 32.66 | 18.73 | 31.58 |
| hsa-miR-222_st | 17956.57 | 14345.70 | 13189.68 | 12648.57 | 14551.83 | 12965.67 |
| hsa-miR-223-star_st | 13.79 | 18.51 | 17.26 | 16.42 | 16.50 | 11.27 |
| hsa-miR-223_st | 20.99 | 16.62 | 20.93 | 18.74 | 13.20 | 18.46 |
| hsa-miR-224_st | 24.91 | 74.93 | 35.40 | 88.52 | 47.10 | 36.59 |
| hsa-miR-224-star_st | 107.22 | 336.81 | 154.44 | 244.12 | 218.39 | 242.78 |
| hsa-miR-200b-star_st | 13.56 | 23.92 | 15.51 | 18.23 | 12.13 | 21.08 |
| hsa-miR-200b_st | 16.89 | 14.85 | 15.33 | 18.64 | 12.01 | 21.08 |
| hsa-let-7g_st | 148.30 | 312.12 | 365.82 | 373.20 | 289.04 | 226.42 |
| hsa-let-7g-star_st | 22.75 | 12.38 | 14.41 | 20.89 | 18.08 | 17.14 |
| hsa-let-7i_st | 2434.18 | 4366.55 | 5516.94 | 5850.17 | 3895.38 | 3028.61 |
| hsa-let-7i-star_st | 51.05 | 53.44 | 55.49 | 61.94 | 55.63 | 62.24 |
| hsa-miR-1_st | 18.02 | 14.17 | 14.40 | 14.41 | 17.10 | 15.65 |
| hsa-miR-15b_st | 242.65 | 310.72 | 404.98 | 369.58 | 248.38 | 285.64 |
| hsa-miR-15b-star_st | 15.83 | 14.83 | 12.60 | 14.72 | 13.86 | 15.00 |
| hsa-miR-23b-star_st | 38.01 | 47.73 | 32.72 | 30.96 | 48.91 | 64.40 |
| hsa-miR-23b_st | 10677.95 | 10728.28 | 13590.82 | 12152.75 | 12692.66 | 12241.74 |
| hsa-miR-27b-star_st | 99.76 | 121.14 | 116.83 | 112.73 | 108.45 | 124.98 |
| hsa-miR-27b_st | 976.81 | 1900.73 | 2547.45 | 2831.03 | 1943.88 | 2256.32 |
| hsa-miR-30b_st | 105.01 | 150.29 | 143.79 | 229.11 | 136.50 | 110.57 |
| hsa-miR-30b-star_st | 28.62 | 30.71 | 30.87 | 26.34 | 28.39 | 39.03 |
| hsa-miR-122_st | 17.73 | 19.71 | 18.70 | 19.64 | 17.32 | 21.88 |
| hsa-miR-122-star_st | 17.53 | 15.55 | 15.17 | 15.51 | 16.36 | 16.52 |
| hsa-miR-124-star_st | 20.92 | 11.56 | 14.31 | 11.16 | 16.80 | 15.00 |
| hsa-miR-124_st | 38.02 | 49.10 | 32.63 | 30.67 | 24.29 | 28.30 |
| hsa-miR-125b_st | 14289.23 | 14880.75 | 14590.89 | 15066.51 | 13981.40 | 13312.83 |
| hsa-miR-125b-1-star_st | 294.80 | 418.43 | 173.13 | 207.86 | 280.26 | 377.69 |
| hsa-miR-128_st | 46.25 | 76.01 | 75.11 | 84.05 | 43.89 | 54.81 |
| hsa-miR-130a-star_st | 11.71 | 14.08 | 13.08 | 13.78 | 14.89 | 15.61 |
| hsa-miR-130a_st | 584.14 | 666.50 | 661.00 | 860.62 | 764.31 | 792.15 |
| hsa-miR-132-star_st | 14.29 | 15.70 | 16.28 | 23.87 | 19.15 | 20.04 |
| hsa-miR-132_st | 652.01 | 995.14 | 533.42 | 683.55 | 1091.06 | 980.19 |
| hsa-miR-133a_st | 28.25 | 19.84 | 13.41 | 15.83 | 17.78 | 14.08 |
| hsa-miR-135a_st | 14.03 | 16.15 | 14.99 | 12.74 | 17.32 | 13.84 |
| hsa-miR-135a-star_st | 20.48 | 17.70 | 25.49 | 27.14 | 21.63 | 20.04 |
| hsa-miR-137_st | 18.09 | 19.25 | 18.29 | 27.20 | 18.32 | 22.15 |
| hsa-miR-138_st | 788.62 | 3557.57 | 1818.33 | 2737.62 | 4825.00 | 3814.65 |
| hsa-miR-138-2-star_st | 13.46 | 14.70 | 12.60 | 14.10 | 16.43 | 15.59 |
| hsa-miR-140-5p_st | 198.03 | 246.14 | 246.97 | 272.20 | 301.43 | 246.04 |
| hsa-miR-140-3p_st | 4749.67 | 3873.52 | 3249.58 | 2308.00 | 4787.34 | 4684.98 |
| hsa-miR-141-star_st | 13.34 | 14.86 | 16.43 | 16.03 | 13.86 | 15.16 |
| hsa-miR-141_st | 13.67 | 15.50 | 12.73 | 15.22 | 15.79 | 13.34 |
| hsa-miR-142-5p_st | 15.71 | 17.15 | 13.67 | 11.65 | 18.30 | 23.12 |
| hsa-miR-142-3p_st | 15.44 | 17.26 | 12.42 | 14.38 | 13.06 | 17.03 |
| hsa-miR-143-star_st | 406.62 | 435.83 | 224.43 | 278.28 | 573.02 | 606.28 |
| hsa-miR-143_st | 12647.09 | 13736.23 | 12700.01 | 14015.45 | 16724.64 | 16782.12 |
| hsa-miR-144-star_st | 19.30 | 16.36 | 15.47 | 15.25 | 14.45 | 15.65 |
| hsa-miR-144_st | 19.36 | 16.87 | 13.43 | 14.11 | 13.86 | 13.65 |
| hsa-miR-145_st | 16455.83 | 19280.88 | 18189.84 | 21517.88 | 21077.95 | 21465.11 |
| hsa-miR-145-star_st | 25.31 | 25.13 | 20.14 | 31.26 | 29.96 | 26.74 |
| hsa-miR-152_st | 1172.62 | 2619.96 | 2529.21 | 2141.30 | 1439.00 | 1656.93 |
| hsa-miR-153_st | 15.56 | 14.78 | 18.16 | 18.92 | 18.00 | 18.01 |
| hsa-miR-191_st | 5768.85 | 5080.21 | 5494.53 | 3872.73 | 4641.68 | 4850.70 |
| hsa-miR-191-star_st | 27.91 | 20.55 | 30.03 | 24.72 | 19.16 | 18.00 |
| hsa-miR-9_st | 18.34 | 15.14 | 16.37 | 16.64 | 14.05 | 15.77 |
| hsa-miR-9-star_st | 17.13 | 17.12 | 19.08 | 14.10 | 15.09 | 21.55 |
| hsa-miR-125a-5p_st | 5180.64 | 6794.57 | 6260.02 | 7037.50 | 6467.81 | 6584.17 |
| hsa-miR-125a-3p_st | 138.81 | 147.60 | 74.71 | 83.99 | 145.58 | 127.46 |
| hsa-miR-125b-2-star_st | 36.69 | 29.74 | 58.97 | 40.04 | 42.40 | 56.21 |
| hsa-miR-126-star_st | 11.58 | 11.56 | 16.04 | 12.46 | 11.76 | 12.01 |
| hsa-miR-126_st | 32.40 | 27.67 | 22.46 | 46.57 | 39.32 | 43.45 |
| hsa-miR-127-5p_st | 73.10 | 88.26 | 86.32 | 108.50 | 111.34 | 106.26 |
| hsa-miR-127-3p_st | 2920.44 | 2840.01 | 3769.14 | 3617.82 | 3943.28 | 3836.28 |
| hsa-miR-129-3p_st | 24.08 | 16.37 | 19.48 | 26.38 | 19.92 | 20.27 |
| hsa-miR-134_st | 513.38 | 600.80 | 631.60 | 414.41 | 622.63 | 722.52 |
| hsa-miR-136_st | 14.90 | 15.64 | 11.61 | 13.85 | 13.00 | 16.46 |
| hsa-miR-136-star_st | 13.81 | 14.66 | 14.05 | 16.56 | 12.51 | 11.70 |
| hsa-miR-138-1-star_st | 139.59 | 317.60 | 144.05 | 160.01 | 295.05 | 281.46 |
| hsa-miR-146a_st | 770.01 | 17.06 | 127.04 | 113.79 | 14.89 | 16.99 |
| hsa-miR-146a-star_st | 16.45 | 13.72 | 16.25 | 11.49 | 16.42 | 14.67 |
| hsa-miR-149_st | 71.31 | 239.61 | 154.92 | 108.61 | 299.48 | 268.28 |
| hsa-miR-149-star_st | 972.45 | 780.02 | 2242.46 | 1971.26 | 1324.14 | 1182.16 |
| hsa-miR-150_st | 33.85 | 21.10 | 25.06 | 17.79 | 13.77 | 18.78 |
| hsa-miR-150-star_st | 31.41 | 29.77 | 55.94 | 45.44 | 48.43 | 29.07 |
| hsa-miR-154_st | 55.60 | 51.16 | 93.57 | 120.65 | 69.50 | 44.59 |
| hsa-miR-154-star_st | 27.64 | 18.88 | 20.27 | 47.09 | 18.19 | 29.06 |
| hsa-miR-184_st | 13.85 | 12.81 | 17.55 | 15.05 | 15.95 | 13.54 |
| hsa-miR-185_st | 1589.11 | 1955.15 | 1535.40 | 1086.45 | 1650.63 | 1605.89 |
| hsa-miR-185-star_st | 9.70 | 19.76 | 21.12 | 17.67 | 20.61 | 19.36 |
| hsa-miR-186_st | 17.64 | 16.94 | 12.70 | 17.71 | 14.47 | 17.86 |
| hsa-miR-186-star_st | 11.74 | 13.39 | 15.39 | 15.59 | 16.92 | 12.28 |
| hsa-miR-188-5p_st | 16.98 | 23.02 | 18.48 | 27.02 | 19.25 | 32.08 |
| hsa-miR-188-3p_st | 15.28 | 20.84 | 15.78 | 14.51 | 16.37 | 13.23 |
| hsa-miR-190_st | 18.88 | 15.61 | 17.63 | 13.18 | 15.46 | 16.86 |
| hsa-miR-193a-5p_st | 886.52 | 1100.86 | 947.13 | 662.96 | 1061.57 | 1324.15 |
| hsa-miR-193a-3p_st | 65.00 | 79.32 | 51.57 | 92.87 | 60.67 | 86.05 |
| hsa-miR-194_st | 86.98 | 102.26 | 75.20 | 99.26 | 97.63 | 122.82 |
| hsa-miR-195_st | 244.65 | 465.46 | 750.69 | 482.47 | 345.52 | 484.11 |
| hsa-miR-195-star_st | 14.35 | 21.45 | 18.94 | 16.12 | 18.22 | 16.23 |
| hsa-miR-206_st | 14.36 | 15.52 | 16.30 | 13.64 | 14.76 | 15.00 |
| hsa-miR-320a_st | 8171.84 | 6019.00 | 4459.03 | 3724.93 | 7300.00 | 6649.34 |
| hsa-miR-200c-star_st | 12.16 | 16.77 | 11.73 | 11.76 | 10.92 | 12.27 |
| hsa-miR-200c_st | 31.68 | 29.12 | 22.88 | 27.80 | 24.77 | 23.59 |
| hsa-miR-155_st | 15940.80 | 374.85 | 2675.31 | 586.00 | 341.17 | 733.03 |
| hsa-miR-155-star_st | 19.11 | 17.50 | 17.61 | 18.28 | 17.82 | 18.26 |
| hsa-miR-194-star_st | 13.64 | 13.02 | 15.27 | 16.08 | 14.28 | 13.39 |
| hsa-miR-106b_st | 1296.43 | 1353.04 | 1103.85 | 1359.18 | 1103.56 | 1232.83 |
| hsa-miR-106b-star_st | 202.40 | 226.17 | 122.93 | 131.61 | 216.15 | 222.60 |
| hsa-miR-29c-star_st | 15.29 | 14.80 | 15.88 | 15.48 | 15.41 | 15.53 |
| hsa-miR-29c_st | 26.66 | 21.47 | 24.97 | 23.12 | 22.82 | 22.05 |
| hsa-miR-30c-1-star_st | 21.18 | 15.81 | 18.33 | 24.03 | 25.20 | 21.91 |
| hsa-miR-200a-star_st | 12.75 | 13.68 | 13.32 | 12.88 | 14.31 | 11.13 |
| hsa-miR-200a_st | 9.62 | 16.09 | 15.65 | 17.79 | 14.13 | 14.15 |
| hsa-miR-302a-star_st | 15.82 | 10.59 | 13.21 | 15.31 | 14.87 | 16.74 |
| hsa-miR-302a_st | 14.82 | 13.79 | 11.81 | 15.89 | 14.87 | 12.22 |
| hsa-miR-219-2-3p_st | 15.88 | 14.86 | 15.29 | 13.28 | 16.84 | 15.00 |
| hsa-miR-34b-star_st | 19.06 | 22.90 | 32.02 | 29.26 | 26.09 | 18.32 |
| hsa-miR-34b_st | 9.75 | 13.07 | 20.99 | 19.89 | 21.08 | 15.99 |
| hsa-miR-34c-5p_st | 54.03 | 85.39 | 97.81 | 65.51 | 133.54 | 124.25 |
| hsa-miR-34c-3p_st | 65.88 | 84.98 | 97.65 | 49.79 | 97.62 | 95.03 |
| hsa-miR-299-5p_st | 26.41 | 31.27 | 53.51 | 100.53 | 55.06 | 44.44 |
| hsa-miR-299-3p_st | 83.76 | 118.45 | 80.64 | 159.71 | 154.74 | 139.29 |
| hsa-miR-301a_st | 17.56 | 25.62 | 30.78 | 53.59 | 32.92 | 25.89 |
| hsa-miR-99b_st | 5082.54 | 6291.00 | 4421.56 | 4543.06 | 6126.96 | 5592.19 |
| hsa-miR-99b-star_st | 189.22 | 324.25 | 163.68 | 180.46 | 305.83 | 305.69 |
| hsa-miR-296-5p_st | 12.97 | 17.12 | 15.28 | 17.52 | 20.31 | 16.29 |
| hsa-miR-296-3p_st | 23.73 | 44.01 | 28.27 | 41.32 | 35.56 | 39.82 |
| hsa-miR-130b-star_st | 16.13 | 17.76 | 14.41 | 14.66 | 15.05 | 17.54 |
| hsa-miR-130b_st | 609.26 | 684.09 | 485.61 | 340.01 | 704.93 | 791.07 |
| hsa-miR-30e_st | 85.15 | 80.80 | 56.69 | 99.33 | 60.56 | 61.33 |
| hsa-miR-30e-star_st | 15.71 | 20.78 | 23.21 | 29.58 | 15.95 | 16.71 |
| hsa-miR-26a-2-star_st | 13.71 | 17.68 | 16.05 | 16.95 | 18.21 | 22.14 |
| hsa-miR-361-5p_st | 1131.73 | 1846.02 | 1782.52 | 1527.70 | 1951.26 | 2148.54 |
| hsa-miR-361-3p_st | 20.16 | 17.52 | 18.26 | 21.99 | 18.94 | 14.70 |
| hsa-miR-362-5p_st | 156.42 | 194.46 | 147.24 | 110.20 | 230.38 | 229.88 |
| hsa-miR-362-3p_st | 16.50 | 17.78 | 17.76 | 20.84 | 19.08 | 15.09 |
| hsa-miR-363-star_st | 18.40 | 22.56 | 14.97 | 16.58 | 14.33 | 23.58 |
| hsa-miR-363_st | 26.04 | 11.13 | 19.08 | 16.13 | 16.93 | 16.97 |
| hsa-miR-365_st | 18.20 | 14.67 | 15.45 | 18.52 | 12.28 | 19.79 |
| hsa-miR-365-star_st | 42.09 | 42.88 | 40.27 | 40.39 | 48.63 | 92.42 |
| hsa-miR-302b-star_st | 13.44 | 14.73 | 16.29 | 14.15 | 17.13 | 12.03 |
| hsa-miR-302b_st | 15.71 | 11.51 | 13.83 | 13.41 | 20.96 | 11.49 |
| hsa-miR-302c-star_st | 15.73 | 13.76 | 19.50 | 13.63 | 13.86 | 14.73 |
| hsa-miR-302c_st | 16.51 | 13.99 | 13.87 | 15.91 | 13.86 | 18.90 |
| hsa-miR-302d-star_st | 19.11 | 13.72 | 14.71 | 15.77 | 14.72 | 18.02 |
| hsa-miR-302d_st | 13.79 | 18.00 | 12.80 | 15.97 | 15.41 | 13.03 |
| hsa-miR-367-star_st | 20.19 | 15.75 | 16.57 | 14.13 | 12.83 | 15.44 |
| hsa-miR-367_st | 18.69 | 13.42 | 16.45 | 12.89 | 14.94 | 19.39 |
| hsa-miR-376c_st | 87.55 | 128.25 | 172.01 | 396.97 | 166.15 | 167.10 |
| hsa-miR-369-5p_st | 15.14 | 24.71 | 26.66 | 30.11 | 17.51 | 20.19 |
| hsa-miR-369-3p_st | 15.67 | 15.66 | 16.25 | 15.96 | 12.83 | 12.67 |
| hsa-miR-370_st | 179.87 | 195.88 | 159.05 | 171.96 | 223.71 | 223.97 |
| hsa-miR-371-5p_st | 17.35 | 15.35 | 20.86 | 15.96 | 12.34 | 17.12 |
| hsa-miR-371-3p_st | 13.79 | 17.08 | 15.26 | 15.78 | 12.84 | 19.39 |
| hsa-miR-372_st | 15.90 | 17.33 | 17.14 | 16.85 | 15.08 | 16.09 |
| hsa-miR-373-star_st | 17.34 | 15.24 | 25.28 | 17.38 | 15.26 | 19.41 |
| hsa-miR-373_st | 18.83 | 15.22 | 14.60 | 14.72 | 16.27 | 16.25 |
| hsa-miR-374a_st | 14.62 | 17.71 | 14.40 | 19.39 | 17.09 | 16.99 |
| hsa-miR-374a-star_st | 17.56 | 17.56 | 17.37 | 15.56 | 16.93 | 18.51 |
| hsa-miR-375_st | 15.71 | 13.69 | 14.07 | 13.78 | 10.22 | 16.52 |
| hsa-miR-376a-star_st | 16.96 | 11.82 | 10.76 | 12.24 | 11.48 | 14.94 |
| hsa-miR-376a_st | 19.84 | 32.38 | 37.68 | 81.24 | 31.16 | 44.23 |
| hsa-miR-377-star_st | 38.59 | 66.07 | 70.62 | 85.48 | 67.65 | 112.52 |
| hsa-miR-377_st | 24.94 | 24.48 | 22.32 | 37.62 | 19.36 | 20.92 |
| hsa-miR-378-star_st | 40.18 | 42.68 | 38.46 | 23.26 | 21.99 | 19.85 |
| hsa-miR-378_st | 601.88 | 271.61 | 299.52 | 125.83 | 182.39 | 158.98 |
| hsa-miR-379_st | 476.01 | 614.62 | 812.01 | 712.67 | 557.71 | 662.36 |
| hsa-miR-379-star_st | 13.94 | 13.83 | 21.68 | 17.70 | 17.18 | 18.66 |
| hsa-miR-380-star_st | 19.20 | 21.30 | 15.42 | 19.24 | 17.86 | 18.80 |
| hsa-miR-380_st | 15.29 | 13.26 | 18.18 | 14.36 | 16.61 | 15.42 |
| hsa-miR-381_st | 66.98 | 79.35 | 74.60 | 105.13 | 71.07 | 88.46 |
| hsa-miR-382_st | 344.32 | 487.53 | 689.06 | 607.55 | 599.91 | 560.97 |
| hsa-miR-383_st | 16.70 | 20.74 | 19.08 | 17.77 | 20.78 | 20.58 |
| hsa-miR-340_st | 14.71 | 17.86 | 17.16 | 17.42 | 15.92 | 12.18 |
| hsa-miR-340-star_st | 14.04 | 14.22 | 18.34 | 10.22 | 15.75 | 14.81 |
| hsa-miR-330-5p_st | 26.04 | 19.22 | 14.60 | 11.49 | 13.68 | 19.05 |
| hsa-miR-330-3p_st | 132.60 | 146.97 | 81.86 | 72.69 | 193.25 | 168.97 |
| hsa-miR-328_st | 35.52 | 49.04 | 42.36 | 45.37 | 60.64 | 36.63 |
| hsa-miR-342-5p_st | 58.46 | 33.93 | 54.81 | 40.50 | 46.51 | 36.87 |
| hsa-miR-342-3p_st | 1859.30 | 1361.00 | 1418.23 | 909.62 | 1418.42 | 1371.20 |
| hsa-miR-337-5p_st | 262.77 | 232.10 | 268.94 | 453.80 | 311.28 | 369.77 |
| hsa-miR-337-3p_st | 15.03 | 19.47 | 22.90 | 18.58 | 15.11 | 17.12 |
| hsa-miR-323-5p_st | 12.72 | 13.86 | 24.76 | 26.47 | 27.20 | 23.20 |
| hsa-miR-323-3p_st | 22.81 | 23.76 | 21.48 | 22.71 | 21.69 | 23.20 |
| hsa-miR-326_st | 15.81 | 15.54 | 11.83 | 16.60 | 14.07 | 14.19 |
| hsa-miR-151-5p_st | 1738.08 | 2303.26 | 2004.44 | 2297.83 | 2128.58 | 2562.15 |
| hsa-miR-151-3p_st | 344.93 | 599.42 | 472.72 | 425.91 | 424.08 | 538.63 |
| hsa-miR-135b_st | 17.90 | 16.19 | 19.14 | 16.92 | 17.42 | 14.89 |
| hsa-miR-135b-star_st | 15.50 | 14.38 | 19.54 | 15.88 | 17.99 | 18.03 |
| hsa-miR-148b-star_st | 22.11 | 16.35 | 20.85 | 17.14 | 14.89 | 15.99 |
| hsa-miR-148b_st | 32.81 | 34.63 | 51.60 | 60.72 | 32.68 | 20.12 |
| hsa-miR-331-5p_st | 23.00 | 22.30 | 22.11 | 24.52 | 31.96 | 31.35 |
| hsa-miR-331-3p_st | 65.04 | 61.22 | 53.22 | 84.94 | 102.88 | 81.75 |
| hsa-miR-324-5p_st | 413.30 | 387.65 | 246.82 | 250.82 | 481.20 | 468.85 |
| hsa-miR-324-3p_st | 180.64 | 192.90 | 135.12 | 110.51 | 195.20 | 175.32 |
| hsa-miR-338-5p_st | 14.65 | 14.99 | 12.07 | 14.36 | 14.04 | 18.23 |
| hsa-miR-338-3p_st | 15.96 | 11.92 | 13.57 | 14.12 | 14.12 | 19.58 |
| hsa-miR-339-5p_st | 342.32 | 259.12 | 215.42 | 186.16 | 453.64 | 450.31 |
| hsa-miR-339-3p_st | 196.48 | 169.06 | 138.32 | 119.07 | 233.88 | 249.36 |
| hsa-miR-335_st | 19.42 | 27.00 | 31.36 | 39.83 | 27.29 | 20.18 |
| hsa-miR-335-star_st | 19.30 | 18.28 | 16.52 | 18.36 | 14.41 | 18.53 |
| hsa-miR-133b_st | 14.51 | 20.80 | 15.14 | 19.72 | 18.36 | 21.00 |
| hsa-miR-325_st | 19.17 | 13.08 | 16.77 | 16.10 | 13.62 | 14.25 |
| hsa-miR-345_st | 176.05 | 214.19 | 93.97 | 157.28 | 205.19 | 214.30 |
| hsa-miR-346_st | 52.05 | 51.09 | 97.72 | 63.11 | 53.52 | 59.66 |
| hsa-miR-384_st | 17.96 | 14.38 | 15.24 | 13.35 | 15.88 | 14.94 |
| hsa-miR-196b_st | 28.42 | 73.78 | 63.77 | 45.31 | 18.25 | 24.32 |
| hsa-miR-196b-star_st | 18.16 | 24.05 | 26.66 | 23.97 | 23.46 | 30.08 |
| hsa-miR-422a_st | 73.09 | 41.04 | 84.03 | 46.21 | 32.39 | 21.59 |
| hsa-miR-423-5p_st | 522.06 | 507.32 | 287.87 | 203.59 | 496.36 | 417.35 |
| hsa-miR-423-3p_st | 722.87 | 915.90 | 647.51 | 492.71 | 849.68 | 804.45 |
| hsa-miR-424_st | 18.04 | 18.49 | 18.54 | 25.93 | 31.21 | 31.72 |
| hsa-miR-424-star_st | 119.00 | 238.84 | 195.40 | 166.53 | 556.46 | 676.53 |
| hsa-miR-425_st | 733.58 | 601.58 | 713.11 | 511.60 | 594.45 | 660.09 |
| hsa-miR-425-star_st | 74.91 | 89.11 | 94.28 | 60.44 | 79.06 | 97.78 |
| hsa-miR-18b_st | 47.68 | 19.10 | 30.76 | 34.20 | 25.53 | 26.61 |
| hsa-miR-18b-star_st | 15.15 | 18.51 | 12.53 | 15.20 | 16.64 | 11.91 |
| hsa-miR-20b_st | 400.60 | 86.52 | 92.31 | 114.45 | 77.62 | 68.89 |
| hsa-miR-20b-star_st | 12.22 | 19.31 | 13.13 | 15.37 | 18.68 | 16.24 |
| hsa-miR-448_st | 21.31 | 16.96 | 17.14 | 13.89 | 16.82 | 12.01 |
| hsa-miR-429_st | 16.71 | 15.77 | 18.94 | 17.01 | 15.85 | 19.06 |
| hsa-miR-449a_st | 14.12 | 15.61 | 15.20 | 14.86 | 12.49 | 16.37 |
| hsa-miR-450a_st | 16.81 | 11.70 | 16.98 | 13.46 | 15.95 | 15.99 |
| hsa-miR-431_st | 227.43 | 204.20 | 202.10 | 154.34 | 182.68 | 259.12 |
| hsa-miR-431-star_st | 36.22 | 31.26 | 40.02 | 40.78 | 40.07 | 35.35 |
| hsa-miR-433_st | 118.59 | 136.25 | 154.29 | 111.87 | 291.25 | 168.64 |
| hsa-miR-329_st | 14.84 | 22.90 | 32.83 | 72.64 | 48.43 | 26.33 |
| hsa-miR-451_st | 21.70 | 20.18 | 18.97 | 22.16 | 16.67 | 19.23 |
| hsa-miR-452_st | 25.03 | 79.76 | 43.52 | 89.40 | 45.12 | 40.34 |
| hsa-miR-452-star_st | 16.10 | 17.38 | 11.43 | 12.15 | 15.06 | 13.81 |
| hsa-miR-409-5p_st | 203.04 | 253.39 | 319.79 | 333.43 | 322.25 | 271.86 |
| hsa-miR-409-3p_st | 621.71 | 964.72 | 1279.41 | 1077.14 | 1040.66 | 1020.51 |
| hsa-miR-412_st | 14.11 | 17.58 | 13.57 | 16.90 | 12.03 | 15.02 |
| hsa-miR-410_st | 35.89 | 25.93 | 44.22 | 59.20 | 23.64 | 41.65 |
| hsa-miR-376b_st | 14.84 | 22.03 | 15.57 | 30.27 | 19.67 | 21.06 |
| hsa-miR-483-5p_st | 24.70 | 41.06 | 40.11 | 44.90 | 56.59 | 56.93 |
| hsa-miR-483-3p_st | 17.40 | 17.97 | 18.55 | 18.77 | 16.50 | 18.09 |
| hsa-miR-484_st | 29.18 | 29.50 | 28.70 | 29.74 | 26.51 | 35.11 |
| hsa-miR-485-5p_st | 114.90 | 96.17 | 127.88 | 106.83 | 159.81 | 139.29 |
| hsa-miR-485-3p_st | 25.53 | 44.53 | 37.08 | 56.11 | 53.84 | 44.84 |
| hsa-miR-486-5p_st | 129.66 | 207.80 | 115.49 | 85.84 | 39.48 | 37.00 |
| hsa-miR-486-3p_st | 68.99 | 69.97 | 36.98 | 45.49 | 25.10 | 16.67 |
| hsa-miR-487a_st | 126.10 | 121.24 | 108.01 | 141.31 | 111.78 | 160.93 |
| hsa-miR-488-star_st | 12.33 | 14.10 | 18.09 | 12.96 | 16.53 | 16.60 |
| hsa-miR-488_st | 17.50 | 13.83 | 14.06 | 18.78 | 13.32 | 14.19 |
| hsa-miR-489_st | 14.84 | 14.64 | 17.15 | 18.17 | 11.69 | 19.57 |
| hsa-miR-490-5p_st | 22.10 | 17.87 | 18.04 | 17.94 | 16.19 | 15.71 |
| hsa-miR-490-3p_st | 14.00 | 15.90 | 14.79 | 10.78 | 11.79 | 11.47 |
| hsa-miR-491-5p_st | 134.10 | 179.42 | 120.36 | 106.73 | 171.11 | 190.51 |
| hsa-miR-491-3p_st | 18.04 | 17.34 | 14.77 | 17.10 | 16.52 | 14.02 |
| hsa-miR-511_st | 17.54 | 18.70 | 14.58 | 14.93 | 14.89 | 11.45 |
| hsa-miR-146b-5p_st | 63.89 | 18.80 | 32.74 | 61.07 | 26.12 | 20.94 |
| hsa-miR-146b-3p_st | 15.83 | 12.65 | 10.39 | 10.57 | 13.59 | 14.93 |
| hsa-miR-202-star_st | 15.71 | 15.14 | 17.45 | 20.19 | 12.83 | 21.08 |
| hsa-miR-202_st | 17.94 | 19.01 | 18.94 | 17.72 | 16.42 | 16.27 |
| hsa-miR-492_st | 20.28 | 13.72 | 15.26 | 18.64 | 17.07 | 18.47 |
| hsa-miR-493-star_st | 19.69 | 25.93 | 36.95 | 52.80 | 28.17 | 27.06 |
| hsa-miR-493_st | 167.82 | 185.15 | 170.67 | 213.36 | 220.29 | 250.06 |
| hsa-miR-432_st | 548.48 | 751.27 | 1039.71 | 602.12 | 855.10 | 920.06 |
| hsa-miR-432-star_st | 15.10 | 19.65 | 18.32 | 16.02 | 19.64 | 20.10 |
| hsa-miR-494_st | 730.69 | 956.99 | 1490.53 | 1789.25 | 1179.52 | 1151.68 |
| hsa-miR-495_st | 56.53 | 56.52 | 69.38 | 99.22 | 39.06 | 82.74 |
| hsa-miR-496_st | 17.33 | 13.58 | 17.70 | 17.87 | 15.80 | 15.20 |
| hsa-miR-193b-star_st | 348.43 | 306.49 | 188.48 | 165.68 | 289.61 | 333.24 |
| hsa-miR-193b_st | 1737.32 | 1642.94 | 1446.06 | 1500.86 | 2370.36 | 2141.41 |
| hsa-miR-497_st | 120.43 | 114.56 | 167.59 | 160.23 | 104.54 | 187.84 |
| hsa-miR-497-star_st | 11.19 | 14.88 | 14.27 | 13.71 | 13.96 | 13.40 |
| hsa-miR-181d_st | 94.63 | 103.21 | 121.67 | 149.93 | 83.66 | 93.53 |
| hsa-miR-512-5p_st | 16.23 | 15.71 | 11.03 | 16.50 | 12.65 | 15.08 |
| hsa-miR-512-3p_st | 18.55 | 12.91 | 17.54 | 18.13 | 19.52 | 24.64 |
| hsa-miR-498_st | 29.60 | 22.39 | 35.01 | 35.50 | 28.72 | 20.54 |
| hsa-miR-520e_st | 15.49 | 9.64 | 11.22 | 13.74 | 14.56 | 12.85 |
| hsa-miR-515-5p_st | 14.84 | 14.41 | 15.76 | 15.34 | 14.89 | 14.17 |
| hsa-miR-515-3p_st | 10.45 | 13.23 | 9.70 | 16.11 | 13.09 | 13.59 |
| hsa-miR-519e-star_st | 15.72 | 17.35 | 14.25 | 19.23 | 18.03 | 16.99 |
| hsa-miR-519e_st | 12.41 | 13.21 | 14.22 | 13.55 | 11.59 | 12.44 |
| hsa-miR-520f_st | 19.49 | 17.89 | 16.25 | 17.52 | 18.01 | 11.57 |
| hsa-miR-519c-5p_st | 16.03 | 15.16 | 14.50 | 13.42 | 12.35 | 13.73 |
| hsa-miR-519c-3p_st | 14.01 | 15.26 | 17.17 | 15.41 | 12.83 | 19.30 |
| hsa-miR-520a-5p_st | 16.00 | 14.92 | 16.14 | 15.67 | 12.92 | 16.35 |
| hsa-miR-520a-3p_st | 13.66 | 13.82 | 16.62 | 14.75 | 16.81 | 13.32 |
| hsa-miR-526b_st | 14.65 | 14.92 | 13.34 | 12.90 | 17.93 | 14.87 |
| hsa-miR-526b-star_st | 14.79 | 13.72 | 16.44 | 15.02 | 14.09 | 16.38 |
| hsa-miR-519b-5p_st | 11.29 | 19.39 | 12.75 | 15.79 | 14.66 | 22.83 |
| hsa-miR-519b-3p_st | 12.71 | 15.50 | 13.07 | 11.31 | 17.76 | 23.78 |
| hsa-miR-525-5p_st | 14.95 | 16.24 | 12.98 | 15.62 | 15.87 | 15.75 |
| hsa-miR-525-3p_st | 12.79 | 12.70 | 12.60 | 7.24 | 10.37 | 10.06 |
| hsa-miR-523-star_st | 15.34 | 14.28 | 10.87 | 12.75 | 12.38 | 15.30 |
| hsa-miR-523_st | 11.73 | 21.24 | 15.89 | 15.06 | 11.69 | 15.04 |
| hsa-miR-518f-star_st | 23.89 | 21.53 | 25.00 | 24.25 | 26.89 | 34.93 |
| hsa-miR-518f_st | 12.18 | 13.09 | 14.82 | 16.22 | 13.57 | 11.48 |
| hsa-miR-520b_st | 14.40 | 16.13 | 14.10 | 13.21 | 16.68 | 14.92 |
| hsa-miR-518b_st | 12.24 | 12.36 | 13.10 | 11.75 | 17.54 | 19.27 |
| hsa-miR-526a_st | 16.42 | 17.29 | 12.25 | 14.47 | 13.56 | 19.11 |
| hsa-miR-520c-5p_st | 16.72 | 15.08 | 17.67 | 15.08 | 15.90 | 19.22 |
| hsa-miR-520c-3p_st | 17.19 | 17.64 | 19.87 | 16.09 | 14.40 | 13.55 |
| hsa-miR-518c-star_st | 23.22 | 24.34 | 19.89 | 21.94 | 20.82 | 24.35 |
| hsa-miR-518c_st | 13.16 | 18.36 | 17.98 | 12.80 | 16.71 | 10.52 |
| hsa-miR-524-5p_st | 15.90 | 14.80 | 16.03 | 12.87 | 13.86 | 23.76 |
| hsa-miR-524-3p_st | 15.90 | 15.87 | 12.34 | 15.96 | 19.10 | 13.50 |
| hsa-miR-517-star_st | 12.27 | 18.77 | 14.61 | 16.90 | 16.47 | 19.10 |
| hsa-miR-517a_st | 10.31 | 13.24 | 15.85 | 16.15 | 13.83 | 15.29 |
| hsa-miR-519d_st | 14.93 | 11.96 | 13.77 | 13.69 | 15.58 | 15.99 |
| hsa-miR-521_st | 15.11 | 16.95 | 15.98 | 10.84 | 14.41 | 19.03 |
| hsa-miR-520d-5p_st | 14.20 | 20.09 | 16.70 | 16.63 | 17.12 | 16.99 |
| hsa-miR-520d-3p_st | 15.07 | 19.06 | 16.23 | 14.82 | 15.95 | 13.35 |
| hsa-miR-517b_st | 15.43 | 17.06 | 14.86 | 18.90 | 14.45 | 12.62 |
| hsa-miR-520g_st | 14.19 | 19.08 | 16.25 | 12.24 | 14.89 | 14.23 |
| hsa-miR-516b_st | 19.11 | 18.14 | 15.43 | 19.24 | 15.15 | 15.56 |
| hsa-miR-516b-star_st | 15.90 | 14.71 | 14.13 | 13.18 | 19.12 | 15.30 |
| hsa-miR-518e-star_st | 14.76 | 13.72 | 11.96 | 13.18 | 14.78 | 16.55 |
| hsa-miR-518e_st | 11.53 | 10.20 | 15.31 | 15.25 | 16.49 | 16.45 |
| hsa-miR-518a-5p_st | 16.13 | 12.10 | 16.50 | 16.02 | 17.06 | 21.06 |
| hsa-miR-518a-3p_st | 14.86 | 11.47 | 15.19 | 15.22 | 14.69 | 13.20 |
| hsa-miR-518d-5p_st | 15.97 | 17.58 | 17.01 | 17.79 | 17.69 | 16.63 |
| hsa-miR-518d-3p_st | 16.70 | 12.25 | 13.47 | 11.78 | 16.52 | 12.64 |
| hsa-miR-517c_st | 15.07 | 12.23 | 14.59 | 10.11 | 17.01 | 11.56 |
| hsa-miR-520h_st | 14.99 | 10.77 | 11.59 | 16.13 | 22.61 | 11.43 |
| hsa-miR-522-star_st | 13.80 | 14.60 | 15.72 | 10.72 | 17.62 | 16.37 |
| hsa-miR-522_st | 18.39 | 15.44 | 18.18 | 15.78 | 18.51 | 18.72 |
| hsa-miR-519a-star_st | 15.88 | 11.98 | 15.61 | 16.83 | 16.00 | 9.24 |
| hsa-miR-519a_st | 16.98 | 13.73 | 16.50 | 15.07 | 15.43 | 17.63 |
| hsa-miR-527_st | 14.30 | 14.10 | 14.40 | 15.06 | 14.34 | 15.76 |
| hsa-miR-516a-5p_st | 18.28 | 10.59 | 15.49 | 16.30 | 14.26 | 16.88 |
| hsa-miR-516a-3p_st | 14.10 | 13.03 | 10.76 | 15.30 | 13.41 | 12.30 |
| hsa-miR-499-5p_st | 15.04 | 21.43 | 17.61 | 16.18 | 15.90 | 19.53 |
| hsa-miR-499-3p_st | 15.76 | 15.87 | 16.77 | 17.79 | 18.08 | 19.01 |
| hsa-miR-500_st | 179.42 | 191.58 | 138.71 | 85.59 | 166.48 | 164.63 |
| hsa-miR-500-star_st | 248.14 | 312.57 | 246.17 | 160.65 | 286.92 | 249.99 |
| hsa-miR-501-5p_st | 63.40 | 65.92 | 47.22 | 44.47 | 55.92 | 40.67 |
| hsa-miR-501-3p_st | 126.90 | 158.66 | 134.41 | 81.17 | 121.26 | 116.20 |
| hsa-miR-502-5p_st | 17.63 | 22.44 | 19.67 | 18.24 | 17.75 | 15.03 |
| hsa-miR-502-3p_st | 331.90 | 388.42 | 239.01 | 171.05 | 246.82 | 273.37 |
| hsa-miR-503_st | 123.83 | 151.86 | 98.91 | 141.28 | 525.94 | 571.78 |
| hsa-miR-504_st | 18.40 | 21.09 | 17.17 | 16.31 | 18.08 | 13.50 |
| hsa-miR-505-star_st | 110.90 | 188.94 | 120.84 | 62.62 | 131.08 | 122.61 |
| hsa-miR-505_st | 22.33 | 54.82 | 44.77 | 40.54 | 23.57 | 24.16 |
| hsa-miR-513a-5p_st | 19.11 | 18.29 | 19.17 | 17.39 | 20.09 | 15.37 |
| hsa-miR-513a-3p_st | 17.15 | 18.91 | 13.32 | 17.23 | 15.80 | 16.83 |
| hsa-miR-506_st | 11.01 | 18.25 | 17.64 | 13.29 | 13.01 | 13.21 |
| hsa-miR-507_st | 15.59 | 13.07 | 14.39 | 14.17 | 13.14 | 18.00 |
| hsa-miR-508-5p_st | 12.51 | 12.24 | 13.63 | 13.68 | 10.81 | 11.24 |
| hsa-miR-508-3p_st | 11.76 | 15.04 | 13.29 | 12.62 | 18.12 | 13.04 |
| hsa-miR-509-5p_st | 13.49 | 16.95 | 17.06 | 15.18 | 12.35 | 11.59 |
| hsa-miR-509-3p_st | 18.03 | 18.43 | 20.75 | 18.73 | 19.35 | 18.96 |
| hsa-miR-510_st | 16.98 | 15.60 | 10.36 | 13.82 | 13.96 | 18.13 |
| hsa-miR-514_st | 14.26 | 18.88 | 12.60 | 14.75 | 13.45 | 15.99 |
| hsa-miR-532-5p_st | 373.26 | 439.54 | 326.84 | 198.50 | 340.40 | 373.25 |
| hsa-miR-532-3p_st | 116.18 | 143.66 | 146.17 | 90.83 | 119.25 | 120.65 |
| hsa-miR-455-5p_st | 18.92 | 25.34 | 30.47 | 39.24 | 28.31 | 22.61 |
| hsa-miR-455-3p_st | 512.18 | 695.10 | 812.05 | 771.16 | 1106.34 | 1138.91 |
| hsa-miR-539_st | 16.25 | 19.25 | 19.36 | 28.09 | 16.31 | 19.16 |
| hsa-miR-544_st | 19.20 | 17.93 | 15.52 | 18.13 | 12.76 | 15.33 |
| hsa-miR-545-star_st | 20.47 | 15.77 | 14.35 | 18.17 | 11.79 | 14.21 |
| hsa-miR-545_st | 18.02 | 14.01 | 17.79 | 12.37 | 13.86 | 15.99 |
| hsa-miR-487b_st | 429.28 | 581.00 | 716.27 | 725.28 | 686.55 | 660.30 |
| hsa-miR-551a_st | 16.39 | 17.50 | 13.22 | 11.18 | 13.89 | 17.35 |
| hsa-miR-552_st | 15.93 | 10.54 | 15.76 | 14.52 | 15.08 | 19.34 |
| hsa-miR-553_st | 17.13 | 14.84 | 14.97 | 16.55 | 14.54 | 12.73 |
| hsa-miR-554_st | 10.75 | 9.48 | 14.08 | 12.63 | 15.68 | 16.54 |
| hsa-miR-92b-star_st | 132.97 | 77.27 | 262.69 | 237.34 | 131.83 | 131.95 |
| hsa-miR-92b_st | 243.77 | 318.26 | 196.11 | 213.27 | 296.18 | 214.66 |
| hsa-miR-555_st | 13.70 | 14.30 | 18.32 | 14.49 | 14.25 | 15.53 |
| hsa-miR-556-5p_st | 11.82 | 13.70 | 16.25 | 14.28 | 16.39 | 15.16 |
| hsa-miR-556-3p_st | 14.84 | 15.04 | 12.60 | 13.97 | 15.82 | 14.16 |
| hsa-miR-557_st | 12.46 | 11.56 | 12.58 | 12.10 | 19.26 | 12.95 |
| hsa-miR-558_st | 14.84 | 14.56 | 16.25 | 14.92 | 13.85 | 16.96 |
| hsa-miR-559_st | 15.90 | 16.95 | 13.30 | 17.42 | 17.60 | 15.67 |
| hsa-miR-561_st | 16.97 | 12.73 | 15.32 | 13.44 | 16.64 | 17.10 |
| hsa-miR-562_st | 14.42 | 15.63 | 15.77 | 15.26 | 15.95 | 18.07 |
| hsa-miR-563_st | 15.22 | 15.84 | 13.02 | 15.34 | 16.97 | 17.49 |
| hsa-miR-564_st | 28.54 | 18.91 | 21.38 | 20.03 | 19.07 | 21.75 |
| hsa-miR-566_st | 15.90 | 19.10 | 13.51 | 14.72 | 11.63 | 12.34 |
| hsa-miR-567_st | 20.66 | 19.59 | 16.19 | 16.34 | 16.48 | 17.49 |
| hsa-miR-568_st | 12.61 | 14.17 | 15.81 | 15.49 | 13.27 | 13.39 |
| hsa-miR-551b-star_st | 27.65 | 38.33 | 30.36 | 28.53 | 30.80 | 27.15 |
| hsa-miR-551b_st | 32.59 | 26.01 | 28.38 | 26.57 | 20.67 | 18.41 |
| hsa-miR-569_st | 15.08 | 13.51 | 18.11 | 13.55 | 17.02 | 13.04 |
| hsa-miR-570_st | 15.33 | 18.03 | 16.25 | 15.77 | 18.27 | 15.36 |
| hsa-miR-571_st | 17.87 | 17.51 | 17.18 | 19.82 | 17.02 | 20.27 |
| hsa-miR-572_st | 32.58 | 31.92 | 105.42 | 53.37 | 25.96 | 30.84 |
| hsa-miR-573_st | 11.80 | 12.65 | 12.51 | 14.14 | 11.22 | 14.67 |
| hsa-miR-574-5p_st | 272.52 | 415.90 | 245.93 | 168.25 | 282.74 | 310.05 |
| hsa-miR-574-3p_st | 3349.02 | 4203.58 | 3632.92 | 2286.22 | 3674.06 | 3836.91 |
| hsa-miR-575_st | 14.23 | 19.10 | 23.20 | 19.97 | 15.60 | 13.84 |
| hsa-miR-576-5p_st | 14.84 | 12.46 | 16.12 | 14.98 | 16.58 | 12.14 |
| hsa-miR-576-3p_st | 16.31 | 17.90 | 15.04 | 15.68 | 13.19 | 14.46 |
| hsa-miR-577_st | 14.81 | 14.80 | 15.37 | 12.03 | 13.70 | 14.95 |
| hsa-miR-578_st | 18.47 | 19.59 | 20.97 | 18.78 | 21.62 | 26.50 |
| hsa-miR-579_st | 14.28 | 16.14 | 12.33 | 18.64 | 17.89 | 13.23 |
| hsa-miR-580_st | 15.52 | 16.19 | 17.17 | 17.12 | 17.58 | 16.11 |
| hsa-miR-581_st | 14.22 | 16.44 | 13.38 | 15.29 | 12.64 | 15.31 |
| hsa-miR-582-5p_st | 14.84 | 15.83 | 12.42 | 12.88 | 13.88 | 14.78 |
| hsa-miR-582-3p_st | 15.36 | 15.01 | 14.94 | 13.66 | 13.90 | 13.60 |
| hsa-miR-583_st | 11.97 | 14.93 | 12.65 | 16.41 | 16.20 | 10.60 |
| hsa-miR-584_st | 20.40 | 26.24 | 18.34 | 17.59 | 17.90 | 24.59 |
| hsa-miR-585_st | 14.37 | 15.60 | 17.65 | 16.44 | 16.64 | 12.26 |
| hsa-miR-548a-3p_st | 26.04 | 21.25 | 21.90 | 20.01 | 24.02 | 25.73 |
| hsa-miR-586_st | 15.22 | 15.26 | 11.87 | 13.51 | 16.37 | 14.37 |
| hsa-miR-587_st | 15.61 | 16.95 | 15.80 | 14.87 | 15.67 | 14.04 |
| hsa-miR-548b-5p_st | 16.60 | 18.32 | 18.11 | 15.96 | 14.08 | 14.11 |
| hsa-miR-548b-3p_st | 17.41 | 12.97 | 12.23 | 14.47 | 15.95 | 12.03 |
| hsa-miR-588_st | 13.98 | 15.92 | 14.76 | 14.38 | 12.45 | 20.89 |
| hsa-miR-589_st | 20.19 | 15.87 | 18.72 | 17.45 | 15.95 | 18.65 |
| hsa-miR-589-star_st | 24.80 | 28.34 | 24.51 | 26.83 | 20.21 | 36.35 |
| hsa-miR-550_st | 22.62 | 24.67 | 20.38 | 20.70 | 30.54 | 18.53 |
| hsa-miR-550-star_st | 18.53 | 32.98 | 27.60 | 34.27 | 28.52 | 30.37 |
| hsa-miR-590-5p_st | 17.83 | 14.34 | 12.82 | 16.38 | 16.15 | 16.99 |
| hsa-miR-590-3p_st | 14.16 | 15.68 | 18.67 | 15.06 | 11.87 | 19.01 |
| hsa-miR-591_st | 17.09 | 8.94 | 13.37 | 10.69 | 13.45 | 12.08 |
| hsa-miR-592_st | 24.18 | 14.80 | 16.35 | 19.27 | 15.73 | 14.02 |
| hsa-miR-593-star_st | 8.26 | 11.86 | 9.02 | 7.25 | 13.86 | 12.03 |
| hsa-miR-593_st | 11.91 | 12.29 | 15.38 | 18.30 | 17.29 | 17.71 |
| hsa-miR-595_st | 17.74 | 16.05 | 20.90 | 17.80 | 24.05 | 20.69 |
| hsa-miR-596_st | 15.15 | 12.68 | 14.32 | 8.83 | 11.14 | 13.45 |
| hsa-miR-597_st | 10.36 | 16.12 | 12.59 | 10.83 | 17.26 | 18.06 |
| hsa-miR-598_st | 14.87 | 14.81 | 13.52 | 16.65 | 14.40 | 14.96 |
| hsa-miR-599_st | 13.79 | 13.50 | 10.84 | 15.96 | 17.02 | 16.01 |
| hsa-miR-548a-5p_st | 18.16 | 19.99 | 19.83 | 16.90 | 16.40 | 22.15 |
| hsa-miR-600_st | 14.13 | 18.24 | 16.32 | 18.42 | 18.72 | 14.02 |
| hsa-miR-601_st | 16.98 | 22.87 | 22.23 | 15.92 | 17.97 | 23.20 |
| hsa-miR-602_st | 24.07 | 27.85 | 67.06 | 76.15 | 36.91 | 23.90 |
| hsa-miR-603_st | 20.52 | 16.20 | 18.67 | 18.84 | 10.72 | 15.99 |
| hsa-miR-604_st | 15.89 | 20.80 | 15.52 | 14.11 | 17.98 | 20.74 |
| hsa-miR-605_st | 16.61 | 20.76 | 16.25 | 14.56 | 20.50 | 15.73 |
| hsa-miR-606_st | 17.25 | 22.95 | 17.70 | 19.14 | 19.89 | 15.84 |
| hsa-miR-607_st | 14.93 | 13.99 | 14.70 | 18.64 | 15.90 | 14.25 |
| hsa-miR-608_st | 7.50 | 11.51 | 6.33 | 9.43 | 7.72 | 13.03 |
| hsa-miR-609_st | 13.79 | 15.01 | 19.00 | 16.41 | 14.55 | 15.08 |
| hsa-miR-610_st | 18.16 | 18.03 | 15.91 | 18.05 | 15.68 | 16.97 |
| hsa-miR-611_st | 6.44 | 8.56 | 8.75 | 4.99 | 7.98 | 13.99 |
| hsa-miR-612_st | 12.43 | 14.80 | 14.67 | 26.92 | 14.47 | 15.93 |
| hsa-miR-613_st | 12.75 | 15.55 | 17.01 | 15.96 | 13.36 | 14.76 |
| hsa-miR-614_st | 9.76 | 15.76 | 15.62 | 6.19 | 10.72 | 14.16 |
| hsa-miR-615-5p_st | 18.17 | 23.09 | 17.73 | 21.55 | 15.99 | 24.83 |
| hsa-miR-615-3p_st | 92.86 | 271.45 | 166.99 | 111.00 | 28.28 | 261.85 |
| hsa-miR-616-star_st | 19.87 | 18.03 | 18.31 | 16.90 | 16.18 | 13.00 |
| hsa-miR-616_st | 14.84 | 18.58 | 17.90 | 21.15 | 16.32 | 18.03 |
| hsa-miR-548c-5p_st | 11.86 | 17.51 | 11.84 | 12.24 | 12.00 | 17.14 |
| hsa-miR-548c-3p_st | 15.96 | 13.60 | 15.46 | 13.95 | 14.89 | 14.02 |
| hsa-miR-617_st | 19.15 | 20.36 | 18.23 | 19.70 | 26.76 | 20.54 |
| hsa-miR-618_st | 12.89 | 16.85 | 16.25 | 13.18 | 16.60 | 12.77 |
| hsa-miR-619_st | 12.66 | 12.92 | 12.86 | 12.50 | 12.75 | 10.28 |
| hsa-miR-620_st | 16.66 | 15.52 | 18.09 | 18.87 | 16.84 | 15.99 |
| hsa-miR-621_st | 15.90 | 12.94 | 17.08 | 15.02 | 17.02 | 16.08 |
| hsa-miR-622_st | 16.40 | 15.82 | 13.77 | 13.08 | 14.81 | 12.19 |
| hsa-miR-623_st | 14.75 | 13.63 | 22.86 | 14.01 | 16.12 | 16.20 |
| hsa-miR-624-star_st | 18.04 | 18.41 | 15.33 | 13.29 | 15.06 | 16.99 |
| hsa-miR-624_st | 13.79 | 13.19 | 15.05 | 14.10 | 18.38 | 14.23 |
| hsa-miR-625_st | 233.98 | 96.22 | 67.59 | 118.57 | 88.30 | 141.53 |
| hsa-miR-625-star_st | 15.34 | 16.75 | 16.31 | 18.00 | 19.13 | 15.10 |
| hsa-miR-626_st | 15.02 | 15.56 | 13.44 | 16.34 | 18.55 | 17.45 |
| hsa-miR-627_st | 16.68 | 23.47 | 14.80 | 17.17 | 14.42 | 10.20 |
| hsa-miR-628-5p_st | 18.11 | 25.03 | 17.17 | 23.39 | 21.20 | 21.93 |
| hsa-miR-628-3p_st | 49.02 | 95.49 | 83.54 | 72.20 | 57.53 | 66.63 |
| hsa-miR-629_st | 54.36 | 111.86 | 31.49 | 72.02 | 63.41 | 82.05 |
| hsa-miR-629-star_st | 42.04 | 44.52 | 29.26 | 32.57 | 33.77 | 37.64 |
| hsa-miR-630_st | 14.84 | 16.24 | 17.85 | 17.06 | 14.89 | 14.96 |
| hsa-miR-631_st | 12.15 | 7.07 | 13.27 | 10.22 | 12.60 | 12.01 |
| hsa-miR-33b_st | 12.45 | 19.05 | 15.65 | 17.01 | 13.86 | 14.87 |
| hsa-miR-33b-star_st | 12.36 | 10.05 | 9.66 | 6.01 | 9.71 | 14.30 |
| hsa-miR-632_st | 13.79 | 13.70 | 17.78 | 18.64 | 12.90 | 16.74 |
| hsa-miR-633_st | 17.73 | 17.68 | 15.99 | 13.86 | 14.64 | 18.94 |
| hsa-miR-634_st | 14.10 | 17.27 | 13.93 | 14.75 | 17.86 | 11.73 |
| hsa-miR-635_st | 15.34 | 15.15 | 15.90 | 13.78 | 16.81 | 20.82 |
| hsa-miR-636_st | 12.31 | 12.83 | 16.43 | 18.60 | 22.17 | 13.40 |
| hsa-miR-637_st | 14.54 | 17.20 | 17.56 | 17.71 | 24.52 | 18.00 |
| hsa-miR-638_st | 4625.65 | 3155.71 | 7475.24 | 5995.47 | 4591.50 | 4612.31 |
| hsa-miR-639_st | 23.79 | 22.58 | 26.68 | 27.64 | 25.38 | 25.42 |
| hsa-miR-640_st | 17.46 | 21.26 | 22.01 | 14.78 | 18.67 | 19.21 |
| hsa-miR-641_st | 19.64 | 16.90 | 15.87 | 21.98 | 18.64 | 20.51 |
| hsa-miR-642_st | 18.13 | 15.10 | 14.97 | 17.48 | 14.82 | 19.38 |
| hsa-miR-643_st | 16.98 | 14.70 | 19.48 | 17.79 | 17.02 | 14.00 |
| hsa-miR-644_st | 15.07 | 15.68 | 14.27 | 13.90 | 11.97 | 14.00 |
| hsa-miR-645_st | 11.17 | 12.12 | 17.70 | 12.91 | 16.05 | 14.69 |
| hsa-miR-646_st | 16.34 | 15.87 | 15.16 | 15.86 | 15.95 | 19.97 |
| hsa-miR-647_st | 20.51 | 21.18 | 20.42 | 16.01 | 21.10 | 21.41 |
| hsa-miR-648_st | 22.52 | 19.39 | 19.42 | 21.63 | 18.19 | 18.55 |
| hsa-miR-649_st | 18.04 | 16.03 | 16.60 | 14.66 | 15.08 | 18.79 |
| hsa-miR-650_st | 18.55 | 16.01 | 12.17 | 13.52 | 19.38 | 8.52 |
| hsa-miR-651_st | 16.68 | 16.44 | 15.58 | 14.54 | 15.95 | 15.99 |
| hsa-miR-652_st | 169.23 | 126.83 | 115.18 | 100.84 | 158.14 | 142.71 |
| hsa-miR-548d-5p_st | 17.85 | 19.43 | 16.91 | 18.64 | 17.89 | 16.27 |
| hsa-miR-548d-3p_st | 18.59 | 16.60 | 17.17 | 17.87 | 17.41 | 17.54 |
| hsa-miR-661_st | 9.62 | 7.83 | 10.75 | 5.76 | 14.26 | 8.88 |
| hsa-miR-662_st | 17.06 | 16.95 | 18.99 | 14.90 | 18.34 | 16.99 |
| hsa-miR-663_st | 1007.83 | 900.45 | 2061.62 | 1895.44 | 1604.23 | 1138.57 |
| hsa-miR-449b_st | 17.31 | 12.96 | 16.63 | 19.81 | 22.61 | 17.19 |
| hsa-miR-449b-star_st | 20.48 | 19.51 | 26.79 | 20.08 | 23.08 | 23.59 |
| hsa-miR-653_st | 14.23 | 16.43 | 21.18 | 15.49 | 13.33 | 17.74 |
| hsa-miR-411_st | 122.98 | 150.11 | 181.86 | 310.86 | 165.03 | 177.91 |
| hsa-miR-411-star_st | 24.63 | 33.54 | 31.94 | 49.98 | 31.12 | 30.85 |
| hsa-miR-654-5p_st | 61.86 | 67.00 | 90.36 | 69.20 | 76.24 | 73.62 |
| hsa-miR-654-3p_st | 52.54 | 83.01 | 112.51 | 158.30 | 72.25 | 83.08 |
| hsa-miR-655_st | 13.79 | 18.09 | 15.40 | 14.70 | 14.69 | 11.67 |
| hsa-miR-656_st | 18.33 | 12.48 | 15.62 | 18.38 | 20.23 | 15.50 |
| hsa-miR-549_st | 11.38 | 16.72 | 14.90 | 19.39 | 16.85 | 16.99 |
| hsa-miR-657_st | 9.26 | 13.32 | 11.14 | 8.48 | 13.14 | 11.89 |
| hsa-miR-658_st | 15.73 | 14.83 | 17.64 | 17.51 | 17.00 | 20.34 |
| hsa-miR-659_st | 22.14 | 18.76 | 17.35 | 13.18 | 19.17 | 14.93 |
| hsa-miR-660_st | 86.63 | 101.89 | 93.61 | 93.14 | 87.05 | 87.08 |
| hsa-miR-421_st | 48.54 | 132.58 | 107.88 | 101.45 | 92.60 | 124.69 |
| hsa-miR-542-5p_st | 54.54 | 88.90 | 73.39 | 58.29 | 146.31 | 169.98 |
| hsa-miR-542-3p_st | 16.27 | 17.41 | 17.06 | 16.76 | 17.03 | 16.42 |
| hsa-miR-758_st | 23.89 | 35.64 | 37.83 | 56.83 | 36.26 | 42.06 |
| hsa-miR-1264_st | 16.96 | 15.87 | 14.16 | 14.16 | 15.24 | 11.70 |
| hsa-miR-671-5p_st | 134.88 | 199.22 | 113.76 | 109.41 | 142.46 | 176.06 |
| hsa-miR-671-3p_st | 36.81 | 67.55 | 36.82 | 37.02 | 43.02 | 63.65 |
| hsa-miR-668_st | 17.03 | 23.17 | 21.81 | 26.69 | 25.10 | 26.46 |
| hsa-miR-767-5p_st | 13.52 | 16.32 | 19.13 | 16.73 | 15.37 | 19.30 |
| hsa-miR-767-3p_st | 13.55 | 20.20 | 16.88 | 17.13 | 14.13 | 15.55 |
| hsa-miR-1224-5p_st | 14.54 | 19.12 | 22.57 | 30.51 | 16.93 | 18.71 |
| hsa-miR-1224-3p_st | 15.60 | 15.58 | 14.58 | 13.28 | 17.20 | 16.67 |
| hsa-miR-320b_st | 8137.06 | 5897.26 | 4332.61 | 3462.77 | 7025.75 | 6224.71 |
| hsa-miR-320c_st | 6115.37 | 4851.62 | 3931.78 | 3283.43 | 6183.44 | 5057.70 |
| hsa-miR-1296_st | 22.22 | 53.72 | 25.35 | 46.70 | 45.80 | 81.99 |
| hsa-miR-1468_st | 16.35 | 16.99 | 14.82 | 17.73 | 14.97 | 15.43 |
| hsa-miR-1323_st | 22.43 | 21.69 | 14.41 | 18.64 | 16.40 | 14.66 |
| hsa-miR-1271_st | 232.65 | 393.22 | 189.54 | 205.82 | 260.70 | 325.72 |
| hsa-miR-1301_st | 159.02 | 119.53 | 78.11 | 54.74 | 128.86 | 117.72 |
| hsa-miR-454-star_st | 11.16 | 11.87 | 15.33 | 15.72 | 14.89 | 13.63 |
| hsa-miR-454_st | 15.78 | 14.50 | 17.28 | 15.97 | 20.76 | 19.01 |
| hsa-miR-1185_st | 14.84 | 13.32 | 15.99 | 15.02 | 14.93 | 14.90 |
| hsa-miR-449c_st | 12.48 | 13.03 | 12.73 | 13.42 | 12.73 | 13.03 |
| hsa-miR-449c-star_st | 15.31 | 14.08 | 14.19 | 15.95 | 17.02 | 15.81 |
| hsa-miR-1283_st | 18.72 | 12.05 | 16.00 | 14.67 | 15.95 | 12.57 |
| hsa-miR-769-5p_st | 67.21 | 93.05 | 53.31 | 62.18 | 83.69 | 105.38 |
| hsa-miR-769-3p_st | 21.18 | 53.44 | 33.51 | 33.95 | 55.87 | 40.48 |
| hsa-miR-766_st | 36.63 | 20.76 | 28.56 | 21.94 | 15.21 | 20.54 |
| hsa-miR-762_st | 1989.49 | 1645.90 | 4912.86 | 3708.96 | 3050.86 | 2558.92 |
| hsa-miR-802_st | 13.82 | 16.96 | 16.55 | 16.05 | 14.81 | 19.26 |
| hsa-miR-670_st | 12.08 | 18.31 | 16.33 | 13.41 | 15.27 | 16.27 |
| hsa-miR-1298_st | 19.73 | 17.44 | 13.95 | 18.46 | 15.22 | 11.64 |
| hsa-miR-2113_st | 14.37 | 21.53 | 19.29 | 18.06 | 19.36 | 17.09 |
| hsa-miR-761_st | 13.79 | 15.55 | 12.73 | 17.12 | 18.63 | 15.59 |
| hsa-miR-764_st | 11.87 | 13.53 | 11.70 | 9.84 | 16.52 | 15.24 |
| hsa-miR-759_st | 20.57 | 10.83 | 12.69 | 17.00 | 15.22 | 15.72 |
| hsa-miR-765_st | 13.75 | 15.10 | 19.05 | 15.23 | 17.26 | 18.15 |
| hsa-miR-770-5p_st | 18.09 | 22.86 | 18.88 | 29.25 | 28.07 | 20.48 |
| hsa-miR-675_st | 12.62 | 22.23 | 17.04 | 25.31 | 25.60 | 11.93 |
| hsa-miR-675-star_st | 15.98 | 17.80 | 20.04 | 18.36 | 15.74 | 16.23 |
| hsa-miR-298_st | 12.75 | 12.91 | 14.69 | 11.86 | 15.84 | 14.04 |
| hsa-miR-891a_st | 11.75 | 12.48 | 12.91 | 16.40 | 18.90 | 16.99 |
| hsa-miR-300_st | 15.48 | 21.57 | 18.59 | 12.61 | 12.60 | 14.79 |
| hsa-miR-886-5p_st | 1208.46 | 1391.69 | 1114.86 | 785.59 | 1353.27 | 2002.33 |
| hsa-miR-886-3p_st | 247.78 | 185.04 | 159.48 | 228.70 | 322.82 | 377.42 |
| hsa-miR-892a_st | 14.59 | 13.76 | 14.41 | 14.83 | 14.15 | 13.03 |
| hsa-miR-220b_st | 15.90 | 18.30 | 16.77 | 17.49 | 19.16 | 14.75 |
| hsa-miR-450b-5p_st | 17.67 | 13.95 | 13.68 | 17.73 | 18.85 | 20.80 |
| hsa-miR-450b-3p_st | 13.59 | 14.10 | 16.72 | 14.24 | 16.12 | 16.85 |
| hsa-miR-874_st | 89.34 | 104.83 | 123.10 | 70.42 | 94.26 | 64.11 |
| hsa-miR-890_st | 14.62 | 11.65 | 14.27 | 14.02 | 17.75 | 14.02 |
| hsa-miR-891b_st | 17.15 | 18.53 | 21.19 | 19.03 | 11.65 | 16.24 |
| hsa-miR-220c_st | 13.79 | 15.10 | 17.84 | 10.76 | 15.89 | 16.57 |
| hsa-miR-888_st | 15.34 | 15.25 | 11.73 | 12.69 | 13.45 | 18.90 |
| hsa-miR-888-star_st | 10.82 | 15.84 | 12.67 | 11.91 | 12.64 | 15.82 |
| hsa-miR-892b_st | 19.34 | 15.73 | 12.45 | 16.63 | 15.14 | 16.79 |
| hsa-miR-541-star_st | 18.36 | 18.12 | 17.08 | 17.43 | 14.82 | 21.60 |
| hsa-miR-541_st | 16.66 | 15.08 | 19.83 | 17.34 | 18.26 | 18.75 |
| hsa-miR-889_st | 13.55 | 14.61 | 16.24 | 15.77 | 15.37 | 15.78 |
| hsa-miR-875-5p_st | 12.90 | 14.80 | 11.43 | 14.27 | 12.91 | 14.26 |
| hsa-miR-875-3p_st | 24.56 | 18.48 | 18.54 | 17.47 | 17.61 | 18.00 |
| hsa-miR-876-5p_st | 17.52 | 13.88 | 12.67 | 14.85 | 15.88 | 17.21 |
| hsa-miR-876-3p_st | 15.62 | 14.52 | 20.79 | 13.81 | 18.39 | 16.06 |
| hsa-miR-708_st | 159.64 | 101.75 | 137.58 | 505.13 | 243.35 | 557.79 |
| hsa-miR-708-star_st | 16.18 | 14.28 | 14.60 | 14.63 | 16.60 | 12.54 |
| hsa-miR-147b_st | 10.96 | 13.09 | 17.84 | 12.24 | 17.82 | 15.00 |
| hsa-miR-190b_st | 18.00 | 19.23 | 14.46 | 15.91 | 17.08 | 15.01 |
| hsa-miR-744_st | 504.00 | 767.12 | 476.46 | 422.02 | 801.11 | 823.86 |
| hsa-miR-744-star_st | 16.04 | 26.93 | 16.23 | 26.48 | 17.02 | 17.09 |
| hsa-miR-885-5p_st | 45.24 | 35.85 | 68.19 | 47.09 | 31.36 | 39.18 |
| hsa-miR-885-3p_st | 20.39 | 31.74 | 57.08 | 67.85 | 26.23 | 28.26 |
| hsa-miR-877_st | 88.40 | 63.60 | 54.97 | 38.71 | 92.11 | 55.39 |
| hsa-miR-877-star_st | 13.79 | 17.82 | 17.77 | 14.85 | 15.16 | 17.82 |
| hsa-miR-887_st | 25.10 | 51.44 | 41.74 | 44.93 | 49.61 | 45.54 |
| hsa-miR-665_st | 81.51 | 44.58 | 39.07 | 56.02 | 57.19 | 47.44 |
| hsa-miR-873_st | 16.84 | 12.73 | 16.17 | 14.92 | 12.48 | 19.05 |
| hsa-miR-543_st | 68.86 | 62.00 | 70.67 | 62.26 | 42.74 | 79.92 |
| hsa-miR-374b_st | 18.77 | 20.21 | 19.92 | 26.58 | 18.72 | 15.00 |
| hsa-miR-374b-star_st | 17.68 | 15.74 | 15.03 | 13.04 | 15.90 | 16.65 |
| hsa-miR-760_st | 16.45 | 26.84 | 21.71 | 23.81 | 24.39 | 29.51 |
| hsa-miR-301b_st | 20.23 | 14.58 | 17.17 | 17.57 | 18.08 | 12.80 |
| hsa-miR-216b_st | 17.03 | 22.09 | 19.79 | 19.70 | 21.29 | 21.08 |
| hsa-miR-208b_st | 18.30 | 15.78 | 13.61 | 14.80 | 14.95 | 13.53 |
| hsa-miR-920_st | 22.93 | 20.97 | 14.01 | 23.41 | 19.91 | 18.48 |
| hsa-miR-921_st | 10.63 | 22.22 | 19.08 | 18.16 | 16.22 | 14.52 |
| hsa-miR-922_st | 14.55 | 13.35 | 10.93 | 10.10 | 13.57 | 15.00 |
| hsa-miR-924_st | 14.01 | 17.07 | 17.30 | 16.45 | 16.07 | 15.99 |
| hsa-miR-509-3-5p_st | 19.74 | 16.23 | 20.73 | 16.18 | 18.08 | 17.29 |
| hsa-miR-933_st | 22.98 | 23.97 | 40.75 | 36.93 | 28.08 | 31.57 |
| hsa-miR-934_st | 16.73 | 16.40 | 17.02 | 17.22 | 21.76 | 17.73 |
| hsa-miR-935_st | 16.80 | 14.40 | 19.54 | 14.29 | 13.86 | 20.04 |
| hsa-miR-936_st | 22.41 | 17.75 | 16.78 | 20.27 | 17.79 | 16.14 |
| hsa-miR-937_st | 11.07 | 14.84 | 13.85 | 9.89 | 15.66 | 11.25 |
| hsa-miR-938_st | 20.36 | 16.33 | 18.11 | 16.14 | 14.49 | 15.03 |
| hsa-miR-939_st | 38.87 | 31.65 | 41.50 | 47.38 | 49.75 | 29.06 |
| hsa-miR-940_st | 43.75 | 22.04 | 21.84 | 19.51 | 24.55 | 24.26 |
| hsa-miR-941_st | 29.00 | 20.33 | 23.78 | 31.92 | 25.60 | 26.55 |
| hsa-miR-942_st | 11.65 | 12.61 | 16.01 | 12.24 | 14.89 | 12.92 |
| hsa-miR-943_st | 23.59 | 20.96 | 35.60 | 34.64 | 19.16 | 20.95 |
| hsa-miR-944_st | 17.72 | 14.24 | 15.64 | 16.61 | 12.03 | 11.21 |
| hsa-miR-297_st | 18.15 | 14.84 | 13.78 | 19.99 | 16.07 | 19.91 |
| hsa-miR-1178_st | 11.96 | 13.74 | 16.51 | 17.15 | 17.41 | 13.30 |
| hsa-miR-1179_st | 15.04 | 17.14 | 11.99 | 15.17 | 14.11 | 15.62 |
| hsa-miR-1180_st | 142.27 | 198.05 | 153.98 | 78.45 | 183.40 | 156.41 |
| hsa-miR-1181_st | 9.85 | 14.60 | 14.58 | 20.23 | 18.01 | 16.12 |
| hsa-miR-1182_st | 9.36 | 17.21 | 14.66 | 13.18 | 12.00 | 20.42 |
| hsa-miR-1183_st | 14.31 | 14.35 | 18.75 | 13.07 | 16.99 | 14.83 |
| hsa-miR-1184_st | 29.17 | 24.26 | 28.63 | 45.56 | 82.68 | 24.32 |
| hsa-miR-1225-5p_st | 56.64 | 59.08 | 67.40 | 65.28 | 49.90 | 65.00 |
| hsa-miR-1225-3p_st | 9.62 | 13.72 | 12.99 | 12.25 | 11.05 | 14.15 |
| hsa-miR-1226-star_st | 27.42 | 18.49 | 23.50 | 26.66 | 43.96 | 31.69 |
| hsa-miR-1226_st | 21.41 | 26.41 | 24.49 | 24.68 | 22.35 | 26.76 |
| hsa-miR-1227_st | 18.98 | 19.10 | 14.95 | 16.83 | 14.73 | 16.44 |
| hsa-miR-1228-star_st | 859.77 | 621.85 | 1785.44 | 1862.70 | 1165.38 | 973.08 |
| hsa-miR-1228_st | 46.85 | 36.94 | 35.58 | 19.70 | 31.29 | 19.27 |
| hsa-miR-1229_st | 14.19 | 17.02 | 10.92 | 14.84 | 15.56 | 14.25 |
| hsa-miR-1231_st | 188.22 | 127.64 | 496.07 | 395.40 | 185.09 | 232.51 |
| hsa-miR-1233_st | 20.48 | 10.84 | 12.93 | 17.43 | 15.92 | 15.89 |
| hsa-miR-1234_st | 14.31 | 21.13 | 10.59 | 10.58 | 23.39 | 20.04 |
| hsa-miR-1236_st | 17.07 | 19.10 | 13.24 | 11.89 | 18.08 | 13.01 |
| hsa-miR-1237_st | 18.20 | 20.18 | 19.95 | 20.89 | 19.89 | 13.52 |
| hsa-miR-1238_st | 16.80 | 18.52 | 17.17 | 21.12 | 18.44 | 30.02 |
| hsa-miR-1200_st | 9.43 | 11.97 | 12.22 | 12.30 | 15.03 | 16.09 |
| hsa-miR-1201_st | 13.45 | 18.29 | 22.33 | 14.40 | 16.60 | 19.65 |
| hsa-miR-1202_st | 24.19 | 23.74 | 27.60 | 23.83 | 25.48 | 21.08 |
| hsa-miR-1203_st | 11.21 | 15.12 | 11.94 | 12.24 | 16.24 | 12.88 |
| hsa-miR-663b_st | 22.73 | 18.17 | 5.92 | 7.12 | 12.68 | 10.66 |
| hsa-miR-1204_st | 12.81 | 12.61 | 14.41 | 11.31 | 14.68 | 17.82 |
| hsa-miR-1205_st | 9.87 | 16.72 | 13.32 | 16.19 | 15.62 | 17.77 |
| hsa-miR-1206_st | 16.22 | 14.70 | 13.77 | 13.46 | 12.95 | 16.27 |
| hsa-miR-1207-5p_st | 304.47 | 269.99 | 299.09 | 313.66 | 283.21 | 275.80 |
| hsa-miR-1207-3p_st | 16.38 | 18.34 | 16.91 | 11.40 | 18.12 | 13.98 |
| hsa-miR-1208_st | 22.51 | 27.69 | 45.59 | 29.53 | 20.57 | 29.59 |
| hsa-miR-548e_st | 15.90 | 14.34 | 16.25 | 18.64 | 18.89 | 15.17 |
| hsa-miR-548j_st | 14.84 | 12.23 | 13.51 | 16.42 | 13.81 | 23.43 |
| hsa-miR-1285_st | 28.92 | 24.07 | 25.46 | 29.60 | 26.05 | 27.09 |
| hsa-miR-1286_st | 16.16 | 13.13 | 18.80 | 15.84 | 13.06 | 13.60 |
| hsa-miR-1287_st | 18.66 | 44.76 | 31.80 | 42.32 | 44.33 | 59.94 |
| hsa-miR-1289_st | 12.60 | 13.67 | 15.31 | 15.86 | 14.64 | 10.80 |
| hsa-miR-1290_st | 23.35 | 23.53 | 30.19 | 41.40 | 17.02 | 20.27 |
| hsa-miR-1291_st | 15.12 | 10.91 | 10.76 | 10.15 | 13.02 | 8.66 |
| hsa-miR-548k_st | 18.73 | 14.47 | 14.65 | 16.67 | 14.26 | 17.71 |
| hsa-miR-1293_st | 14.54 | 15.59 | 10.83 | 16.59 | 12.68 | 9.07 |
| hsa-miR-1294_st | 18.68 | 18.81 | 18.94 | 20.57 | 20.87 | 16.61 |
| hsa-miR-1295_st | 10.83 | 13.01 | 14.38 | 15.02 | 15.91 | 10.89 |
| hsa-miR-1297_st | 11.71 | 15.83 | 13.51 | 14.66 | 21.07 | 20.11 |
| hsa-miR-1299_st | 13.53 | 15.70 | 15.03 | 15.87 | 14.50 | 17.17 |
| hsa-miR-548l_st | 20.20 | 18.33 | 16.94 | 19.76 | 19.16 | 22.04 |
| hsa-miR-1302_st | 12.85 | 15.00 | 14.81 | 11.42 | 12.95 | 15.85 |
| hsa-miR-1303_st | 23.73 | 20.86 | 20.26 | 24.73 | 14.89 | 23.20 |
| hsa-miR-1304_st | 17.37 | 20.10 | 16.07 | 19.80 | 17.59 | 19.01 |
| hsa-miR-1305_st | 20.95 | 18.11 | 13.38 | 15.96 | 11.91 | 18.85 |
| hsa-miR-1243_st | 16.89 | 13.45 | 19.07 | 15.03 | 15.91 | 14.93 |
| hsa-miR-548f_st | 16.94 | 16.95 | 17.17 | 16.81 | 17.03 | 16.99 |
| hsa-miR-1244_st | 19.92 | 16.03 | 16.50 | 18.77 | 16.92 | 14.12 |
| hsa-miR-1245_st | 18.61 | 15.99 | 15.33 | 15.02 | 14.89 | 13.62 |
| hsa-miR-1246_st | 65.07 | 42.36 | 67.25 | 116.49 | 22.05 | 22.15 |
| hsa-miR-1247_st | 8.56 | 10.53 | 4.13 | 5.08 | 6.50 | 12.45 |
| hsa-miR-1248_st | 15.88 | 15.72 | 14.09 | 16.19 | 13.80 | 18.31 |
| hsa-miR-1249_st | 17.52 | 17.51 | 17.56 | 15.96 | 20.17 | 15.63 |
| hsa-miR-1250_st | 15.21 | 15.97 | 11.87 | 15.38 | 20.03 | 13.95 |
| hsa-miR-1251_st | 15.44 | 12.43 | 15.95 | 16.74 | 13.71 | 13.74 |
| hsa-miR-1253_st | 16.65 | 14.26 | 17.09 | 17.45 | 12.86 | 13.44 |
| hsa-miR-1254_st | 20.19 | 19.10 | 17.85 | 18.36 | 23.93 | 18.60 |
| hsa-miR-1255a_st | 22.89 | 12.68 | 15.37 | 17.80 | 17.16 | 18.05 |
| hsa-miR-1256_st | 15.34 | 18.94 | 15.02 | 14.75 | 12.15 | 14.44 |
| hsa-miR-1257_st | 19.59 | 17.58 | 21.79 | 16.16 | 19.53 | 17.39 |
| hsa-miR-1258_st | 16.78 | 12.76 | 17.87 | 17.15 | 12.27 | 12.84 |
| hsa-miR-1259_st | 13.17 | 17.98 | 17.50 | 16.90 | 17.78 | 16.71 |
| hsa-miR-1260_st | 35.77 | 33.31 | 43.34 | 35.05 | 56.22 | 35.33 |
| hsa-miR-548g_st | 14.74 | 11.10 | 16.27 | 14.01 | 15.65 | 16.12 |
| hsa-miR-1261_st | 15.54 | 13.41 | 13.17 | 14.60 | 12.91 | 13.46 |
| hsa-miR-1262_st | 16.53 | 15.14 | 16.35 | 14.32 | 21.12 | 20.75 |
| hsa-miR-1263_st | 25.48 | 21.96 | 15.39 | 22.85 | 22.04 | 23.99 |
| hsa-miR-548n_st | 14.79 | 19.10 | 15.33 | 18.78 | 20.21 | 17.41 |
| hsa-miR-548m_st | 14.16 | 9.90 | 17.63 | 13.25 | 15.99 | 15.18 |
| hsa-miR-1265_st | 23.28 | 19.03 | 18.02 | 20.43 | 16.17 | 16.44 |
| hsa-miR-548o_st | 15.29 | 16.24 | 16.74 | 16.30 | 14.60 | 16.66 |
| hsa-miR-1266_st | 13.51 | 18.07 | 16.01 | 17.93 | 20.21 | 16.99 |
| hsa-miR-1267_st | 15.09 | 19.56 | 19.08 | 17.66 | 22.59 | 15.99 |
| hsa-miR-1268_st | 206.07 | 224.24 | 234.47 | 300.94 | 252.68 | 181.45 |
| hsa-miR-1269_st | 13.21 | 10.91 | 11.23 | 11.31 | 8.63 | 9.15 |
| hsa-miR-1270_st | 24.09 | 20.17 | 21.93 | 21.16 | 24.89 | 22.15 |
| hsa-miR-1272_st | 19.23 | 15.90 | 19.98 | 18.66 | 20.29 | 17.80 |
| hsa-miR-1273_st | 10.53 | 16.91 | 11.64 | 15.34 | 12.83 | 16.65 |
| hsa-miR-1274a_st | 54.63 | 39.12 | 46.03 | 50.73 | 65.95 | 31.19 |
| hsa-miR-548h_st | 15.20 | 14.06 | 13.38 | 14.32 | 16.22 | 14.84 |
| hsa-miR-1275_st | 330.15 | 176.43 | 98.78 | 74.80 | 138.92 | 109.62 |
| hsa-miR-1276_st | 11.71 | 14.35 | 17.92 | 11.11 | 14.63 | 13.06 |
| hsa-miR-302e_st | 15.21 | 17.38 | 12.85 | 15.17 | 21.35 | 16.99 |
| hsa-miR-302f_st | 14.90 | 14.70 | 14.29 | 17.71 | 15.39 | 16.71 |
| hsa-miR-1277_st | 15.47 | 16.07 | 11.83 | 16.22 | 15.03 | 16.54 |
| hsa-miR-548p_st | 15.49 | 13.07 | 17.78 | 13.92 | 12.08 | 20.83 |
| hsa-miR-548i_st | 17.76 | 17.20 | 17.83 | 18.52 | 16.67 | 14.12 |
| hsa-miR-1278_st | 16.43 | 14.67 | 18.27 | 21.34 | 19.09 | 13.73 |
| hsa-miR-1279_st | 16.01 | 15.96 | 14.41 | 13.68 | 11.92 | 14.13 |
| hsa-miR-1274b_st | 33.51 | 30.43 | 46.80 | 47.24 | 68.89 | 39.50 |
| hsa-miR-1281_st | 137.27 | 135.81 | 102.89 | 54.26 | 79.66 | 96.99 |
| hsa-miR-1282_st | 12.93 | 14.47 | 18.00 | 12.88 | 15.45 | 14.52 |
| hsa-miR-1284_st | 13.40 | 15.87 | 15.78 | 14.59 | 20.79 | 14.96 |
| hsa-miR-1288_st | 16.39 | 11.09 | 10.24 | 13.90 | 13.39 | 11.54 |
| hsa-miR-1292_st | 18.04 | 29.58 | 24.76 | 13.08 | 18.58 | 19.79 |
| hsa-miR-1252_st | 17.48 | 14.46 | 17.69 | 13.61 | 16.38 | 14.04 |
| hsa-miR-1255b_st | 14.97 | 13.37 | 15.51 | 14.31 | 16.30 | 15.35 |
| hsa-miR-1280_st | 155.07 | 188.63 | 219.13 | 153.65 | 426.07 | 190.97 |
| hsa-miR-1308_st | 1186.37 | 1228.40 | 1097.72 | 861.67 | 1404.03 | 1404.66 |
| hsa-miR-664-star_st | 72.79 | 85.53 | 71.38 | 39.22 | 38.28 | 41.29 |
| hsa-miR-664_st | 26.21 | 21.16 | 19.36 | 24.47 | 18.34 | 20.94 |
| hsa-miR-1306_st | 16.86 | 19.70 | 17.34 | 20.80 | 17.47 | 24.49 |
| hsa-miR-1307_st | 558.78 | 539.41 | 657.66 | 375.79 | 565.53 | 650.19 |
| hsa-miR-513b_st | 16.46 | 17.23 | 17.45 | 11.74 | 15.78 | 16.73 |
| hsa-miR-513c_st | 13.76 | 14.30 | 14.97 | 14.97 | 15.59 | 14.05 |
| hsa-miR-1321_st | 16.60 | 16.08 | 18.61 | 15.76 | 13.83 | 17.49 |
| hsa-miR-1322_st | 14.73 | 16.89 | 14.01 | 15.48 | 11.64 | 13.97 |
| hsa-miR-720_st | 102.33 | 99.05 | 100.21 | 95.42 | 135.05 | 110.17 |
| hsa-miR-1197_st | 15.30 | 12.45 | 19.53 | 10.35 | 17.80 | 16.65 |
| hsa-miR-1324_st | 13.06 | 13.77 | 13.93 | 13.46 | 16.33 | 18.00 |
| hsa-miR-1469_st | 2762.17 | 2309.64 | 5290.07 | 4377.48 | 3467.35 | 2959.08 |
| hsa-miR-1470_st | 7.55 | 11.28 | 0.00 | 0.00 | 5.76 | 1.65 |
| hsa-miR-1471_st | 12.75 | 9.55 | 10.89 | 5.47 | 11.25 | 11.05 |
| hsa-miR-1537_st | 15.73 | 13.07 | 11.96 | 16.44 | 14.09 | 16.50 |
| hsa-miR-1538_st | 16.47 | 14.68 | 7.23 | 10.32 | 10.65 | 15.51 |
| hsa-miR-1539_st | 12.09 | 17.84 | 14.50 | 12.58 | 10.69 | 12.77 |
| hsa-miR-103-as_st | 25.39 | 20.58 | 20.97 | 16.48 | 20.17 | 22.15 |
| hsa-miR-320d_st | 1068.45 | 1105.57 | 1056.08 | 1048.72 | 1075.29 | 1086.03 |
| hsa-miR-1825_st | 50.38 | 41.41 | 36.14 | 30.62 | 31.33 | 36.00 |
| hsa-miR-1826_st | 12383.56 | 8722.54 | 9060.44 | 10485.11 | 10327.34 | 8780.17 |
| hsa-miR-1827_st | 25.11 | 15.87 | 20.11 | 20.19 | 23.07 | 25.19 |
| hsa-miR-1908_st | 1548.39 | 1135.73 | 3707.25 | 3254.04 | 2442.49 | 1892.94 |
| hsa-miR-1909-star_st | 51.60 | 53.11 | 73.84 | 38.99 | 47.20 | 40.54 |
| hsa-miR-1909_st | 66.29 | 73.90 | 206.01 | 221.40 | 146.18 | 120.00 |
| hsa-miR-1910_st | 205.21 | 120.71 | 219.18 | 152.63 | 197.09 | 163.77 |
| hsa-miR-1911_st | 15.04 | 24.43 | 11.68 | 12.99 | 9.85 | 12.22 |
| hsa-miR-1911-star_st | 19.64 | 17.72 | 13.83 | 14.23 | 18.21 | 12.21 |
| hsa-miR-1912_st | 14.91 | 15.04 | 13.46 | 16.85 | 19.16 | 13.93 |
| hsa-miR-1913_st | 19.63 | 16.45 | 29.48 | 18.62 | 13.72 | 18.00 |
| hsa-miR-1914_st | 11.11 | 8.32 | 7.16 | 5.56 | 7.69 | 12.97 |
| hsa-miR-1914-star_st | 12.44 | 16.19 | 18.36 | 24.63 | 13.52 | 15.63 |
| hsa-miR-1915-star_st | 13.61 | 11.24 | 11.15 | 10.22 | 12.64 | 10.74 |
| hsa-miR-1915_st | 5298.47 | 2986.66 | 8095.28 | 5674.82 | 4017.89 | 4386.97 |
| hsa-miR-1972_st | 104.51 | 56.88 | 45.54 | 40.60 | 35.61 | 54.71 |
| hsa-miR-1973_st | 45.61 | 29.92 | 42.99 | 34.28 | 28.07 | 21.14 |
| hsa-miR-1975_st | 5105.43 | 3011.88 | 3253.02 | 2103.31 | 3154.95 | 2274.77 |
| hsa-miR-1976_st | 14.75 | 20.20 | 13.16 | 16.94 | 17.93 | 15.57 |
| hsa-miR-1979_st | 273.26 | 404.36 | 395.82 | 450.38 | 548.27 | 498.77 |
| hsa-miR-2052_st | 16.57 | 17.79 | 17.81 | 17.33 | 18.54 | 12.16 |
| hsa-miR-2053_st | 25.75 | 15.36 | 15.30 | 16.77 | 15.03 | 10.41 |
| hsa-miR-2054_st | 89.16 | 91.77 | 63.50 | 48.00 | 78.91 | 91.56 |
| hsa-miR-2110_st | 82.08 | 77.86 | 61.89 | 47.23 | 92.54 | 78.86 |
| hsa-miR-2114_st | 12.07 | 16.54 | 15.78 | 12.60 | 15.76 | 13.41 |
| hsa-miR-2114-star_st | 16.70 | 12.78 | 12.21 | 12.49 | 20.63 | 15.80 |
| hsa-miR-2115_st | 13.90 | 19.70 | 14.33 | 13.78 | 15.26 | 15.38 |
| hsa-miR-2115-star_st | 17.47 | 16.60 | 14.31 | 19.93 | 18.20 | 15.99 |
| hsa-miR-2116_st | 16.03 | 18.19 | 15.48 | 20.16 | 17.18 | 15.07 |
| hsa-miR-2116-star_st | 14.84 | 16.44 | 15.33 | 21.71 | 18.08 | 12.91 |
| hsa-miR-2117_st | 13.82 | 10.37 | 14.77 | 15.00 | 15.16 | 11.69 |
| hsa-miR-548q_st | 13.57 | 12.65 | 19.24 | 15.96 | 16.52 | 14.02 |
| hsa-miR-2276_st | 19.11 | 21.35 | 25.30 | 16.74 | 17.56 | 20.90 |
| hsa-miR-2277_st | 59.83 | 33.73 | 77.48 | 60.88 | 56.69 | 32.64 |
| hsa-miR-2278_st | 21.31 | 24.99 | 21.37 | 18.70 | 14.31 | 29.04 |
| hsa-miR-711_st | 10.97 | 19.10 | 20.83 | 17.42 | 18.32 | 12.03 |
| hsa-miR-718_st | 21.03 | 12.29 | 27.61 | 24.59 | 44.11 | 24.23 |
| hsa-miR-2861_st | 3466.28 | 2464.45 | 6813.77 | 5284.73 | 3091.42 | 3589.67 |
| hsa-miR-2909_st | 13.07 | 14.08 | 14.05 | 16.18 | 15.19 | 20.50 |
| hsa-miR-3115_st | 15.59 | 17.80 | 16.05 | 15.78 | 14.62 | 20.88 |
| hsa-miR-3116_st | 16.98 | 12.17 | 12.60 | 13.98 | 9.94 | 13.50 |
| hsa-miR-3117_st | 19.52 | 14.54 | 18.76 | 16.03 | 19.49 | 19.01 |
| hsa-miR-3118_st | 15.94 | 14.98 | 19.24 | 14.54 | 12.07 | 14.02 |
| hsa-miR-3119_st | 14.84 | 14.34 | 11.60 | 15.27 | 13.05 | 13.80 |
| hsa-miR-3120_st | 14.19 | 20.91 | 18.11 | 18.04 | 16.81 | 18.19 |
| hsa-miR-3121_st | 16.00 | 13.72 | 18.03 | 16.76 | 17.32 | 15.46 |
| hsa-miR-3122_st | 12.75 | 17.87 | 12.10 | 20.44 | 21.45 | 15.58 |
| hsa-miR-3123_st | 15.73 | 13.98 | 14.11 | 12.90 | 20.00 | 15.65 |
| hsa-miR-3124_st | 36.74 | 27.65 | 54.23 | 75.69 | 67.94 | 36.52 |
| hsa-miR-548s_st | 16.98 | 16.24 | 12.45 | 19.03 | 11.78 | 18.69 |
| hsa-miR-3125_st | 11.97 | 15.66 | 15.60 | 15.04 | 16.53 | 10.78 |
| hsa-miR-3126-5p_st | 18.29 | 15.91 | 16.72 | 18.94 | 17.02 | 14.83 |
| hsa-miR-3126-3p_st | 14.95 | 13.72 | 14.78 | 14.00 | 15.78 | 16.99 |
| hsa-miR-3127_st | 17.51 | 15.67 | 19.44 | 17.47 | 18.58 | 20.93 |
| hsa-miR-3128_st | 22.03 | 22.32 | 22.03 | 29.07 | 23.15 | 25.54 |
| hsa-miR-3129_st | 13.35 | 16.44 | 15.32 | 12.24 | 19.87 | 11.93 |
| hsa-miR-3130-5p_st | 11.59 | 13.09 | 15.94 | 16.84 | 14.10 | 16.12 |
| hsa-miR-3130-3p_st | 14.44 | 18.95 | 15.17 | 18.39 | 16.98 | 17.92 |
| hsa-miR-3131_st | 13.57 | 16.13 | 13.69 | 15.89 | 14.22 | 12.61 |
| hsa-miR-3132_st | 20.50 | 22.31 | 16.51 | 15.31 | 10.60 | 15.77 |
| hsa-miR-3133_st | 21.31 | 16.12 | 15.48 | 18.26 | 16.04 | 18.00 |
| hsa-miR-378b_st | 29.62 | 23.13 | 23.30 | 25.79 | 24.17 | 22.51 |
| hsa-miR-3134_st | 15.52 | 19.85 | 16.18 | 16.46 | 16.46 | 16.84 |
| hsa-miR-3135_st | 16.34 | 17.08 | 10.81 | 14.58 | 16.42 | 20.04 |
| hsa-miR-466_st | 16.72 | 12.01 | 17.24 | 18.45 | 14.75 | 17.35 |
| hsa-miR-3136_st | 24.36 | 25.74 | 16.61 | 18.64 | 19.85 | 25.42 |
| hsa-miR-544b_st | 14.84 | 12.23 | 12.60 | 13.09 | 13.46 | 13.88 |
| hsa-miR-3137_st | 15.80 | 16.49 | 15.87 | 14.49 | 16.38 | 14.43 |
| hsa-miR-3138_st | 18.48 | 24.09 | 22.37 | 14.09 | 22.34 | 20.36 |
| hsa-miR-3139_st | 17.43 | 18.33 | 14.25 | 18.52 | 16.88 | 23.80 |
| hsa-miR-3140_st | 16.12 | 15.06 | 16.33 | 15.40 | 14.08 | 13.31 |
| hsa-miR-548t_st | 14.84 | 14.41 | 15.59 | 15.34 | 12.74 | 13.18 |
| hsa-miR-3141_st | 348.19 | 418.42 | 404.63 | 374.55 | 382.29 | 424.10 |
| hsa-miR-3142_st | 14.23 | 11.62 | 15.36 | 11.79 | 17.56 | 11.02 |
| hsa-miR-3143_st | 14.98 | 16.95 | 14.19 | 15.96 | 13.51 | 14.12 |
| hsa-miR-548u_st | 15.88 | 12.66 | 17.76 | 16.32 | 12.83 | 12.76 |
| hsa-miR-3144-5p_st | 13.15 | 15.44 | 14.83 | 12.87 | 16.73 | 14.55 |
| hsa-miR-3144-3p_st | 14.52 | 16.71 | 13.51 | 16.68 | 12.83 | 16.82 |
| hsa-miR-3145_st | 17.03 | 16.39 | 14.82 | 15.43 | 13.40 | 14.76 |
| hsa-miR-1273c_st | 16.59 | 16.53 | 19.29 | 18.97 | 19.21 | 18.69 |
| hsa-miR-3146_st | 14.84 | 14.82 | 17.81 | 15.16 | 11.69 | 15.71 |
| hsa-miR-3147_st | 15.84 | 16.16 | 17.19 | 18.73 | 16.67 | 16.93 |
| hsa-miR-548v_st | 19.89 | 15.78 | 12.67 | 11.77 | 14.43 | 12.29 |
| hsa-miR-3148_st | 15.90 | 14.77 | 18.11 | 14.17 | 19.90 | 19.58 |
| hsa-miR-3149_st | 15.38 | 14.80 | 20.82 | 16.57 | 15.99 | 17.05 |
| hsa-miR-3150_st | 11.77 | 12.54 | 11.03 | 10.37 | 11.76 | 12.25 |
| hsa-miR-3151_st | 13.80 | 16.95 | 18.11 | 26.49 | 17.23 | 17.15 |
| hsa-miR-3152_st | 14.63 | 15.12 | 14.54 | 15.12 | 12.83 | 16.99 |
| hsa-miR-3153_st | 17.73 | 15.24 | 14.97 | 15.19 | 15.34 | 15.81 |
| hsa-miR-3074_st | 15.94 | 16.98 | 14.80 | 18.74 | 17.79 | 16.46 |
| hsa-miR-3154_st | 18.17 | 20.19 | 18.38 | 21.81 | 20.89 | 20.05 |
| hsa-miR-3155_st | 13.88 | 17.96 | 13.51 | 10.63 | 15.02 | 18.85 |
| hsa-miR-3156_st | 19.45 | 20.07 | 19.71 | 19.28 | 18.27 | 17.82 |
| hsa-miR-3157_st | 15.70 | 16.67 | 21.53 | 13.84 | 16.73 | 18.00 |
| hsa-miR-3158_st | 12.75 | 14.80 | 10.76 | 10.80 | 14.23 | 19.18 |
| hsa-miR-3159_st | 12.87 | 14.97 | 15.19 | 14.89 | 15.00 | 17.66 |
| hsa-miR-3160_st | 16.67 | 16.66 | 16.93 | 12.39 | 15.97 | 17.89 |
| hsa-miR-3161_st | 10.87 | 11.31 | 13.42 | 13.55 | 13.40 | 12.59 |
| hsa-miR-3162_st | 57.84 | 52.00 | 52.49 | 52.04 | 52.66 | 44.78 |
| hsa-miR-3163_st | 17.41 | 17.27 | 15.25 | 18.00 | 15.96 | 19.01 |
| hsa-miR-3164_st | 12.41 | 14.13 | 15.14 | 17.30 | 14.56 | 14.43 |
| hsa-miR-3165_st | 15.86 | 14.75 | 14.91 | 15.35 | 16.44 | 13.31 |
| hsa-miR-3166_st | 15.54 | 11.99 | 14.41 | 15.07 | 12.34 | 12.85 |
| hsa-miR-1260b_st | 131.78 | 130.50 | 114.04 | 106.81 | 237.40 | 144.35 |
| hsa-miR-3167_st | 16.11 | 19.17 | 11.61 | 14.91 | 16.17 | 14.27 |
| hsa-miR-3168_st | 15.65 | 18.90 | 18.44 | 10.62 | 15.83 | 18.14 |
| hsa-miR-3169_st | 14.72 | 14.15 | 17.17 | 16.57 | 14.01 | 21.07 |
| hsa-miR-3170_st | 18.32 | 13.46 | 13.11 | 16.51 | 15.48 | 14.30 |
| hsa-miR-3171_st | 16.89 | 18.89 | 13.30 | 14.10 | 16.13 | 15.19 |
| hsa-miR-3172_st | 23.59 | 20.40 | 33.34 | 30.16 | 29.77 | 22.36 |
| hsa-miR-3173_st | 17.38 | 16.15 | 16.88 | 16.81 | 17.43 | 17.40 |
| hsa-miR-1193_st | 19.01 | 15.87 | 19.80 | 9.91 | 14.94 | 11.50 |
| hsa-miR-323b-5p_st | 14.90 | 18.90 | 16.62 | 17.24 | 15.04 | 15.49 |
| hsa-miR-323b-3p_st | 19.14 | 21.83 | 20.97 | 20.43 | 19.25 | 18.08 |
| hsa-miR-3174_st | 16.19 | 17.36 | 20.38 | 13.86 | 26.88 | 20.04 |
| hsa-miR-3175_st | 21.98 | 25.83 | 20.16 | 31.67 | 25.01 | 24.32 |
| hsa-miR-3176_st | 16.44 | 15.47 | 15.41 | 15.80 | 12.83 | 16.62 |
| hsa-miR-3177_st | 19.77 | 27.25 | 20.37 | 24.92 | 24.01 | 23.75 |
| hsa-miR-3178_st | 7314.99 | 3047.11 | 8184.13 | 6640.64 | 8584.86 | 4431.77 |
| hsa-miR-3179_st | 15.75 | 22.55 | 14.75 | 15.65 | 16.50 | 17.45 |
| hsa-miR-3180-5p_st | 5.34 | 7.89 | 4.09 | 0.93 | 3.83 | 8.75 |
| hsa-miR-3180-3p_st | 97.52 | 85.87 | 223.33 | 267.94 | 126.01 | 132.04 |
| hsa-miR-548w_st | 20.04 | 14.98 | 21.48 | 20.71 | 13.98 | 16.99 |
| hsa-miR-3181_st | 18.84 | 14.74 | 13.14 | 15.89 | 15.84 | 22.17 |
| hsa-miR-3182_st | 14.84 | 12.65 | 11.93 | 14.74 | 13.86 | 15.54 |
| hsa-miR-3183_st | 13.78 | 9.42 | 10.76 | 10.35 | 11.79 | 9.07 |
| hsa-miR-3184_st | 13.30 | 15.11 | 14.77 | 18.51 | 14.31 | 19.29 |
| hsa-miR-3185_st | 879.57 | 674.71 | 2232.55 | 1844.14 | 752.45 | 1078.42 |
| hsa-miR-3065-5p_st | 17.17 | 18.77 | 15.62 | 18.21 | 20.80 | 13.80 |
| hsa-miR-3065-3p_st | 15.79 | 15.13 | 13.54 | 15.57 | 21.26 | 18.55 |
| hsa-miR-3186-5p_st | 12.40 | 13.42 | 13.60 | 14.41 | 16.13 | 12.25 |
| hsa-miR-3186-3p_st | 13.38 | 19.51 | 13.51 | 16.03 | 13.86 | 13.76 |
| hsa-miR-3187_st | 29.98 | 25.30 | 74.65 | 64.97 | 50.12 | 58.86 |
| hsa-miR-3188_st | 79.03 | 72.44 | 298.54 | 116.79 | 75.65 | 90.87 |
| hsa-miR-3189_st | 14.01 | 15.79 | 11.92 | 13.86 | 11.79 | 13.23 |
| hsa-miR-320e_st | 198.72 | 267.47 | 216.05 | 210.93 | 243.16 | 187.98 |
| hsa-miR-3190-5p_st | 13.83 | 16.41 | 13.60 | 13.07 | 15.50 | 14.97 |
| hsa-miR-3190-3p_st | 18.89 | 18.36 | 20.21 | 17.96 | 16.25 | 16.25 |
| hsa-miR-3191_st | 12.57 | 12.00 | 14.40 | 15.47 | 16.08 | 14.30 |
| hsa-miR-3192_st | 12.76 | 15.90 | 18.02 | 19.04 | 19.16 | 12.97 |
| hsa-miR-3193_st | 14.84 | 14.74 | 17.07 | 15.48 | 20.38 | 23.20 |
| hsa-miR-3194_st | 10.75 | 14.80 | 15.44 | 7.49 | 10.05 | 9.74 |
| hsa-miR-3195_st | 418.22 | 244.25 | 758.30 | 415.03 | 296.18 | 257.48 |
| hsa-miR-3196_st | 4917.38 | 3236.39 | 7303.52 | 6196.59 | 5887.33 | 4496.83 |
| hsa-miR-548x_st | 20.19 | 19.04 | 16.55 | 19.01 | 21.06 | 21.15 |
| hsa-miR-3197_st | 37.49 | 53.35 | 54.21 | 91.59 | 105.90 | 87.11 |
| hsa-miR-3198_st | 16.51 | 17.52 | 16.40 | 17.67 | 17.03 | 14.41 |
| hsa-miR-3199_st | 15.71 | 19.35 | 15.40 | 19.13 | 18.14 | 19.74 |
| hsa-miR-3200_st | 16.77 | 24.38 | 17.32 | 16.78 | 12.31 | 15.84 |
| hsa-miR-3201_st | 44.20 | 32.32 | 27.98 | 43.19 | 54.87 | 26.81 |
| hsa-miR-514b-5p_st | 16.46 | 14.80 | 13.97 | 17.56 | 13.44 | 19.01 |
| hsa-miR-514b-3p_st | 14.84 | 12.81 | 14.79 | 12.57 | 15.32 | 13.03 |
| hsa-miR-3202_st | 18.22 | 15.89 | 15.33 | 17.79 | 13.88 | 21.32 |
| hsa-miR-1273d_st | 18.04 | 18.23 | 15.11 | 18.01 | 20.69 | 15.58 |
| hsa-miR-4295_st | 22.30 | 15.71 | 15.33 | 15.98 | 16.97 | 18.00 |
| hsa-miR-4296_st | 13.69 | 14.69 | 17.14 | 16.08 | 20.49 | 16.77 |
| hsa-miR-4297_st | 13.92 | 14.94 | 14.55 | 17.32 | 17.19 | 10.22 |
| hsa-miR-378c_st | 229.61 | 108.28 | 140.61 | 60.87 | 96.84 | 90.97 |
| hsa-miR-4293_st | 16.07 | 20.73 | 20.01 | 18.41 | 22.05 | 16.67 |
| hsa-miR-4294_st | 14.77 | 19.67 | 18.59 | 22.56 | 19.16 | 18.48 |
| hsa-miR-4301_st | 18.72 | 17.65 | 15.33 | 17.59 | 13.39 | 19.84 |
| hsa-miR-4299_st | 15.93 | 19.59 | 17.47 | 20.66 | 22.35 | 23.20 |
| hsa-miR-4298_st | 1139.78 | 1204.14 | 863.52 | 609.19 | 1116.35 | 1308.39 |
| hsa-miR-4300_st | 15.09 | 15.87 | 17.01 | 14.62 | 14.90 | 16.26 |
| hsa-miR-4304_st | 26.15 | 22.78 | 21.01 | 35.58 | 24.62 | 22.18 |
| hsa-miR-4302_st | 23.10 | 19.19 | 19.08 | 15.55 | 18.08 | 18.00 |
| hsa-miR-4303_st | 20.10 | 15.87 | 18.60 | 18.25 | 14.48 | 21.93 |
| hsa-miR-4305_st | 16.89 | 20.67 | 21.18 | 15.65 | 17.81 | 18.39 |
| hsa-miR-4306_st | 30.24 | 32.32 | 33.71 | 31.05 | 42.00 | 32.48 |
| hsa-miR-4309_st | 13.97 | 12.88 | 18.05 | 15.04 | 15.76 | 18.43 |
| hsa-miR-4307_st | 13.10 | 16.65 | 14.16 | 17.86 | 19.66 | 11.53 |
| hsa-miR-4308_st | 14.80 | 13.73 | 15.13 | 17.83 | 15.93 | 21.50 |
| hsa-miR-4310_st | 20.19 | 20.01 | 23.35 | 25.17 | 20.10 | 13.89 |
| hsa-miR-4311_st | 21.22 | 18.68 | 17.80 | 19.32 | 18.80 | 15.36 |
| hsa-miR-4312_st | 15.81 | 18.38 | 17.31 | 17.79 | 23.46 | 17.57 |
| hsa-miR-4313_st | 24.17 | 18.73 | 18.60 | 17.82 | 10.47 | 17.80 |
| hsa-miR-4315_st | 17.35 | 13.08 | 15.79 | 15.15 | 14.89 | 15.77 |
| hsa-miR-4316_st | 16.25 | 18.12 | 20.26 | 18.07 | 19.08 | 15.49 |
| hsa-miR-4314_st | 14.54 | 16.56 | 20.48 | 13.18 | 17.68 | 13.61 |
| hsa-miR-4318_st | 12.70 | 15.43 | 18.11 | 13.83 | 19.61 | 21.04 |
| hsa-miR-4319_st | 17.99 | 15.51 | 17.29 | 18.21 | 22.66 | 19.08 |
| hsa-miR-4320_st | 7.75 | 16.32 | 15.26 | 21.30 | 21.40 | 14.98 |
| hsa-miR-4317_st | 29.74 | 48.85 | 48.74 | 33.31 | 53.67 | 51.02 |
| hsa-miR-4322_st | 23.21 | 21.43 | 34.95 | 25.93 | 16.44 | 27.39 |
| hsa-miR-4321_st | 20.77 | 17.08 | 44.98 | 33.62 | 23.81 | 16.08 |
| hsa-miR-4323_st | 17.86 | 16.51 | 14.07 | 15.14 | 12.87 | 13.81 |
| hsa-miR-4324_st | 18.25 | 33.82 | 29.66 | 44.65 | 42.91 | 25.07 |
| hsa-miR-4256_st | 14.65 | 13.26 | 12.73 | 16.79 | 13.18 | 16.41 |
| hsa-miR-4257_st | 21.75 | 17.73 | 15.83 | 19.05 | 17.68 | 14.10 |
| hsa-miR-4258_st | 22.71 | 16.66 | 13.43 | 17.00 | 20.41 | 18.47 |
| hsa-miR-4259_st | 13.79 | 12.65 | 16.12 | 19.97 | 17.47 | 12.78 |
| hsa-miR-4260_st | 18.35 | 17.50 | 19.96 | 17.54 | 16.71 | 21.18 |
| hsa-miR-4253_st | 15.01 | 22.15 | 21.75 | 22.35 | 14.32 | 16.81 |
| hsa-miR-4251_st | 15.30 | 16.31 | 15.64 | 15.59 | 13.42 | 14.30 |
| hsa-miR-4254_st | 14.70 | 12.83 | 13.43 | 11.58 | 10.17 | 13.28 |
| hsa-miR-4255_st | 16.29 | 16.22 | 20.46 | 21.11 | 16.00 | 18.75 |
| hsa-miR-4252_st | 17.59 | 21.88 | 21.32 | 20.05 | 20.41 | 24.03 |
| hsa-miR-4325_st | 18.85 | 20.18 | 20.52 | 20.58 | 21.44 | 19.33 |
| hsa-miR-4326_st | 15.43 | 15.14 | 16.51 | 14.82 | 14.32 | 14.70 |
| hsa-miR-4327_st | 27.21 | 24.71 | 25.45 | 27.14 | 24.06 | 19.33 |
| hsa-miR-4261_st | 14.61 | 20.80 | 15.65 | 13.41 | 18.86 | 14.00 |
| hsa-miR-4265_st | 13.79 | 14.80 | 11.42 | 16.53 | 11.84 | 11.88 |
| hsa-miR-4266_st | 11.11 | 15.10 | 16.87 | 15.31 | 15.90 | 15.99 |
| hsa-miR-4267_st | 14.76 | 20.98 | 13.57 | 18.28 | 30.62 | 17.16 |
| hsa-miR-4262_st | 13.17 | 16.43 | 12.40 | 15.47 | 16.07 | 16.95 |
| hsa-miR-2355_st | 16.98 | 12.76 | 13.90 | 15.64 | 14.82 | 13.84 |
| hsa-miR-4268_st | 13.66 | 8.92 | 14.10 | 8.48 | 14.78 | 11.04 |
| hsa-miR-4269_st | 24.59 | 37.12 | 41.47 | 46.15 | 22.80 | 27.00 |
| hsa-miR-4263_st | 12.08 | 13.06 | 15.54 | 14.21 | 15.38 | 10.85 |
| hsa-miR-4264_st | 14.98 | 17.11 | 14.55 | 15.95 | 17.04 | 15.61 |
| hsa-miR-4270_st | 391.64 | 327.19 | 810.11 | 393.49 | 339.17 | 285.51 |
| hsa-miR-4271_st | 20.52 | 20.19 | 15.53 | 16.90 | 20.72 | 15.91 |
| hsa-miR-4272_st | 14.91 | 15.71 | 14.89 | 15.96 | 14.72 | 13.25 |
| hsa-miR-4273_st | 11.41 | 14.25 | 17.96 | 11.08 | 12.44 | 17.77 |
| hsa-miR-4276_st | 13.55 | 16.95 | 13.09 | 15.09 | 15.95 | 15.00 |
| hsa-miR-4275_st | 13.79 | 17.89 | 13.68 | 15.50 | 11.79 | 14.34 |
| hsa-miR-4274_st | 16.91 | 22.39 | 17.87 | 17.37 | 16.39 | 24.51 |
| hsa-miR-4281_st | 763.91 | 629.91 | 1538.54 | 836.59 | 654.91 | 538.95 |
| hsa-miR-4277_st | 14.24 | 14.35 | 15.09 | 15.75 | 15.22 | 22.04 |
| hsa-miR-4279_st | 15.95 | 21.96 | 20.69 | 17.30 | 18.08 | 18.27 |
| hsa-miR-4278_st | 13.22 | 13.63 | 15.76 | 14.99 | 13.10 | 14.24 |
| hsa-miR-4280_st | 16.09 | 13.63 | 15.80 | 14.53 | 15.46 | 13.43 |
| hsa-miR-4282_st | 16.39 | 15.82 | 13.77 | 14.49 | 19.32 | 14.98 |
| hsa-miR-4285_st | 14.98 | 19.10 | 19.67 | 24.06 | 21.20 | 19.01 |
| hsa-miR-4283_st | 16.98 | 16.71 | 15.26 | 16.90 | 17.80 | 20.69 |
| hsa-miR-4284_st | 220.60 | 131.05 | 70.38 | 126.36 | 146.59 | 112.51 |
| hsa-miR-4286_st | 47.44 | 42.60 | 61.24 | 54.05 | 65.60 | 47.45 |
| hsa-miR-4287_st | 16.17 | 16.16 | 20.04 | 12.24 | 15.59 | 21.08 |
| hsa-miR-4288_st | 29.86 | 42.74 | 35.62 | 33.23 | 40.35 | 31.45 |
| hsa-miR-4292_st | 12.86 | 16.99 | 23.00 | 30.10 | 24.29 | 15.69 |
| hsa-miR-4289_st | 13.69 | 19.29 | 17.27 | 21.54 | 16.40 | 23.25 |
| hsa-miR-4290_st | 20.36 | 18.50 | 24.68 | 14.10 | 17.17 | 16.65 |
| hsa-miR-4291_st | 17.37 | 18.63 | 13.58 | 18.54 | 14.61 | 17.60 |
| hsa-miR-4329_st | 43.50 | 48.62 | 40.96 | 38.79 | 53.52 | 47.24 |
| hsa-miR-4330_st | 11.90 | 13.08 | 17.29 | 12.70 | 17.93 | 15.99 |
| hsa-miR-500b_st | 12.99 | 15.36 | 14.41 | 16.06 | 11.79 | 13.90 |
| hsa-miR-4328_st | 16.96 | 16.58 | 17.09 | 16.14 | 18.26 | 17.87 |
| v11_hsa-miR-453_st | 14.95 | 16.77 | 12.25 | 11.28 | 12.86 | 10.26 |
| v11_hsa-miR-768-5p_st | 2737.42 | 2750.61 | 2836.10 | 2163.75 | 3037.52 | 2571.82 |
| v11_hsa-miR-768-3p_st | 1305.37 | 1362.38 | 1131.23 | 1051.76 | 1419.36 | 868.58 |
| v11_hsa-miR-923_st | 3656.30 | 2704.83 | 2081.37 | 4853.99 | 2943.47 | 2608.54 |
| v11_hsa-miR-1300_st | 21.93 | 15.87 | 23.78 | 20.69 | 25.95 | 13.11 |
