# Supplementary material for: miR-10a restores human mesenchymal stem cell differentiation by repressing KLF4
Source: J Cell Physiol. 2013 Aug 23;228(12):2324–36. doi: 10.1002/jcp.24402 (PMC4285942; doi:10.1002/jcp.24402)
Supplement: Supplementary file 8 — Table S5. Differential miRNA expression of hMSCs of different ages. [file jcp0228-2324-sd8.doc]

**Supplementary Table S5.** **Differential miRNA expression of hMSCs of different ages.**

| **Gene ID** | **q-value(%)** | **Fold Change** | **H78** | **H80** | **H75** | **H17** | **H20** | **H25** |
| --- | --- | --- | --- | --- | --- | --- | --- | --- |
| miR-196a | 0.00 | 6.54 | 45.68 | 178.44 | 270.12 | 25.44 | 18.71 | 16.56 |
| miR-486-5p | 0.00 | 2.92 | 129.66 | 207.80 | 115.49 | 85.84 | 39.48 | 37.00 |
| miR-664-star | 0.00 | 1.93 | 72.79 | 85.53 | 71.38 | 39.22 | 38.28 | 41.29 |
| miR-378-star | 0.00 | 1.87 | 40.18 | 42.68 | 38.46 | 23.26 | 21.99 | 19.85 |
| miR-708 | 0.00 | 0.32 | 159.64 | 101.75 | 137.58 | 505.13 | 243.35 | 557.79 |
| miR-10a | 0.00 | 0.26 | 36.62 | 49.74 | 45.76 | 222.40 | 165.42 | 130.90 |
| miR-3197 | 0.00 | 0.50 | 37.49 | 53.35 | 54.21 | 91.59 | 105.90 | 87.11 |
