# Supplementary material for: miR-10a restores human mesenchymal stem cell differentiation by repressing KLF4
Source: J Cell Physiol. 2013 Aug 23;228(12):2324–36. doi: 10.1002/jcp.24402 (PMC4285942; doi:10.1002/jcp.24402)
Supplement: Supplementary file 9 — Method S1. Luciferase reporter assay of miR-10b. [file jcp0228-2324-sd9.doc]

**Methods Supplementary 1. Luciferase reporter assay of miR-10b.**

A 100 ng aliquot of miR-10b mimics or scrambled control (GenePharma, Shanghai, China), 100 ng of pGL4.13-KLF4-3’UTR or pGL4.13-KLF4-3’UTR-mut containing the firefly luciferase reporter vector and 5 ng of the control vector containing Renilla luciferase (pRL-TK) were used to transfect 3x10^4^ hMSCs per well in a 48-well plate using a 0.5 µl aliquot of Lipofectamine 2000 (Invitrogen, San Diego, CA). HMSCs numbering 3x10^4^ were transfected by a 1 µl aliquot of Lipofectamine 2000 (Invitrogen, San Diego, CA), 200 ng of miR-10b inhibitor or inhibitor scrambled control (GenePharma, Shanghai, China), 200 ng of pGL4.13-KLF4-3’UTR or pGL4.13-KLF4-3’UTR-mut and 10 ng of pRL-TK. Firefly and Renilla luciferase activities were measured consecutively at 48h after transfection using the Dual-Luciferase system (Promega, Madison, WI) according to the manufacturer's instructions.
